# Key events in the process of sex determination and differentiation in early chicken embryos

**DOI:** 10.5713/ab.24.0679

**Published:** 2025-02-27

**Authors:** Xiaoqian Lv, Changhua Sun, Xin Liu, Guanzheng Liu, Wei Gong, Hongwu Qian, Zeyu Li, Jun Wu, Xilin Zhu, Jiuzhou Song, Yingjie Niu, Hongyan Sun, Wei Han, Guo hong Chen, Kai Jin, Bichun Li, Qisheng Zuo

**Affiliations:** 1 Joint International Research Laboratory of Agriculture and Agri-Product Safety of Ministry of Education of China, Yangzhou University, Yangzhou, China; 2 Key Laboratory of Animal Breeding Reproduction and Molecular Design for Jiangsu Province, College of Animal Science and Technology, Yangzhou University, Yangzhou, China; 3 Department of Food Technology, College of Biochemical Engineering, Yangzhou Polytechnic College, Yangzhou, China; 4 Department of Animal & Avian Sciences, University of Maryland, College Park, MD, USA; 5 Jiangsu Institute of Poultry Sciences/Poultry institute, Chinese Academy of Agricultural Sciences, Yangzhou, China; 6 College of Biotechnology, Jiangsu University of Science and Technology, Zhenjiang, China

**Keywords:** Chicken, DNA Methylation, Glycolysis, Histone Acetylation, Sex Determination

## Abstract

**Objective:**

Current understanding of sex determination and differentiation mechanisms during early chicken embryonic development remains incomplete. To address this, we applied RNA sequencing to identify male-female expression differences at critical developmental stages (E0 blastocysts, E3.5-E6.5 genital ridges, E18.5 gonads), focusing on glycolysis, histone acetylation, and DNA methylation. This approach aims to unravel key regulatory mechanisms and advance developmental biology insights.

**Methods:**

We analyzed molecular mechanisms of chicken sex determination at key stages (E0 blastocysts, E3.5-E6.5 genital ridges, E18.5 gonads) using RNA sequencing. Glycolysis, histone acetylation, and DNA methylation levels were assessed in embryonic stem cells and chicken embryonic fibroblasts. E18.5 gonads were treated with glycolytic activators (SB431542 and PD0325901 [2i]), a DNA demethylation activator (Vitamin C [Vc]), or an inhibitors of histone acetylation modification (valproic acid [VPA]). Sex-related gene expression, hormone levels, and gonad morphology were evaluated to determine treatment effects.

**Results:**

Key findings revealed that sex differences emerged as early as the blastocyst stage, intensified with embryonic development and were marked by a surge in sexually dimorphic gene expression. Gene Ontology and Kyoto encyclopedia of genes and genomes analyses highlighted the pivotal roles of energy metabolism and epigenetic modification process during this critical period. 2i, VC, or VPA interventions targeting E18.5 embryo gonads, induced a remarkable transformation of ovarian tissue into a testis-like structure, characterized by cortical thinning, medulla densification, downregulation of female-specific genes (FOXL2, WNT4), upregulation of male-specific genes (SOX9, AMH), and increased testosterone secretion. This phenotypic and molecular shift underscores the ability of metabolic and epigenetic modulators to reprogram ovarian development towards a male-like pattern, preserving male sexual characteristics.

**Conclusion:**

Our study establishes energy metabolism and epigenetic regulation as central drivers of avian sex determination. These findings advance understanding of vertebrate developmental biology and provide a framework for dissecting regulatory networks in avian sexual development.

## INTRODUCTION

Sex determination and differentiation are complex and fascinating developmental processes that have long been a research focus in the field of life sciences [1]. Studying the mechanisms of sex determination is not only crucial for understanding biological development and differentiation, but also effective in improving the production efficiency of economic animals and enriching biological research models. At present, the key genes for sex determination have not been identified, and the key regulators of sex determination and differentiation are not well understood. What’s more, the key regulators of sex determination and differentiation in chickens remain unclear, greatly limiting the research and development of sex control technologies in poultry production and affecting the use of chickens as models in basic research. Therefore, there is an urgent need to delve deeper into the mechanisms of sex determination in chickens.

In mammals, sex determination is primarily controlled by genetic factors [2]. For example, in humans and mice, the Y chromosome gene SRY/Sry causes the undifferentiated embryonic gonad to develop into a testis, resulting in a male phenotype. In the absence of SRY/Sry, the gonad develops into an ovary, leading to a female phenotype [3–5]. As research continues to deepen, various theories have emerged regarding the regulation of sex determination in poultry. Chickens have a sex chromosome composition of ZZ (male) and ZW (female), based on which the “Z-chromosome dosage effect” theory and the “W-chromosome dominance effect” theory have been developed. However, current studies have shown that the key molecules supporting these two hypotheses, namely the DMRT1 gene located on the Z-chromosome and the HINTW gene located on the W-chromosome, are not switch genes. When knocked out or interfered with, they do not result in complete sex reversal in chickens [6–8]. The emergence of gynandromorphic chickens has brought new insights into the study of sex determination in chickens: the “Somatic Sex Autonomous Identity in chicken” hypothesis [9]. This hypothesis suggests that the sex of chickens is autonomously determined by somatic cell genetic factors, independent of gonads and hormones [10]. But some studies suggest that sex hormones still play an important role in chicken sex determination [8,11]. These hypotheses attempt to explain the specific mechanisms of sex determination in birds. But no conclusive evidence has been found to determine which hypothesis is correct, or whether other unknown mechanisms of sex determination exist [8,11]. So far, only a series of important regulatory genes, such as SOX9 [11], DMRT1 [6], SPIN1Z [12], HINTW [7], FOXL2 [8] and so on. However, these genes are not the key ones [8], and their knockout or functional inhibition cannot completely reverse sex determination and differentiation in chickens [8]. SOX9 is a downstream gene of DMRT1, where DMRT1 induces and activates SOX9. When SOX9 is highly expressed in male embryos, it further promotes testicular differentiation [13,14]. DMRT1, located on the Z chromosome, is also not a switch gene. The knockout of DMRT1 cannot achieve sex reversal in Z^D−^W chickens [6]. These genes can indeed be used as potential candidate genes, but they are all downstream genes of the regulatory pathway, rather than switch genes. These researches indicates that the sex determination mechanism in chickens is relatively complex and influenced by multiple factors. Therefore, further exploration and understanding of the specific mechanisms underlying sex determination in chickens are urgently needed.

In this study, we aimed to identify the critical regulatory factors involved in sex determination and differentiation by extracting samples from early chicken embryos (E0 to E18.5 d) and conducting high-throughput RNA sequencing analysis. Our goal is to pioneer new avenues of research on sex determination and differentiation in chickens, providing novel insights into this complex process.

## MATERIALS AND METHODS

### Animals and ethical statement

Eggs were collected from Rugao Yellow chickens (a kind of egg-and-meat dual-purpose breed of chickens) (Poultry Institute, Chinese Academy of Agricultural Sciences, Yangzhou City, China). All embryos were incubated under conditions of 37.8°C and 65% relative humidity.

The animal experiments were approved by the Institutional Animal Care and Use Committee of the Yangzhou University Animal Experiments Ethics Committee (Permit Number: SYXK [Su] IACUC 2012-0029). All experimental procedures were performed in accordance with the Regulations for the Administration of Affairs Concerning Experimental Animals approved by the State Council of the People’s Republic of China.

### Cell Isolation and culture

The isolation and culture methods for chicken embryonic stem cells (ESC) and chicken embryonic fibroblasts (CEFs) followed previous descriptions [14,15]. Briefly, ESCs were isolated from the blastocyst cells of the x-stage (when the embryo develops to the Eyal-Giladi and Kochav stage X, blastodermal cells could be isolate [stage X embryo]) fertilized egg embryo region [16,17]. The isolated blastocyst cells were cultured at 37°C with 5% CO_2_ for 24 hours to purify them, using ESC medium. CEFs were isolated and cultured from 9-day-old fertilized eggs (chicken embryos hatched for 9 days, [E9]). After removing the head, tail, limbs, and internal organs of the chicken embryos, they were digested with trypsin, centrifuged at 200×g for 6 minutes at room temperature. The isolated CEFs were cultured and purified in Dulbecco’s Modified Eagle Medium (DMEM) medium (37°C, 5% CO_2_) (Gibico, Grand Island, NY, USA) containing 10% fetal bovine serum (FBS) for 24 h.

The collection of embryonic gonad tissues was conducted in three distinct stages. In the primary stage (E0, HH stage 1), blastocyst tissues were collected. Progressing to the onset stage (E3.5 to E6.5, HH stage 21 to HH stage 30), genital ridge tissues were harvested. Finally, in the final stage (E18.5, HH stage 44), testicular and ovarian tissues were obtained. The extraction of E0 embryonic tissues followed the same protocol as described previously for blastocyst tissues [14,16,18]. At E3.5 to E6.5 days, eggs were cleaned and sterilized. After opened eggshell, the embryo was gently removed using a pair of forceps. Under a stereomicroscope, the ventral side of the embryo was carefully incised using ophthalmic scissors, and the genital ridge was identified and separated using ophthalmic forceps. The isolated genital ridge tissues were rinsed in phosphate-buffered saline (PBS). At E18.5 d, the abdominal cavity of the chick embryo was opened, the testicular or ovarian tissues on both sides were clamped and separated using ophthalmic forceps, ensuring the removal of any attached tissues during the separation process. The isolated tissues were transferred to sample collection tubes containing TRIzol and appropriately labeled. The first wash solution containing blastocyst tissues of different sexes and blood samples from embryos at various stages were collected for subsequent sex identification.

### Sex determination of chicken embryo samples by polymerase chain reaction

Chicken sex determination was conducted through a polymerase chain reaction (PCR)-based detection method as previously described in the literature [19]. Mighty Amp DNA Polymerase was employed to amplify the CHD1 gene located on the sex chromosomes (Z/W). The PCR amplification products were then verified through gel electrophoresis. Primers were designed based on the genomic sequence of chicken CHD1 gene on sex chromosomes (CHD1-Z, chrZ:51359549-51400046; CHD1-W, chrW: 4989932-5105612, galGal6a, UCSC). The lengths of our amplified products using our primers for CHD1 are 580 base pair (bp) (chrZ: 51387236-51387815) on Z chromosome and 434 bp (chrW: 5019696-5020129) on W chromosome. The primer sequences were as follows: CHD1-Forward: CTGCGAGAAC GTGGCAACAGAGT; CHD1-Reverse: ATTGAAATGAT CCAGTGCTTG.

### Tissue RNA extraction, purification and sequencing

After sex identification, the identified tissue samples were grouped into 12 groups (total 36 samples) for the rest of the RNA extraction and sequencing according to developmental time and sex, and the groupings and naming are shown in Table 1, with three replicates set in each group.

Total RNA from each group was extracted using the TRIzol reagent (Tiangen, Beijing, China). DNase I (Takara, Dalian, China), which is free of RNase, was added to the reaction mixture for 10 minutes to effectively remove genomic DNA. The purity and concentration of the RNA were then determined using a NanoDrop 1000 spectrophotometer (Thermo Scientific, Waltham, MA, USA). The integrity of the RNA was further evaluated using an Agilent 2100 Bioanalyzer (Agilent Technologies, Santa Clara, CA, USA). Subsequently, the extracted RNA was reverse-transcribed and subjected to next-generation sequencing. Twelve RNA libraries were constructed according to the Illumina mRNA-seq sequencing protocol, with three replicates per group. The qualified RNA samples were sequenced using Illumina technology by Shanghai OE Biotech Co., Ltd. (Shanghai, China). The TruSeq Stranded mRNA LT Sample Prep Kit was employed for library preparation of the samples, and sequencing was conducted on the Illumina HiSeq X Ten sequencing platform. Detailed steps can be referred to the library preparation process instructions for the Illumina HiSeq X sequencing platform on the Illumina official website (https://www.illumina.com/systems/sequencing-platforms/hiseq-x). The prepared libraries underwent paired-end sequencing on the Illumina HiSeq X Ten with a read length of 150 bp. The sequencing depth was 6 G.

### Differential expression analysis and functional enrichment

The analysis methods for the second-generation sequencing data followed the descriptions provided in a previous study [19]. For the Raw Reads corresponding to each group of samples obtained by Illumina sequencing, the storage format is fastq. The Trimmomatic software [20] (Version 0.36, http://www.usadellab.org/cms/?page=trimmomatic) is used for processing (mainly including adapter removal, removal of low - quality reads and removal of low - quality bases from the 3 - end and 5 - end in different ways), and the processed data is statistically analyzed using RSeQC software [21,22] (Version 2.6.4, http://rseqc.sourceforge.net/) and FastQC software [23] (Version 0.11.5, https://www.bioinformatics.babraham.ac.uk/projects/fastqc/). The main original sequencing amount, effective sequencing amount, Q30, guanine-cytosine content, Supplement 1). After comprehensive evaluation, Clean Reads are obtained for the next experiment. The reference genome used in this alignment is the chicken reference genome GRCg6a (GCF_000002315.6, https://www.ncbi.nlm.nih.gov/assembly/GCF_000002315.6), and the alignment software is HISAT2 [24, 25] (Version 2.2.1.0, http://daehwankimlab.github.io/hisat2/). The obtained Clean Reads are aligned with the reference gene. The order and parameters of alignment: hisat2 -x genome.index -1 RD1 -2 RD2 -p 12 --rna-strandness RF --fr --summary-file sample.summary --new-summary -S sample.sam. Gene expression levels were quantified by calculating the fragments per kilobase of transcript per million mapped reads (FPKM) values. Heatmap and volcano cluster analysis were performed using the OE Biotech Cloud Platform (https://cloud.oebiotech.com/task/) to conduct enrichment analysis of differentially expressed genes (DEGs). The expression of each differential gene presented in the heatmap is the mean of three repeated results of the experimental group, and the heatmap default method for row normalization was z-score normalization. Upset graph is a kind of graph used to visually represent the overlap of a set of elements. Compared with venn graph, it can draw an unlimited number of sets. And this upset graph was performed using the OECloud tools at https://cloud.oebiotech.com. And upset analyses were employed to analyze the intersection of DEGs at different developmental stages between female and male. GraphPad Prism 8 software was employed for the analysis of expression patterns of DEGs.

### Chick embryo gonads isolation and culture *in vitro*

Sex gonads were isolated from chicken embryos ranging from incubation to E18.5. After cleaning the surface, the eggshell was cracked open with ophthalmic tweezers on a sterile ultra-clean table, picked out the eggs and collected the ovaries and bilateral testes, and then washed gonads by PBS for 3 times. Under a stereomicroscope, excess tissue was carefully removed using fine forceps. The cleaned gonads (ovaries and testes) were placed in a 24-well culture plate, with one gonads tissue per well. Each well was filled with 300 μL of DMEM (Hyclone, Logan, UT, USA) containing 10% FBS (Gibco, Grand Island, NY, USA) and 2% chicken serum (Gibco). The gonads were cultured for 6 days. To investigate the effects of glycolysis and epigenetic modifications (DNA methylation and histone acetylation) on chicken embryonic sex determination, glycolytic activators (2i: 3 μM SB431542 and 1 μM PD0325901, SB431542, HY-10431; PD0325901, HY-10254; MCE, Monmouth Junction, NJ, USA) [15], DNA methylation inhibitors (50 μg/mL vitamin C [Vc]), and histone deacetylase inhibitors (1 mM VPA) were added to the culture medium of the chicken embryonic gonad tissues *in vitro*. Every other day, replace with fresh 300 μL of culture medium (containing the corresponding small molecule compounds) for a continuous culture period of 6 days. The control group gonads were cultured in tissue culture medium without any compound treatment.

### Quantitative real-time polymerase chain reaction

After treating chicken gonads with 2i, Vc, or VPA, gonads were homogenized in TRIzol Reagent (DP424; QIAGEN, Beijing, China) and total RNA were isolated accordingly.

Quantitative real-time (qRT)-PCR was performed using the FastKing one-step RT-PCR kit with SYBR green (KR123; QIAGEN). The mRNA levels of relevant genes were detected using the CFX-Connect RT PCR Detection System (7500fast; Bio-rad, California, USA). The qRT-PCR primer sequences are listed in Table 2 (the approximate size ranges of the expected amplicons: Foxl2 (NM_001012612): 127 bp; Sox9 (NM_204281): 161 bp; AMH (NM_205030): 129 bp; WNT4 (NM_204783): 147 bp; β-actin (NM_205518.2): 169 bp). The qRT-PCR program included an initial denaturation step at 95°C for 30 seconds, followed by 40 cycles of 95°C for 10 seconds and 60°C for 30 seconds. The results were quantified using the 2^−ΔΔCt^ method, normalizing the data relative to β-Actin [18,26,27]. Three biological replicates were performed for this qRT-PCR experiment.

### Glucose uptake assay

Cells were seeded at 1×10^4^ cells per well in a 96-well plate with a black transparent bottom. Untreated cells served as an internal control. Subsequently, 100 μM of 2-NBDG (Thermofisher) and 100 μL of glucose-free medium (Solarbio, Beijing, China) were added, and the plate was incubated for 30 min at 37°C in a 5% CO_2_ environment, protected from light. Fluorescence was measured at excitation 465 nm and emission 540 nm using a fluorescence microplate reader (Tecan, Männedorf, Switzerland).

### Measurement of lactic acid production

Lactic acid is not only the end product of glycolysis, but also plays an important role in energy regulation and signal transduction [28]. The lactic acid assay kit (provided by Nanjing Jiancheng Bioengineering Institute, Nanjing, China) was utilized to determine the amount of lactic acid production. Following the manufacturer’s instructions, the supernatant from the induced culture medium was collected for lactic acid detection. Absorbance was measured using a fluorescence microplate reader (Tecan). The lactic acid production (mM) was calculated using the formula: 3×(Optical density [OD] of the sample group−OD of the blank control group)/(OD of the standard–OD of the blank control group).

### DNA methylation level analysis

DNA methylation level was detected by Dot blot assay and the experimental method was according to the previous description with some modification [29]. Collect both male and female ESCs, after extracted and purified DNA by DNA extraction kit, ddH_2_O was employed to dilute DNA to 100 ng/μL and the DNA samples were incubated at 95°C for 10 min. The DNA samples were quickly placed into ice, and each sample was diluted into different gradient samples (300 ng, 200 ng and 100 ng of DNA mass) with a volume of 5 μL. Drop the DNA samples on the nylon membrane, air drying after 15 min, Ultraviolet crosslinking at 1,200 J/m^2^ for 2 h. After blocking membranes by skimmed milk powder, 5-Hydroxymethylcytosine (5-hmC) Polyclonal Antibody was used as primary antibody. The secondary antibody was added and incubated for 2 h on a shaker at room temperature. The membranes were finally immersed in the reaction system (1:1 mixing BeyoECL Moon A and B) in the dark for 1 min and imaged with Bio-Rad ChemiDoc Imaging System (Bio-Rad,). The calculation method of relative levels of 5hmc: $\frac{A+B+C}{3}$:1 (A: Female_300 ng_/Male_300 ng_; B: Female_200 ng_/Male_200 ng_; C: Female_100 ng_/Male_100 ng_). Three biological replicates were performed for this Dot blot experiment.

### Histone acetylation level analysis

The histone acetylation enzyme (HAT) enzyme-linked immunosorbent assay (ELISA) kit (YIYAN Biological Technology, Shanghai, China) was used for the measurement of histone acetylation levels. According to the manufacturer instructions, collect the supernatant sample after cell homogenate and add the samples to coated microplate. After washing and HAT ELISA kit reagent treatment, the histone acetylation levels of those samples were measured by a fluorescence microplate reader (Tecan) according to manufacturer’s instructions.

### Paraffin sectioning and hematoxylin-eosin (H&E) staining

Firstly, adjust the morphology of the isolated gonadal tissue under a stereo microscope (MVX10; Olympus, Tokyo, Japan) and remove any additional attached tissues. The isolated gonadal tissue is then preserved in 4% paraformaldehyde for 24 hours before being transferred to 70% ethanol. Subsequently, gradient dehydration was performed using ethanol concentrations ranging from low to high, followed by clearance with xylene and embedding in paraffin. The embedded tissue was continuously sectioned into slices with a thickness of 6 μm. The slices were then dewaxed using xylene and infiltrated with a gradient of ethanol concentrations from high to low. Subsequently, after HE staining and dehydration, the tissue blocks were mounted. Figures were taken under an optical microscope (DMC6200; Leica, Weztlar, Germany).

### The level of estradiol and testosterone assay

The intact gonad tissue was collected in a 1.5 mL centrifuge tube containing 350 μL of PBS. After being fully homogenized, the samples were centrifuged at 900×g for a duration of 30 minutes, and the supernatant was then carefully collected. Three female and three male samples were randomly selected from each of the Blank group and treatment groups for gonad samples. The estradiol and testosterone concentration of these samples was measured by ELISA kit according to manufacturer’s instructions (F8599-A and F8573-A; YiLi, Shanghai, China).

## Statistical analysis

Each test was repeated three times, and the results were expressed as mean±standard error. SPSS was used to compare the differences between groups for statistical significance. Differences between each group were considered as significant using Student t-test (* p<0.05, statistically significant; ** p<0.01 statistically very significant), and GraphPad Prism8 software was used for mapping.

## RESULTS

### Successfully constructed a transcriptome library for analysis of chicken sex determination and differentiation-related genes

**Figure 1:**
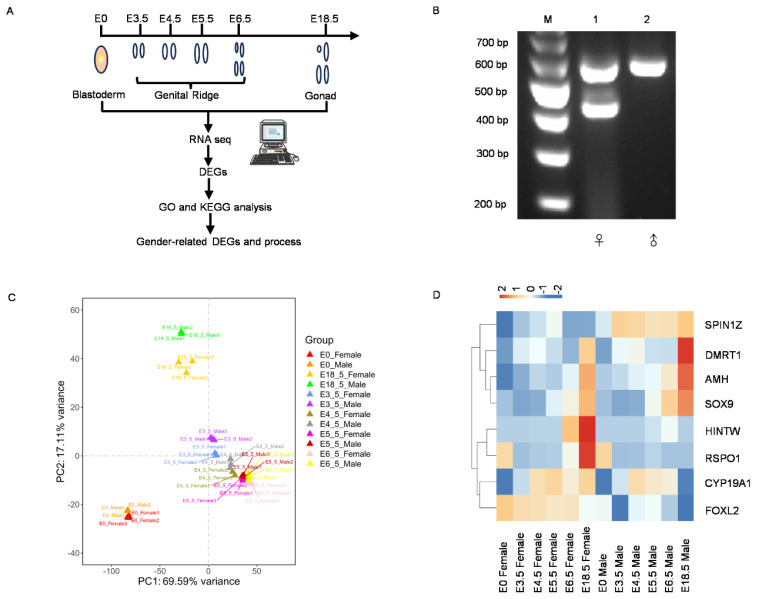
Sample collection arrangements and RNA-Seq data evaluation. (A) Detailed sample grouping and collection process. (B) Partial sex identification results. M, marker; Lane1, female genital ridge tissues at E6.5 d; Lane 2, male genital ridge tissues at E6.5 d. (C) PCA analysis of samples from different stages. (D) Heatmap analysis of sex-related genes at various stages. The detailed Gene IDs of sex-related genes and their mean FPKM values during different developmental stages corresponding to the heatmap is already listed in Table 3. The default row normalization mode is z-score normalization. DEGs, differentially expressed genes; GO, Gene Ontology; KEGG, Kyoto encyclopedia of genes and genomes; bp, base pair; PC, principal component; PCA, principal component analysis; FPKM, fragments per kilobase of transcript per million mapped reads.

[Table t3-ab-24-0679][Table t4-ab-24-0679][Table t5-ab-24-0679][Table t6-ab-24-0679]To elucidate the key regulatory networks involved in chicken sex determination and differentiation, blastosphere (E0), genital ridge (E3.5 d, E4.5 d, E5.5 d, and E6.5 d) and gonads (E18.5 d) were collected at different developmental stages using PCR-based sex identification methods and extracted RNA for transcriptome sequencing (Figure 1A; Supplement 2). Partial sex identification results of E6.5 genital ridge tissues was showed in Figure 1B. In females there are two bands at approximately 434 bp and 580 bp positions, whereas in males there is one band at approximately 580 bp. A total of 307 developing embryonic eggs were collected in this experiment, of which 154 were males and 153 were females (Table 7). A total of 77 (E0, male 39, female 38), 50 (E3.5, male 23, female 27), 49 (E4.5, male 24, female 25), 48 (E5.5, male 24, female 24), 46 (E6.5, male 26, female 20) and 37 (E18.5, male 18, female 19) embryonic eggs were collected in each period. For each period, 18 female and 18 male samples were randomly selected, respectively, and each sequencing sample was a mix of six female or male samples with three replicates in each group. The quality of the extracted RNA was verified to meet the sequencing standards for subsequent experiments (RNA integrity number value>7, 28S/18S>0.7) (Supplements 1,2). High-quality raw transcriptomic data were obtained from 12 samples using the Illumina mRNA-seq sequence platform (Supplement 3), indicating that the sequencing quality was sufficient for subsequent expression analysis.

To assess the reliability of the experiments and the rationality of sample selection by examining the correlation of expression levels across samples, principal component analysis (PCA) was performed on the sequencing data (Figure 1C). The results revealed that the differences between the sexes were relatively small during the blastocyst stage, but gradually became more apparent as development progressed, particularly at E18.5d (Figure 1C). Although the differences between the sexes were minimal during the E3.5 d to E6.5 d developmental stages, the sample correlation analysis clearly demonstrated the existence of distinct and independent differences between male and female samples at different time points (Supplement 4). Furthermore, sex-associated genes in the sequencing data exhibited significant differences between the two sexes (Figure 1D; Supplement 5A; female-associated genes: HINTW, RSPO1, CYP19A1, FOXL2; male-associated genes: SPIN1Z, DMRT1, AMH, SOX9). These analytical results indicated that the sequencing data were accurate and reliable, making them suitable for subsequent analyses.

### Screening of key factors regulating chicken sex determination and differentiation

**Figure 2:**
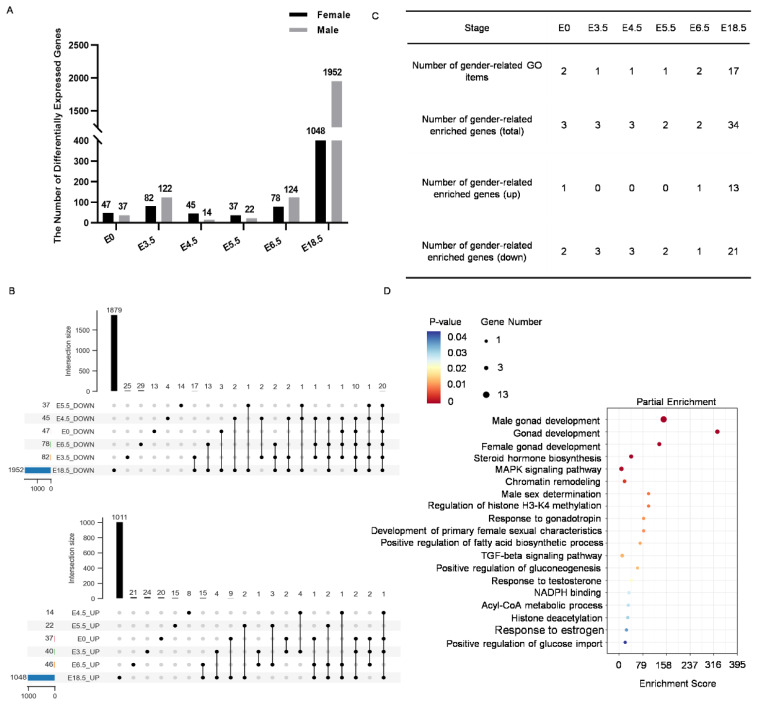
Screening and enrichment analysis of sex-related differentially expressed genes Based on RNA-seq data from E0 to E18.5 d. (A) Statistics on the number of differentially expressed genes between males and females at each stage. (B) UPSET analysis of differentially expressed genes in males and females from E0 to E18.5 d. Up, male; down, female. (C) Selected sex-related GO terms and the number and distribution of sex-related differentially expressed genes. (D) Bubble plot of enrichment analysis for selected sex-related differentially expressed genes. GO, Gene Ontology; TGF, transforming growth factor; NADPH, β-nicotinamide adenine dinucleotide 2′reduced.

To further analyze the DEGs between male and female chickens from E0 to E18.5 d, the number of DEGs at each developmental stage were counted. The results revealed that DEGs were already present at E0 d and gradually increased in number as development progressed (Figure 2A; Supplement 6). The DEGs obtained from the sequencing data of each time point were analyzed by a multi-sample upset analysis, and the results showed that there were continuously upregulated and downregulated DEGs from E0 throughout the process of sex determination and differentiation (Figure 2B). By PCA analysis, we found that there was a difference between males and females at E0 stage (Partial of Supplement 7). Through GO:0030154, cell differentiation screened 7 DEGs at E0 (shown in the volcano diagram) (Supplement 6A, E0 Volcano Plot). There were differences in gene expression between blastocysts of different sexes on E0 day, but it did not mean that the sex had been determined. The sex of chicken embryos may gradually change to both sexes at E0 stage. In addition, at E0, two genes (DMRT1 and HINTW) were identified as DEGs related to sex regulation. The sex-related gene expression profile showed that HINTW was indeed differentially expressed in female and male blastocysts from E0 (Supplement 5A). And the mRNA expression level of DMRT1, SOX9, HINTW, and FOXL2 in each period had been analyzed (Supplement 5B). HINTW and FOXL2 were differentially expressed between males and females at E0 and were significantly highly expressed in females (*p<0.0001). Although there was no significant difference between male and female at E0 stage, the expression of DMRT1 in males tended to be higher than that in females. These results further indicated that sex differentiation in chicken embryos began as early as the blastocyst stage.

To gain a deeper understanding of the functions and biological processes associated with sex determination and differentiation, a pool of gender-related genes was screened based on Gene Ontology (GO) terms in chickens from E0 to E18.5 d. A total of 17 GO terms were identified, involving 34 relevant genes (Figure 2C; Supplement 8). Notably, two gender-related GO terms emerged at E0 d, male sex determination (GO:0030238) and male genitalia development (GO:0030539), further suggesting that sex determination may commence at this early stage. By E18.5 d, the number of gender-related GO terms increased significantly, particularly those related to sex differentiation, gonadal development, and sex maintenance, indicating an increase in the expression of genes associated with these processes during later stages of embryonic development. Furthermore, enrichment analysis of the selected sex-related genes revealed that, in addition to terms related to gonadal development, sex determination, steroid hormone biosynthesis, and sex hormone response. Intriguingly, GO terms related to energy metabolism, including glucose response, positive regulation of glucose import, positive regulation of gluconeogenesis, acetyl-CoA metabolic process, and regulation of fatty acid biosynthesis, were also enriched (Figure 2D; Supplement 9). Moreover, epigenetic-related terms such as chromatin remodeling, histone H3 and H4 methylation, and histone deacetylation were among the enriched categories (Figure 2D; Supplement 9).

### Dynamic changes in energy metabolism during chicken sex determination and differentiation

**Figure 3:**
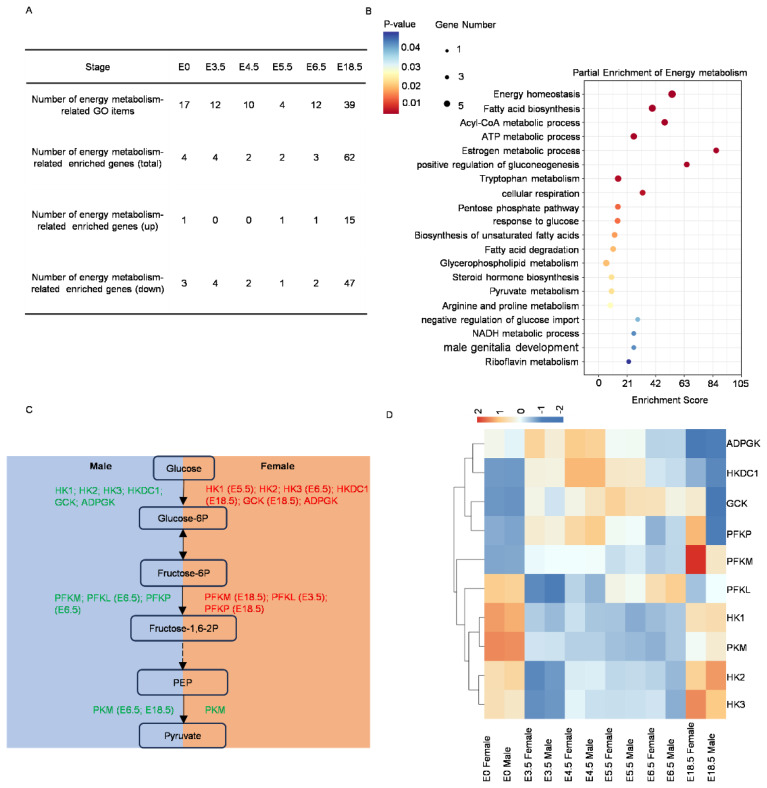
Screening and enrichment analysis of energy metabolism-related differentially expressed genes based on RNA-seq data from E0 to E18.5 d. (A) Selected GO terms related to energy metabolism and the number and distribution of corresponding differentially expressed genes. (B) Bubble plot of enrichment analysis for selected energy metabolism-related differentially expressed genes. (C) Schematic representation of high expression periods of glycolysis processes and key enzymes in male and female chicken embryos. Blue box: glycolysis process in male chicken embryos. Red box: Glycolysis process in female chicken embryos. (D) Heatmap analysis of key enzymes in the glycolysis process. The specific Gene IDs of the crucial enzymes in the glycolysis process, along with their mean FPKM values across distinct developmental stages, are already detailed in Table 4, which aligns with the presented heatmap. The default row normalization mode is z-score normalization. GO, Gene Ontology; ATP, adenosine triphosphate; NADPH, β-nicotinamide adenine dinucleotide 2′reduced; FPKM, fragments per kilobase of transcript per million mapped reads.

To investigate the impact of energy metabolism on sex determination and differentiation, 48 GO terms and 66 associated DEGs related to energy metabolism were screened during the E0 to E18.5 period (Figure 3A; Supplement 10). Notably, 17 GO terms related to energy metabolism were enriched at E0 d, and the number of enriched GO terms remained high during E3.5 to E6.5. Interestingly, a sharp increase in the number of GO terms related to energy metabolism was observed at E18.5 d. The pattern of changes in the number of these energy metabolism-related GO terms resembled the pattern observed for sex-related GO terms, with most of the energy metabolism-associated genes enriched in females (Figure 3A; Supplement 10). These results reasonably suggest that there exists a correlation between energy metabolism and sex determination and differentiation. The higher expression of energy metabolism-related genes in females during the later stages of embryonic development (E18.5 d) (Supplements 10, 10-1), coupled with the dramatic increase in the number of enriched genes, suggested that these genes might be involved in female sex differentiation and the maintenance of sexual characteristics.

To gain a deeper understanding of how energy metabolism-related genes participate in sex determination and differentiation, an enrichment analysis was conducted on the selected genes (Figure 3B; Supplement 11). The results demonstrated that these genes were not only enriched in terms related to the metabolism of the three major nutrients but also in processes such as estrogen metabolism, steroid biosynthesis, and male genital development. This result once again indirectly supports the involvement of energy metabolism in sex differentiation and maintenance of sexual characteristics during the early embryonic development of chickens.

Glycolysis is an indispensable part of energy metabolism, closely linked to other metabolic processes within the cell, and collectively maintains the normal life activities of the cell [30]. And the production of pyruvate is one of the key intermediates in glycolysis, and the process of pyruvate metabolism is highly enriched in the aforementioned analysis. Hexokinase, glucokinase, phosphofructokinase, and lactate dehydrogenase are crucial rate-limiting enzymes in glycolysis, playing significant regulatory roles in energy metabolism [31]. Analysis of the expression and distribution of key enzymes involved in glycolysis during the E0 to E18.5 period in both males and females, focusing on first-stage enzymes (HK1, HK2, HK3, HKDC1, GCK, ADPGK), second-stage enzymes (PFKM, PFKL, PFKP), and third-stage enzymes (PKM) (Figures 3C, 3D), revealed that the expression levels of these key glycolytic enzymes were generally higher in females than in males during the E0 to E5.5 d period. However, at E6.5 d and E18.5 d, the expression of the third-stage rate-limiting enzyme PKM was significantly higher in males than in females. Consistent with the transcriptome data, the relative expression levels of these key glycolytic enzymes were generally higher in females than in males during the E0 to E5.5 d period (GCK, E5.5; HK3, E0-E5.5; PFKM, E3.5 and E5.5), while it seemed to play a crucial role in male at E6.5 to E18.5 d (GCK, E6.5; PFKM, E6.5; PKM, E6.5) (Supplement 12A). The glycolytic process actively participated in early chicken embryonic sex determination and differentiation. Specifically, during the E0 to E5.5 d period, glycolysis appeared to play a more prominent role in female embryonic sex determination, differentiation, and maintenance, while it seemed to play a crucial role in male sex differentiation and maintenance at E6.5 to E18.5 d.

### DNA methylation and histone acetylation involved in chicken sex determination and differentiation

**Figure 4:**
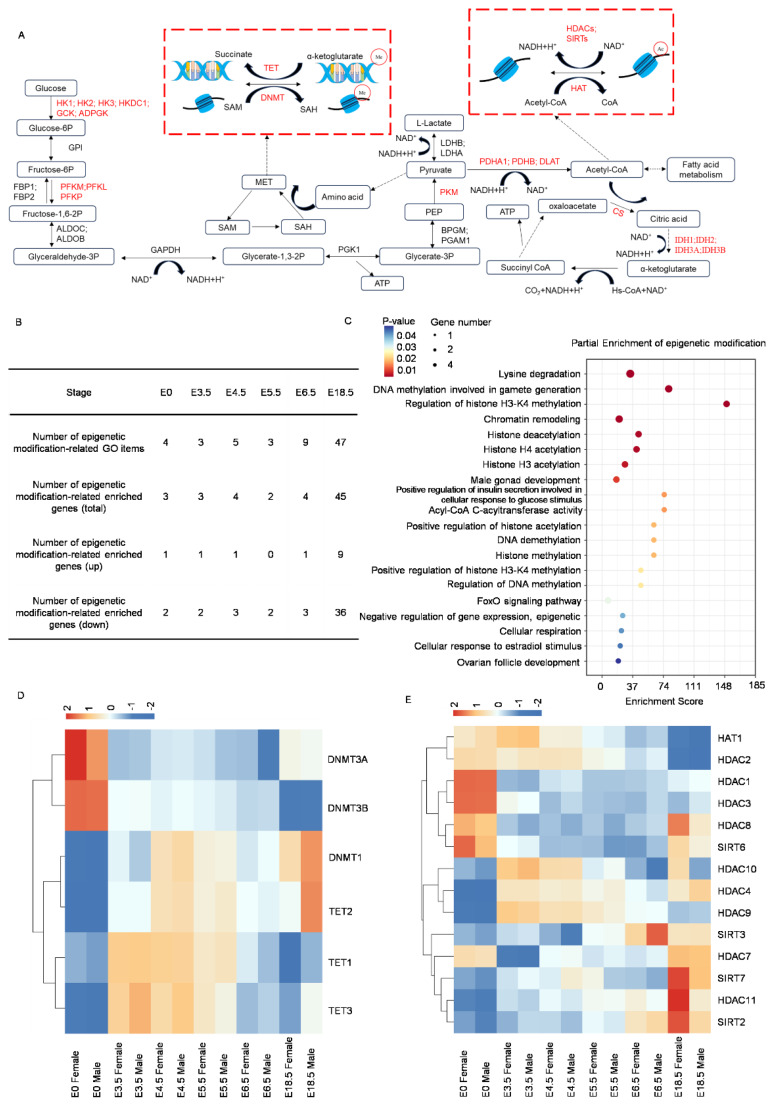
Screening and enrichment analysis of epigenetic modification-related differentially expressed genes based on RNA-seq data from E0 to E18.5 d. (A) Association between pyruvate metabolism and DNA/histone methylation and histone acetylation modifications. Key enzymes in these metabolic processes are shown in red font. The two red boxes represent DNA/histone methylation and histone acetylation processes, respectively. (B) Selected GO terms related to epigenetic modifications and the number and distribution of corresponding differentially expressed genes. (C) Bubble plot of enrichment analysis for selected epigenetic modification-related differentially expressed genes. (D) Heatmap analysis of key enzymes in the DNA methylation process. The comprehensive Gene ID of the pivotal enzymes involved in the DNA methylation process, along with their average FPKM values across various developmental stages, is already included in Table 5, which corresponds to the displayed heatmap. The default row normalization mode is z-score normalization. (E) Heatmap analysis of key enzymes in the histone acetylation process. The specific gene IDs of the crucial enzymes in the histone acetylation process, along with their mean FPKM values during distinct developmental stages, are detailed in Table 6, which aligns with the presented heatmap. The default row normalization mode is z-score normalization. GO, Gene Ontology; FPKM, fragments per kilobase of transcript per million mapped reads.

According to researches [32–34], energy metabolites provide substrates and raw materials for histone acetylation, DNA methylation, and histone methylation (Figure 4A). Given the role of energy metabolism in sex determination and differentiation, it is reasonable to speculate that epigenetic modifications also play a part in this process.

To analyze the dynamic changes of epigenetic modifications during chicken sex determination and differentiation, 48 GO terms and 49 related DEGs associated with epigenetics at E0 to E18.5 d (Figure 4B; Supplement 13) were screened. The number of enriched GO terms remained at a low level from E0 to E6.5 d, but increased sharply at E18.5 d. The pattern of these changes in the number of epigenetic modification-related GO terms resembled that of sex-related GO terms, with corresponding epigenetic modification-related genes predominantly enriched in females (Figure 4B, Number of epigenetic modification-related enriched genes (down), Supplement 13). These results suggested that epigenetic modifications might be involved in chicken sex determination and differentiation. Enrichment analysis of epigenetic modification-related genes (Figure 4C; Supplements 14C, 14D, 15, 16, 18) revealed the enrichment of DNA methylation, histone methylation, histone acetylation, chromatin remodeling, and other epigenetic-related terms. Additionally, processes related to the metabolism of three major nutrients, including pyruvate metabolism, fatty acid metabolism, and amino acid metabolism (tryptophan, valine, leucine, isoleucine, and lysine), were also enriched. These results corroborated our previous speculation regarding the relationship between the energy metabolism and epigenetic modifications. Notably, these epigenetic modification-related genes were also enriched in terms related to DNA methylation in gametogenesis, male gonadal development, cellular response to estrogens, and follicular development. This indirectly suggested that epigenetic modifications were involved in chicken sex determination and differentiation, as well as the maintenance of sexual characteristics during later stages of embryonic development.

To further analyze the impact of DNA or histone methylation on chicken embryo sex determination and differentiation, 29 GO terms and 24 related DEGs were found associated with DNA or histone methylation from E0 to E18.5 d (Supplements 14A, 18). The number of methylation-related GO terms was relatively low from E0 to E6.5 d but increased significantly at E18.5 d, suggesting that DNA/histone methylation may be involved in the regulation of late embryonic development. At E18.5 d, three GO terms related to DNA methylation (GO:1901536; GO:0043046; GO:0044030) were enriched (Supplement 20), and the corresponding genes were highly expressed in females (Supplement 20). Additionally, two GO terms related to DNA demethylation were enriched at E18.5 d, negative regulation of DNA demethylation (GO: 0010216, with GATA3 highly expressed in females) and DNA demethylation (GO:0080111, with TET2 highly expressed in males) (Supplement 20). This suggested that DNA methylation might play a more prominent role in female sex differentiation and maintenance. Moreover, a cluster heatmap was performed to analyze the expression of key enzymes involved in DNA methylation and demethylation at different stages (Figure 4D, Supplement 19). From E0 to E6.5 d, DNA methyltransferases (DNMT3A and DNMT3B) were mostly highly expressed in females (Supplement 19A). At E18.5d, DNA demethylases (TET2 and TET3) were mostly highly expressed in males (Supplement 19A). Consistent with the transcriptome data, the relative expression levels of these key enzymes of DNA methyltransferases (DNMT3A) were expressed at higher levels in female at E5.5 to E18.5 d, while the expression of DNA demethylases (TET2) was mostly higher expressed in males, especially at E4.5 (Supplement 19B). This suggested that the process of DNA methylation was more involved in the early embryonic development of female chicken embryos.

To further analyze the impact of histone acetylation on chicken sex determination and differentiation, 16 acetylation-related GO terms and 25 DEGs were mined associated with acetylation during E0 to E18.5 (Supplements 14B, 17). The expression of key enzymes involved in this process were also analyzed at various stages (Figure 4E). The number of GO terms related to histone acetylation was relatively low from E0 to E6.5 d but increased significantly at E18.5 d (Supplement 17). Additionally, the number of histone acetylation-related genes was higher in females than in males (Supplement 17-1), suggesting that histone acetylation may be involved in the regulation of late embryonic development. The key enzyme involved in histone acetylation, HAT1, was highly expressed at E0 d, with higher expression levels in males compared to females (Supplement 17-1, 21A). Subsequently, the expression of HAT1 gradually decreased, but overall, its expression was lower in females than in males. On the other hand, genes encoding histone deacetylases (HDAC1, HDAC2, HDAC3, HDAC8, and SIRT6) were expressed at higher levels at E0 d and showed a trend of higher expression in females compared to males (Supplement 17-1, 21). Other histone deacetylases (SIRT2, SIRT3, SIRT7, HDAC7, and HDAC11) were expressed at higher levels from E6.5 to E18.5 d, with SIRT2, SIRT7, and HDAC11 showing higher expression in females at E18.5 d (Supplement 17-1, 21A). Consistent with the transcriptome data, the relative expression levels of these key enzymes of histone deacetylases (SIRT7 and HDAC11) were expressed at higher levels in female at E18.5 d (Supplement 21B). Based on these findings, it could be inferred that histone acetylation might play a more prominent role in the early embryonic sex determination and differentiation of male chicken embryos, as well as the male maintenance of sexual characteristics.

### The role of glycolytic processes in sex differentiation and maintenance in chickens

**Figure 5:**
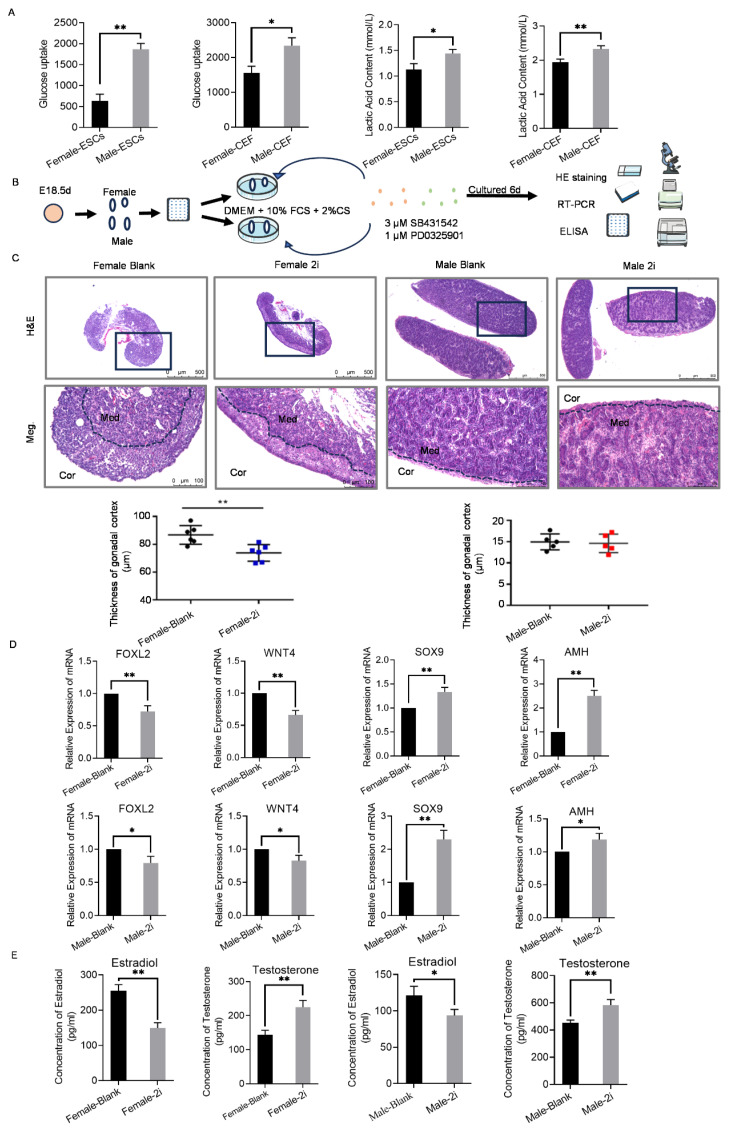
Effects of glycolysis activator treatment on E18.5 d gonads. (A) Glucose intake and lactate content were measured in male and female CEFs and ESCs. *p<0.05, significant difference; **p<0.01, extremely significant difference. (B) Schematic diagram of the strategy for culturing E18.5 d gonads *in vitro* using 2i as a glycolysis activator to detect gender-related gene expression, sex hormone production, and gonad tissue morphological changes. (C) HE staining of the testes and ovaries treated with 2i and statistical results of the cortical thickness of the testes and ovaries. Scale bars: 500 μm (upper), 100 μm (lower). (D) The expression of gender-related genes (FOXL2, WNT4, SOX9, and AMH) in testis and ovary treated with 2i were detected by qRT-PCR. (E) The levels of estradiol and testosterone in testes and ovaries treated with 2i were determined by ELISA. *p<0.05, significant difference; **p<0.01, extremely significant difference. ESC, embryonic stem cells; CEFs, chicken embryonic fibroblasts; DMEM, Dulbecco’s Modified Eagle Medium; FCS, fetal calf serum; HE, hematoxylin-eosin; RT-PCR, real-time polymerase chain reaction; ELISA, enzyme-linked immunosorbent assay; qRT-PCR, quantitative RT-PCR.

To further elucidate whether energy metabolism was associated with sex regulation in chickens, glucose and lactic acid levels were measured in ESC and CEFs. The glucose uptake and lactic acid content in male ESCs and CEFs was significantly higher than that in female, which once again confirmed the gender preference in the glycolysis process (Figure 5A). This trend was consistent with the sequencing data (Figure 3D; Supplement 12A), which showed that key enzyme (PKM, GeneID:396456) involved in the third phase of glycolysis was more expressed in males at E18.5 d.

To gain a deeper understanding of the regulatory role of glycolysis in sex differentiation and maintenance at E18.5 d, gonads were treated with a glycolysis activator, 2i, and examined changes in gonad morphology, sex-related gene expression, and sex hormone production (Figure 5B). After 2i treatment, the results revealed that the cortex/medulla ratio of ovarian tissue decreased significantly, with the cortex thinning and the medulla becoming denser and resembling a testicular-like structure. In contrast, no significant changes were observed in the testicular tissue (Figure 5C). The expression of male-related genes Sox9 and Amh was significantly upregulated in the ovaries treated with 2i, while the expression of female-related genes Foxl2 and Wnt4 was significantly downregulated (Figure 5D). Similarly, in the testes treated with 2i, Sox9 and Amh were upregulated, while Foxl2 and Wnt4 were downregulated (Figure 5D). Overall, the expression of male-related genes was upregulated in both testes and ovaries after 2i treatment, indicating a tendency towards male development. Additionally, estradiol levels were significantly reduced, while testosterone levels were significantly increased in both testes and ovaries after 2i treatment (Figure 5E).

These results suggested that the glycolysis activator could induce the female gonads at E18.5 d to develop male characteristics. Therefore, it could be speculated that the activation of glycolysis might promote male sex differentiation and maintain male sexual characteristics during the late stages of chicken embryonic development.

### The role of DNA methylation in chicken sex differentiation and maintenance

**Figure 6:**
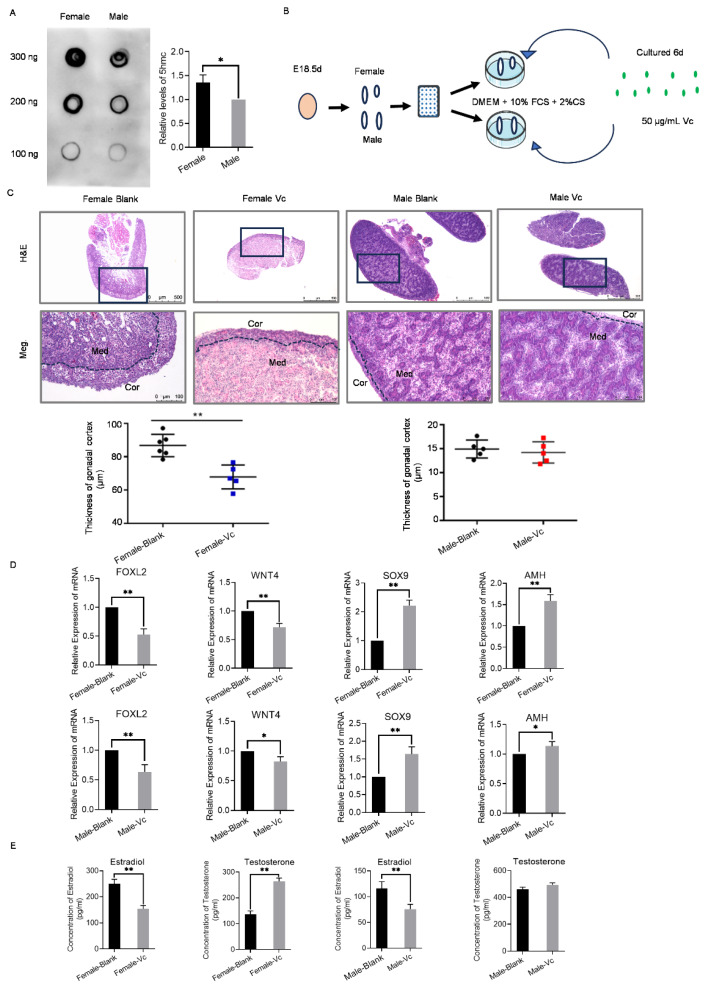
Effects of DNA methylation inhibitor treatment on E18.5 d gonads. (A) Dot Blot was used to detect the 5mc levels in female and male ESC. In the Dot Blot figure, the amount of DNA in each group was 300 ng, 200 ng, and 100 ng, respectively. *p<0.05, significant difference. The relative levels of 5hmc were statistically analyzed based on the grayscale analysis of Dot Blot. (B) Schematic diagram of the strategy for culturing E18.5 d gonads *in vitro* using the DNA methylation inhibitor Vc. (C) HE staining of the testes and ovaries treated with Vc and statistical results of the cortical thickness of the testes and ovaries. Scale bars: 500 μm (upper), 100 μm (lower). (D) The expression of gender-related genes (FOXL2, WNT4, SOX9, and AMH h) in testis and ovary treated with Vc were detected by qRT-PCR. (E) The levels of estradiol and testosterone in testes and ovaries treated with Vc were determined by ELISA. *p<0.05, significant difference; **p<0.01, extremely significant difference. FCS, fetal calf serum; HE, hematoxylin-eosin; RT-PCR, real-time polymerase chain reaction; ELISA, enzyme-linked immunosorbent assay; Vc, vitamin C; qRT-PCR, quantitative RT-PCR.

To further clarify the association between DNA methylation and sex regulation in chickens, Dot blot analysis was conducted using 5-methylcytosine antibodies on ESC genomic samples derived from both sexes. Notably, the DNA methylation level in female ESC was significantly higher than that in male ESC (Figure 6A; Supplement 22). This observation suggested that DNA methylation was actively involved in the regulation of sex determination and differentiation processes since the onset of embryonic development. This finding aligned with sequencing data indicating higher expression levels of DNA methyltransferases in females at E0 d (Figure 4D; Supplement 19A), further validating the profound involvement of DNA methylation in sex determination and differentiation of female chicken embryos during early embryogenesis.

To clarify the relationship between DNA methylation and sex differentiation and maintenance, gonads of E18.5 d were cultured with a DNA methylation inhibitor, Vc, and subsequent changes in gonadal morphology, sex-related gene expression and sex hormone production were examined (Figure 6B). The addition of Vc resulted in a notable thinning of the ovarian cortex and a gradual densification of the medulla, accompanied by the emergence of a curved, tubular morphology (Figure 6C). In contrast, no significant morphological changes were observed in the testes. Analysis of gene expression revealed a significant upregulation of male-related genes Sox9 and Amh in the ovaries treated with Vc, while the expression levels of female-related genes Foxl2 and Wnt4 were significantly downregulated (Figure 6D). Similarly, in the testes treated with Vc, the expression levels of Sox9 and Amh were upregulated, whereas the expression levels of Foxl2 and Wnt4 were downregulated (Figure 6D). Examination of sex hormone levels following Vc treatment revealed a significant increase in testosterone levels and a decrease in estradiol levels in ovaries (Figure 6E). And the estradiol levels in testes after Vc treatment were also significant decreased, but the testosterone levels in testes had no obvious change (Figure 6E). These results indicated that inhibitors of DNA methylation could induce masculinization of the gonads at E18.5 d. This finding corroborated sequencing data showing higher expression of DNA methyltransferases in females at E18.5 d. Collectively, these observations suggested that inhibition of the DNA methylation could promote male sex differentiation and maintain male sexual characteristics in chickens.

### The role of histone acetylation in chicken sex differentiation and maintenance

**Figure 7:**
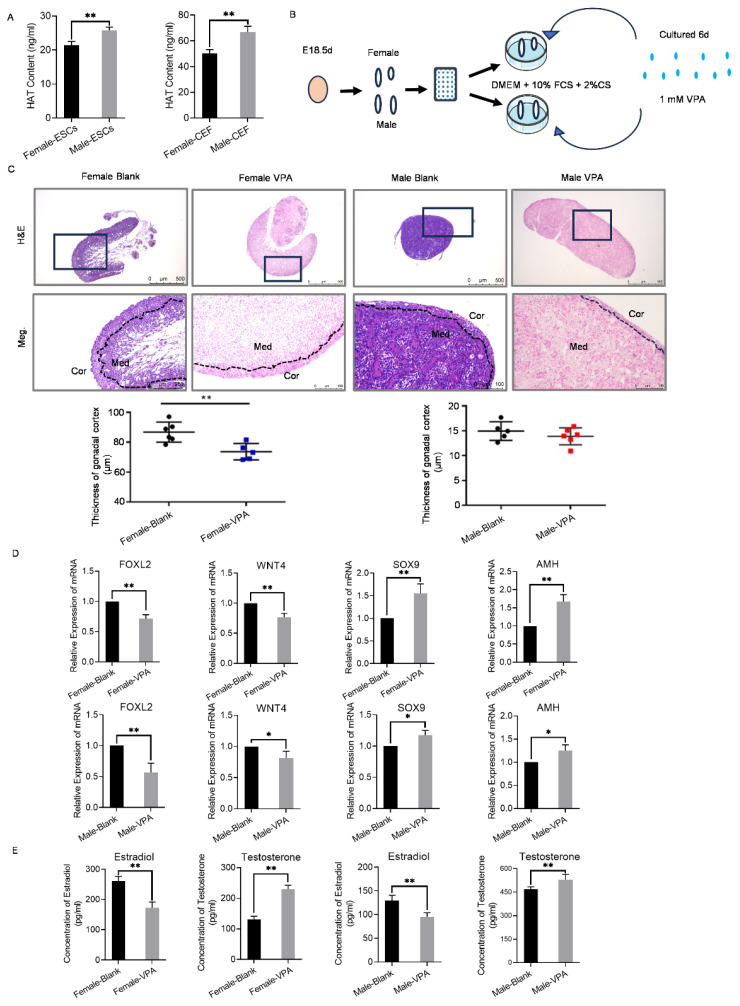
Effects of histone deacetylase inhibitor VPA treatment on male and female E18.5 d gonads. (A) HAT levels in ESC and CEF were detected by ELISA. **p<0.01, extremely significant difference. (B) Schematic diagram of the strategy for culturing E18.5 d gonads *in vitro* using the histone acetylation activator VPA. (C) HE staining of the testes and ovaries treated with VPA and statistical results of the cortical thickness of the testes and ovaries. Scale bars: 500 μm (upper), 100 μm (lower). D. The expression of gender-related genes (FOXL2, WNT4, SOX9, and AMH) in testis and ovary treated with VPA were detected by qRT-PCR. (E) The levels of estradiol and testosterone in testes and ovaries treated with VPA were determined by ELISA. *p<0.05, significant difference; **p<0.01, extremely significant difference. ESC, embryonic stem cells; CEFs, chicken embryonic fibroblasts; DMEM, Dulbecco’s Modified Eagle Medium; FCS, fetal calf serum; VPA, valproic acid; H&E, hematoxylin-eosin; HAT, histone acetylation enzyme; qRT-PCR, quantitative real-time polymerase chain reaction; ELISA, enzyme-linked immunosorbent assay.

To further elucidate whether histone acetylation is associated with sex regulation in chickens, the HAT ELISA kit was employed to assess changes in histone acetylation levels in both male and female ESCs and CEFs. Notably, the levels of histone acetyltransferase were significantly higher in male ESCs and CEFs compared to their female counterparts (Figure 7A). The HAT level assay results showed elevated HAT levels in male ESCs than female ESCs, which is consistent with transcriptome sequencing data (Supplement 21A; The FPKM value of HAT1 at E0 to E18.5) indicating gene expression differences between male and female ESCs at the E0 stage. Furthermore, among the DEGs at E0, we identified two GO terms related to histone deacetylation: HINTW (GO:0000118, histone deacetylase complex) and NIBPLL (GO:0045778, positive regulation of histone deacetylation) (Supplement 17). Therefore, based on our observations and transcriptome data, we reasonably hypothesize that histone acetylation is involved in regulating sex determination and differentiation in chicken embryos from the onset at E0. This finding aligns with sequencing data indicating higher expression levels of histone acetyltransferases in males at E0 d (Figure 4E; Supplements 14B, 21A), further validating the profound involvement of histone acetylation in sex determination and differentiation of male chicken embryos during early embryogenesis.

To gain a deeper understanding of the regulatory role of histone acetylation in sex differentiation and maintenance at E18.5 d, gonads at this stage were cultured with a histone deacetylation inhibitor, VPA, and subsequent changes in gonadal morphology, sex-related gene expression, and sex hormone production were examined (Figure 7B). Treatment with VPA resulted in a thinning of the ovarian cortex and the development of a denser medulla resembling the testicular medulla, while no significant morphological changes were observed in the testes (Figure 7C). Analysis of gene expression revealed a significant upregulation of male-related genes Sox9 and Amh in the ovaries treated with VPA, while the expression levels of female-related genes Foxl2 and Wnt4 were significantly downregulated (Figure 7D). Similarly, in the testes treated with VPA, the expression levels of Sox9 and Amh were upregulated, whereas the expression levels of Foxl2 and Wnt4 were downregulated (Figure 7D). Furthermore, the levels of testosterone were significantly increased in both the ovaries and testes, while estradiol levels decreased in both gonads compared to the control group treated with VPA (Figure 7E). These results indicated that inhibitors of histone deacetylation could induce masculinization of the gonads at E18.5 d. This finding corroborated sequencing data showing higher expression of histone deacetylation enzymes in females at E18.5 d. Collectively, these observations suggested that inhibiting the process of histone deacetylation could promote male sex differentiation and maintain male sexual characteristics in chickens.

## DISCUSSION

In this study, transcriptome sequencing technology was employed to comprehensively assess the expression of sex-related genes and the influencing factors involved in sex determination and differentiation during critical early embryonic development time points from E0 to E18.5 d in chickens. And the E18.5 d of chick embryo development represents a pivotal point in the process, characterized by the near-completion of organogenesis and the emergence of distinct sexual characteristics [35]. Functional studies have initially confirmed the crucial regulatory roles of glycolysis, DNA methylation, and histone acetylation in the early sex differentiation process in chick gonads at E18.5 d. This stage serves as a crucial window for studying sex differentiation. Our analysis highlights that the E18.5 stage offers unique advantages over earlier or later developmental stages, as it captures the critical period of sex differentiation without the confounding factors associated with earlier or more mature stages [36,37]. By focusing on this stage, we can gain deeper insights into the mechanisms underlying sex determination and sex-specific development, as evidenced by our transcriptome data showing a surge in DEGs between males and females.

Based on different determining factors, sex determination in vertebrates can be broadly classified into Genetic Sex Determination (GSD) and Environmental Sex Determination (ESD) [38–40]. Among vertebrates, mammals have their sex determined by genetic factors, possessing clear sex chromosomes, and the process of sex determination is controlled by these sex chromosomes [41]. Studies had shown that knocking out Foxl2 gene in female mice produced testis differentiation, resulting in the formation of testis tubules and spermatogonia [42], while Foxl2 loss of function expression is sufficient to cause an XX female-to-male sex reversal in the goat model [43]. And Sox9 triggers the male developmental pathway in chicken embryos and promoting testicular development [44]. In contrast, reptiles and fish generally belong to the environmentally determined type, characterized by the absence of definitive sex chromosomes. Their sex determination is influenced by external environmental factors such as temperature, light, and hormones [45,46]. In some aquatic organisms, temperature changes cause sex reversal by regulating downstream gene expression through epigenetic modifications [2]. The results in this research also confirmed that sex-related genes (such as Sox9 and Foxl2) are differentially expressed during chick embryo early sex determination and differentiation, and importantly, epigenetic modifications are also involved in the process of sexual differentiation. Knockout of Sry and Foxl2 induces sex reversal in mammals [5,43], but so far no such sex-determining genes have been found in chicken. Knockout of these genes can induce sex reversal [6], indicating that the sex determination mechanism in chickens is complex and requires the identification of additional factors to clarify its regulatory mechanisms. Research has revealed that the expression of the mammalian sex-determining gene Sry is not only controlled by specific transcription factors but also critically depends on epigenetic mechanisms [47,48]. Temperature-induced sex determination in fishes (such as Cichlid Fishes and Nile tilapia) [49, 50] and amphibians (frogs and turtles) [47,51] is even more reliant on epigenetic modifications. In our study, epigenetic modifications and energy metabolism were highly enriched as crucial factors in sex determination and differentiation during early embryonic development in chickens. This not only prompts us to consider the specific mode of sex determination in chicken embryos but also provides directions for further investigating the molecular mechanisms underlying chicken sex determination.

Epigenetic modifications regulate the expression of key genes or related genes involved in sex determination through DNA methylation and HATs, participating in the process of biological sex determination and differentiation. High methylation of the key gene Sry for sex determination leads to abnormal gene expression, which can silence genes such as DMRT1, SOX8, SOX9, NR5A1, and AMH involved in canine gonadal development, resulting in ovotestis formation [52]. Conversely, the absence of histone acetyltransferases p300 and CBP disrupts the histone acetylation of the Sry promoter in mice, leading to male-to-female sex reversal [53]. After Panobinostat (HDAC inhibitor) treated C57BL/6J mice ovary, the expression of SOX9 and DMRT1 increased while WNT4 decreased [54]. These researches suggested that the expression of sex-related genes was related to the degree of DNA methylation and positively related to the level of histone acetylation. Furthermore, a study showed that the m6A methylation level was positively correlated with gene expression abundance (SOX9 and CYP19A1) [55]. And the YTHDC2 (an N6-methyladenosine binding protein) could regulate the expression of sex-related genes, especially HEMGN and SOX9, in male mesonephros/gonad mingle cells, which was verified by *in vitro* experiments (E7 gonad mingle cells), suggesting a regulatory role of m6A methylation in chicken gonad differentiation [55]. Another research showed that both somatic and germline cell populations in morphologically feminized FemZZs (FemZZs for feminized ZZ gonads after exogenous estrogens treatment) maintain significant transcriptomic and epigenetic memories of genetic sex [56]. In our study, it is consistent with other species that inhibiting DNA methylation and histone deacetylases can promote the development of chicken embryos towards the male direction, and induce the downregulation of female sex-related genes Foxl2 and Wnt4, as well as the upregulation of male sex-related genes Amh and Sox9. In other words, it participates in the regulation of sex determination and differentiation of chicken embryos by regulating the expression of sex-related genes. Similar epigenetic modification patterns have also been observed in fish and amphibians with temperature-dependent sex determination [47]. High DNA methylation levels in the cyp19a1 promoter region induced by high temperatures lead to low cyp19a1 expression and the development of female larvae towards to the male direction [47,57]. This indicates that epigenetic modifications play an important role in sex determination and differentiation processes regardless of whether it is GSD or ESD sex determination.

Products or substrates of energy metabolism processes, such as glucose or pyruvate-lactate, affect the timing of porcine embryo cleavage and are more involved in male embryonic development [58]. Studies across various species, including humans [59], mice [33,60], bovines [61], Drosophila [62], and Gammarus [63], have demonstrated that glycolysis plays a pivotal energetic role in processes such as spermatogenesis, oogenesis, and gonadal tissue development [63,64]. For instance, in mice, glycolysis-related enzymes are abundant in primordial germ cells (PGCs), and spermatogonial stem cells exhibit a bioenergetics state that favors glycolytic activity [60]. The effects of the glycolytic inhibitor 2-deoxy-D-glucose and the electron transport chain inhibitor rotenone (a negative impact on PGC survival) on cultured mice male PGCs at E12.5 d separately revealed the importance of glycolysis and oxidative phosphorylation in PGC reprogramming and survival [33]. These results indicate that different energy metabolism processes play dominant roles at different stages in males and females. In our study. energy metabolism-related genes show expression patterns similar to sex-related genes, enriched mostly in females, suggesting a link between energy metabolism and sex determination/differentiation. Female embryos exhibit higher expression of these genes during late embryonic stages, indicating a role in female sex differentiation. However, males show higher expression of the glycolytic enzyme PKM at specific stages, suggesting specific metabolic features. Glycolysis is crucial for sex determination and differentiation in chicken embryos, with different roles in early female and later male development. A recent research demonstrated that metabolic processes such as glycolysis and oxidative phosphorylation have significant impacts on gonad development and sex differentiation in both males and females by influencing gene expression patterns, hormone activity (PPARG), and energy metabolism (PKM2) [65]. After treating the chicken gonads of E18.5 chick embryos with 2i, changes in the expression levels of Sox9, Amh, Foxl2, and Wnt4 further confirm that the activation of glycolysis can regulate the expression of sex-related genes, thereby influencing gonadal sex differentiation. The mechanism by which glycolytic activators influence male traits is unclear but may involve regulating sex-determining signaling pathways or altering cellular energy/redox states. Further research is needed to validate these hypotheses and understand the molecular basis of sex determination/differentiation in poultry.

Differences in energy metabolism levels exist between males and females [66], and these can participate in the regulation of sex determination and differentiation by altering intracellular epigenetic modifications [34].The dynamic shift in intracellular glycolysis and oxidative phosphorylation metabolic patterns not only provides raw materials for DNA and protein synthesis but also regulates the intracellular epigenetic modification level by altering the ratio of S-adenosylmethionine/S-adenosylhomocysteine to participate in gene expression regulation [32-34]. In mice male PGCs at E13.5, the authors observed that acetyl-CoA, a key substrate for histone acetylation, was undetectable, while NAD^+^, a cofactor for sirtuin-type histone deacetylases, exhibited a trend of abundance [33], hint at a potential mechanism where dynamic histone acetylation plays a pivotal role. This finding is closely related to the core content of our study, which focuses on the impact of histone acetylation on chicken sex determination and differentiation (Figure 4A). The lack of acetyl-CoA may limit the level of histone acetylation in mice male PGCs at E13.5. Histone acetylation is an important epigenetic modification typically associated with the activation of gene expression [67,68]. Therefore, the deficiency of acetyl-CoA may affect the expression patterns of specific genes in male PGCs, subsequently influencing sex determination and differentiation. In our study, enrichment analysis of genes related to energy metabolism reveals that these genes are significantly enriched in the GO terms associated with pyruvate metabolism and acetyl-CoA metabolism. These processes can provide raw materials for epigenetic modifications such as methylation and acetylation (Figure 4A). Thus, these results further demonstrate that energy metabolism is connected to epigenetic modification processes through substrates, products, and intermediates, and they collaborate with epigenetic modifications to participate in sex determination and differentiation regulation at different developmental stages.

## CONCLUSION

In summary, energy metabolism and epigenetic modifications play important roles in the sex determination and differentiation processes of early chicken embryonic development. These findings indicate that enhancing glycolysis, inhibiting DNA methylation, and promoting histone acetylation can regulate the expression of sex-related genes, promote the development of chicken embryos towards masculinity, and maintain male morphological characteristics. These discoveries have deepened our understanding of the potential regulatory mechanisms of chicken sex determination and differentiation, and provided theoretical support for further studying the regulatory network of chicken sex determination and differentiation.

## Figures and Tables

**Table 1:** Grouping of samples with description and naming

Group description	Group name
Female E0 blastoderm tissues	E0_female1; E0_female2; E0_female3
Male E0 blastoderm tissues	E0_male1; E0_male2; E0_male3
Female E3.5 genital ridge tissues	E3.5_female1; E3.5_female2; E3.5_female3
Male E3.5 genital ridge tissues	E3.5_male1; E3.5_male2; E3.5_male3
Male E4.5 genital ridge tissue	E4.5_male1; E4.5_male2; E4.5_male3
Female E5.5 genital ridge tissue	E5.5_female1; E5.5_female2; E5.5_female3
Male E5.5 genital ridge tissue	E5.5_male1; E5.5_male2; E5.5_male3
Female E6.5 genital ridge tissue	E6.5_female1; E6.5_female2; E6.5_female3
Male E6.5 genital ridge tissue	E6.5_male1; E6.5_male2; E6.5_male3
Female E18.5 ovary tissue	E18.5_female1; E18.5_female2; E18.5_female3
Male E18.5 testis tissue	E18.5_male1; E18.5_male2; E18.5_male3

**Table 2:** Sequences of the primers for qRT-PCR in this study

Gene	Gene ID	The sequence of primers (5′→3′)
*Foxl2*	NM_001012612	F: CTGATCGCCATGGCCATACG
		R: GGCGGATGCTGTTCTGCCA
*Sox9*	NM_204281	F: AGTACCCGCATCTGCACAA
		R: CCTCCTGCGTGGTTGGTA
*Amh*	NM_205030	F: GGATGGAGGTGCCCCTCTGT
		R: GCAGCATCACCCTCAGGTGG
*Wnt4*	NM_204783	F: TCTACGCCATCTCTTCAGCA
		R: AGGCAATGTTATCGGAGCAG
*β-actin*	NM_205518.2	F: CAGCCATCTTTCTTGGGTAT
		R: CTGTGATCTCCTTCTGCATCC

qRT-PCR, quantitative real-time polymerase chain reaction.

**Table 3 t3-ab-24-0679:** Heatmap corresponding to the Gene ID of sex-related genes and their mean FPKM values during different developmental stages

Gene	Gene ID	The average FPKM value of E0_female	The average FPKM value of E3_5_female	The average FPKM value of E4_5_female	The average FPKM value of E5_5_female	The average FPKM value of E6_5_female	The average FPKM value of E18_5_female	The average FPKM value of E0_male	The average FPKM value of E3_5_male	The average FPKM value of E4_5_male	The average FPKM value of E5_5_male	The average FPKM value of E6_5_male	The average FPKM value of E18_5_male
*DMRT1*	769693	0.111312667	0.649578	1.337125333	1.147684667	2.051791	11.68735	1.148534	1.449096667	2.048536667	2.16872	4.31161	20.6088
*AMH*	395887	1.286603333	0.042056967	0.289355667	8.012853333	29.71496	642.249	1.147046667	0.090940433	0.293858667	19.0807	220.8863333	1852.336667
*SOX9*	374148	0.680595333	2.724163333	3.40195	2.206946667	1.199057333	10.75475333	0.841243667	3.11003	4.2557	2.233993333	2.213046667	41.82643333
*SPIN1Z*	395344	13.99813333	22.30533333	24.67516667	28.85483333	20.1428	20.28546667	25.60943333	39.52263333	37.3355	34.80536667	35.3229	42.06066667
*CYP19A1*	414854	0	0.019444367	0.030087167	0.0530096	11.90929667	76.4887	0	0.022411953	0.00655295	0.315594133	1.188833333	0.014005167
*FOXL2*	503512	7.26752	0.182713333	0.363514733	0.443359667	2.541516667	64.4322	8.18005	0.202669667	0.2189235	0.563375	0.255245667	0.240556
*RSPO1*	419613	0	0.344454	0.769212333	0.869645	0.62447	0.877916	0	0.368442	0.788325667	0.670972333	0.622709333	0.0704204
*HINTW*	395423	278.2383333	144.0106667	135.286	89.135	107.6039667	37.06846667	44.0367	0.679892667	29.221647	37.67833333	12.79489	0.076467533

FPKM, fragments per kilobase of transcript per million mapped reads.

**Table 4 t4-ab-24-0679:** Heatmap corresponding to the Gene ID of key enzymes in glycolysis and their mean FPKM values during different developmental stages

Gene	Gene ID	The average FPKM value of E0_female	The average FPKM value of E0_male	The average FPKM value of E3.5_female	The average FPKM value of E3.5_male	The average FPKM value of E4.5_female	The average FPKM value of E4.5_male	The average FPKM value of E5.5_female	The average FPKM value of E5.5_male	The average FPKM value of E6.5_female	The average FPKM value of E6.5_male	The average FPKM value of E18.5_female	The average FPKM value of E18.5_male
*ADPGK*	415317	12.96486667	12.447	14.294	13.2762	14.5735	14.37743333	12.75273333	12.7778	11.89673333	11.94443333	9.983716667	10.69464
*GCK*	430370	0.906575667	0.882094667	1.539113333	1.26729	1.55273	1.773473333	2.032976667	1.832106667	1.79999	1.5581	1.68432	0.560969667
*HK1*	373889	40.7668	37.93323333	14.79813333	13.54876667	16.26533333	14.37416667	14.17263333	12.6314	13.60913333	14.42976667	26.44513333	27.47306667
*HK2*	374044	11.65490333	13.10666667	2.975096667	3.397596667	6.180946667	6.31337	5.378466667	5.489563333	5.19106	4.314046667	13.79296667	20.46463333
*HK3*	768421	1.193787667	1.090659667	0.312853	0.342374667	0.742530667	0.653987333	0.641334	0.646169333	0.664568	0.439001333	1.9357	1.492186667
*HKDC1*	423698	0.161231667	0.149995367	0.782394	0.759864667	1.495373333	1.496396667	0.898986	0.885266667	0.478533	0.433903	0.253466667	0.0919986
*PFKL*	769850	54.08526667	52.1547	15.2384	12.80993333	22.9048	19.47973333	34.08486667	31.33593333	45.0378	53.66863333	21.1422	29.31603333
*PFKM*	374064	0.198294	0.212241333	0.893429667	0.961469333	0.966032333	0.983766667	0.619964	0.747070333	0.484093333	0.552565333	6.9947	1.646006667
*PFKP*	428411	14.77036667	14.45786667	19.8906	19.3702	21.99006667	23.3175	18.41653333	18.1265	14.94186667	16.3667	24.77626667	12.48896667
*PKM*	396456	1222.976667	1170.276667	132.2336667	128.907	106.1133333	106.9293333	90.5228	84.00996667	75.1701	98.78733333	195.2726667	291.1023333

FPKM, fragments per kilobase of transcript per million mapped reads.

**Table 5 t5-ab-24-0679:** Heatmap corresponding to the Gene ID of key enzymes in DNA methylation and their mean FPKM values during different developmental stages

Gene	Gene ID	E0_female	E0_male	E3.5_female	E3.5_male	E4.5_female	E4.5_male	E5.5_female	E5.5_male	E6.5_female	E6.5_male	E18.5_female	E18.5_male
*DNMT1*	396011	5.67269333	5.30973333	8.95512333	8.04374667	10.9281333	11.4765	10.0908667	9.90132667	8.69871333	9.15331667	11.22389	14.0538667
*DNMT3A*	421991	38.3680333	31.6948	18.9974333	19.2655667	20.6549333	20.8820667	20.4009667	19.0884333	18.8693667	15.7405	22.9744667	22.5055667
*DNMT3B*	419287	319.348667	299.975	21.7659	22.552	18.6114	20.8152333	18.7632	16.5626	12.1664	12.9730333	2.03153	2.341
*TET1*	423690	6.54790333	6.21086333	11.3786667	11.4463	11.2034	11.0395667	9.61215667	9.96338667	7.80264333	6.80664667	4.1464	6.61642
*TET2*	422540	0.29360433	0.30030233	1.71100333	1.70077333	2.53301333	2.64798333	2.11121333	2.26554333	1.75676667	1.63480333	1.80375667	4.22259333
*TET3*	425829	3.96759333	3.65422333	6.84052333	7.43469667	6.60897	7.06543667	6.20625667	5.86043333	4.81527667	5.00810667	4.51821333	5.72574

FPKM, fragments per kilobase of transcript per million mapped reads.

**Table 6 t6-ab-24-0679:** Heatmap corresponding to the Gene ID of key enzymes in histone acetylation and their mean FPKM values during different developmental stages

Gene	Gene ID	E0_female	E0_male	E3.5_female	E3.5_male	E4.5_female	E4.5_male	E5.5_female	E5.5_male	E6.5_female	E6.5_male	E18.5_female	E18.5_male
*HAT1*	374037	61.0301	67.3657	79.0256	81.7673333	57.0384333	57.4978667	48.5721333	46.4608667	39.0002667	41.5044	30.4008667	22.8840333
*HDAC1*	373961	58.2503667	57.8256333	20.7151333	20.0532	24.3090333	25.0365667	21.7876	21.8562333	22.1377667	23.4905333	25.8323333	27.1770333
*HDAC2*	395635	110.046	108.577333	96.086	102.160067	103.6641	102.546333	89.8141	86.3615	73.7054333	79.1995333	45.4746667	39.7920667
*HDAC3*	395506	55.3196667	54.8798333	28.6600333	27.0500667	21.3466667	23.5584667	21.9365333	21.3857	21.0068	23.6128333	21.0942	24.9778333
*HDAC4*	374207	1.49456	1.36868667	5.68090333	5.61432	5.19928667	5.33353	4.66139	5.03423333	4.17385	3.6534	5.32724667	6.69927333
*HDAC7*	426885	5.70492667	5.53324667	2.56044	2.39541667	4.42065667	4.56399667	4.09228667	4.31426667	4.08801333	4.30055	6.71213667	6.62180333
*HDAC8*	422182	58.9333333	52.9684667	25.5291333	22.6871667	24.9148333	22.9434	25.6236333	22.6098667	25.9011333	27.5581	69.907	39.8420667
*HDAC9*	420599	0.254434	0.197043	2.09026333	1.9543	1.68301	1.71649333	1.51743	1.38664	1.17770333	1.15263	0.782178	0.83053033
*HDAC10*	17742	9.3048	8.66645333	14.1899667	14.8905667	13.1441667	13.0804667	10.5680667	11.2016667	9.2075	7.65276667	13.1632667	8.96132667
*HDAC11*	415978	3.42568	3.10150333	5.47519	5.0909	6.30882333	5.77334333	6.87978667	6.74240667	7.62458667	6.60135667	20.8913333	8.08336333
*SIRT2*	548628	33.3960333	31.1960667	34.7793	38.0497	37.7689667	35.7334667	40.9655	39.5501667	47.9707667	50.5401667	68.6706333	50.5551333
*SIRT3*	422988	9.62778333	9.32882333	10.1294067	10.2655833	9.54643333	8.66095	10.7125	10.79919	12.1536333	14.2743667	11.6378667	11.65
*SIRT6*	428332	30.1144333	25.7655	19.2597333	18.7866	16.6919667	16.803	16.6319667	15.3282333	15.3973667	16.8182	23.3322333	20.7643
*SIRT7*	103214197	6.79996667	6.53844333	7.50786	7.82698333	7.67546667	8.47933	8.19501667	7.21911	7.1802	6.88785	11.50468	9.75378667

FPKM, fragments per kilobase of transcript per million mapped reads.

**Table 7:** The Results of chicken embryo sex identification

The stage of sample	Male	Female	Total
E0 HH stage 1	39	38	77
E3.5 HH stage 21	23	27	50
E4.5 HH stage 26	24	25	49
E5.5 HH stage 28	24	24	48
E6.5 HH stage 30	26	20	46
E18.5 HH stage 43	18	19	37
Total	154	153	307

## SUPPLEMENTARY MATERIAL

**Supplement 1**. The quality control of RNA

**Supplement 2**. The quality information of RNA

**Supplement 3**. Transcriptional essence control and alignment results of next generation sequencing

**Supplement 4**. Correlation analysis of samples at E0-E18.5

**Supplement 5**. The FPKM value of gender-related genes (DMRT1, AMH, SOX9, RSPO1, SPIN1Z, CYP19A1, FOXL2, HINTW, DMRT1 and Amh) at E0-E18.5. B. The relative expression level of gender-related genes (DMRT1, SOX9, HINTW, and FOXL2) at E0-E18.5 were detected by qRT-PCR

**Supplement 6**. Statistical analysis of genes differentially expressed between male and female in each stage.

**Supplement 7**. The PCA analysis of samples at E0-E18.5

**Supplement 8**. Distribution statistics of GO items related to gender and corresponding related genes in different periods in male and female **Supplement 8-1**. The FPKM values of gender related differentially expressed genes during different developmental stages

**Supplement 9**. Gender related genes partial enrichment terms

**Supplement 10**. Distribution statistics of GO items related to energy metabolism and corresponding related genes in different periods in male and female **Supplement 10-1**. The FPKM values of energy metabolism related differentially expressed genes during different developmental stages

**Supplement 11**. Energy related genes partial enrichment terms

**Supplement 12**. A. The FPKM value of key enzymes in the glycolysis process (HK1, HK2, HK3, HKDC1, GCK, ADPGK, PFKM, PFKL, PFKP and PKM) at E0-E18.5. B. The relative expression level of gender-related genes (GCK, HK3, PFKM and PKM) at E0-E18.5 were detected by qRT-PCR

**Supplement 13**. Distribution statistics of GO items related to epigenetic modification and corresponding related genes in different periods in male and female **Supplement 13-1**. The FPKM values of epigenetic modification related differentially expressed genes during different developmental stages

**Supplement 14**. Enrichment analysis of DNA/histone methylation and histone acetylation related genes.

**Supplement 15**. Epigenetic modification related genes partial enrichment terms

**Supplement 16**. Acetylation related genes partial enrichment terms

**Supplement 17**.Distribution statistics of GO items related to histone acetylation and corresponding related genes in different periods in male and female **Supplement 17-1**. The FPKM values of histone acetylation related differentially expressed genes during different developmental stages

**Supplement 18**. Methylation related genes partial enrichment terms

**Supplement 19**. A. The FPKM value of key enzymes in the DNA methylation process (DNMT3A, DNMT3B, DNMT1, TET1, TET2 and TET3) at E0-E18.5. B. The relative expression level of gender-related genes (DNMT3A and TET2) at E0-E18.5 were detected by qRT-PCR

**Supplement 20**. Distribution statistics of GO items related to DNA/histone methylation and corresponding related genes in different periods in male and female **Supplement 20-1**. The FPKM values of methylation related differentially expressed genes during different developmental stages

**Supplement 21**. A. The FPKM value of key enzymes in histone acetylation (HAT1, HDAC2, HDAC1, HDAC3, HDAC8, SIRT6, HDAC10, HDAC4, HDAC9, SIRT3, HDAC7, SIRT7, HDAC11 and SIRT7) at E0-E18.5. B. The relative expression level of gender-related genes (HDAC11 and SIRT7) at E0-E18.5 were detected by qRT-PCR

**Supplement 22**. The original analysis process of Dot blot
