# Supplementary material for: Key events in the process of sex determination and differentiation in early chicken embryos
Source: Anim Biosci. 2025 Feb 27;38(6):1081–104. doi: 10.5713/ab.24.0679 (PMC12061580; doi:10.5713/ab.24.0679)
Supplement: Supplementary file 1 [file ab-24-0679-Supplementary-1.pdf]

Supplement 1. The quality control of RNA.

Sample	raw reads	raw bases	clean reads	clean bases	valid bases	Q30	GC
E0 Female1	50.81M	7.62G	49.74M	7.17G	94.05%	94.17%	50.89%
E0 Female2	48.37M	7.26G	47.30M	6.81G	93.79%	94.14%	51.96%
E0 Female3	50.95M	7.64G	49.79M	7.16G	93.72%	94.03%	51.87%
E0 Male1	50.79M	7.62G	49.61M	7.14G	93.75%	94.14%	52.00%
E0 Male2	49.42M	7.41G	48.34M	6.97G	93.97%	94.10%	51.41%
E0 Male3	50.02M	7.50G	48.89M	7.02G	93.61%	94.12%	51.28%
E18.5 Female1	49.90M	7.49G	48.70M	7.04G	94.01%	93.66%	48.67%
E18.5 Female2	48.87M	7.33G	47.55M	6.79G	92.64%	93.56%	49.18%
E18.5 Female3	50.36M	7.55G	49.01M	7.05G	93.32%	93.54%	49.60%
E18.5 Male1	49.85M	7.48G	48.85M	7.06G	94.46%	94.63%	49.15%
E18.5 Male2	47.28M	7.09G	46.35M	6.70G	94.40%	94.68%	49.31%
E18.5 Male3	48.04M	7.21G	46.91M	6.80G	94.29%	93.88%	49.39%
E3.5 Female1	47.13M	7.07G	46.21M	6.67G	94.42%	94.16%	47.57%
E3.5 Female2	48.71M	7.31G	47.77M	6.88G	94.18%	94.20%	47.44%
E3.5 Female3	47.86M	7.18G	46.93M	6.79G	94.55%	94.19%	47.66%
E3.5 Male1	51.52M	7.73G	50.48M	7.29G	94.36%	94.03%	47.66%
E3.5 Male2	51.24M	7.69G	50.21M	7.25G	94.37%	93.86%	47.68%
E3.5 Male3	50.48M	7.57G	49.49M	7.12G	93.96%	94.18%	47.89%
E4.5 Female1	51.39M	7.71G	50.46M	7.30G	94.66%	94.37%	48.30%
E4.5 Female2	40.99M	6.15G	40.16M	5.80G	94.37%	94.48%	48.19%
