# Supplementary material for: Key events in the process of sex determination and differentiation in early chicken embryos
Source: Anim Biosci. 2025 Feb 27;38(6):1081–104. doi: 10.5713/ab.24.0679 (PMC12061580; doi:10.5713/ab.24.0679)
Supplement: Supplementary file 2 [file ab-24-0679-Supplementary-2.pdf]

Supplement 2. The quality information of RNA.

Sample	Stage	Purity		Volum	Total	28S/18S	RIN value
		A260/280	A260/230	(μl)	(μg)		
Blastoderm	E0 male 1	1.91	1.47	35	22.18	1.2	8.8
	E0 male 2	1.84	1.42	35	16.82	1.3	9
	E0 male 3	1.84	1.59	35	13.77	1.2	8.6
Genital Ridge	E3.5 male 1	2.02	1.55	20	13.8	2.1	9.8
	E3.5 male 2	2.02	1.71	20	23.19	1.7	9.8
	E3.5 male 3	2.03	1.68	20	24.02	1.9	9.9
Genital Ridge	E4.5 male 1	1.97	1.68	25	26.35	1.7	10
	E4.5 male 2	1.97	1.72	25	26.39	1.4	10
	E4.5 male 3	1.98	1.74	25	31.86	1.7	10
Genital Ridge	E5.5 male 1	2	1.85	25	25.96	1.5	10
	E5.5 male 2	2.02	1.99	25	37.01	0.9	10
	E5.5 male 3	1.99	1.71	25	31.49	1.8	10
Genital Ridge	E6.5 male 1	2.03	1.99	45	82.14	2.2	9.8
	E6.5 male 2	2.01	1.7	45	89.35	2.2	9.8
	E6.5 male 3	2.02	2.04	45	81.89	2	10
Testis	E18.5 male 1	2.01	1.78	25	24.02	1.7	9.1
	E18.5 male 2	2	1.9	25	22.42	1.4	8.9
	E18.5 male 3	2.01	1.93	25	30.54	1.5	9.3
Blastoderm	E0 female 1	1.84	1.43	35	13.07	1.3	8.9
	E0 female 2	1.85	1.38	35	13.21	1.2	8.6
	E0 female 3	1.86	1.55	35	14.34	1.1	8.8
Genital Ridge	E3.5 female 1	2.03	1.67	20	23.86	1.9	10
	E3.5 female 2	1.93	1.54	20	13.81	1.9	9.9
	E3.5 female 3	2	1.86	20	22.11	1.7	9.9
Genital Ridge	E4.5 female 1	1.98	1.65	25	28.74	1.8	10
	E4.5 female 2	1.99	1.68	25	17.27	1.5	10
	E4.5 female 3	1.99	1.74	25	17.16	1.8	10
Genital Ridge	E5.5 female 1	2.02	2.03	25	28.62	1.7	9.9
	E5.5 female 2	2.02	1.95	25	32.36	1.7	10
	E5.5 female 3	1.98	1.76	25	31.38	1.5	10
Genital Ridge	E6.5 female 1	2.01	1.62	30	54.34	1.8	10
	E6.5 female 2	2.01	1.55	30	47.19	1.6	8.8
	E6.5 female 3	2.02	1.57	30	52.89	1.9	9.7
Ovary	E18.5 female 1	2	1.65	25	24.2	1.5	9
	E18.5 female 2	1.99	1.58	25	22.27	1	7.8
	E18.5 female 3	2	1.64	25	23.38	0.9	7.6
