# Supplementary material for: Key events in the process of sex determination and differentiation in early chicken embryos
Source: Anim Biosci. 2025 Feb 27;38(6):1081–104. doi: 10.5713/ab.24.0679 (PMC12061580; doi:10.5713/ab.24.0679)
Supplement: Supplementary file 3 [file ab-24-0679-Supplementary-3.pdf]

Supplement 3. Transcriptional essence control and alignment results of next generation sequencing.

Sample	Total reads	Total mapped reads	Multiple mapped	Uniquely mapped	Reads mapped in proper pairs
E0_Female1	49739248	46278932	1530483	44748449	43474558
		-93.04%	-3.08%	-89.97%	-87.40%
E0_Female2	47303788	43474697	1535262	41939435	40725530
		-91.91%	-3.25%	-88.66%	-86.09%
E0_Female3	49788380	45678214	1574623	44103591	42818562
		-91.74%	-3.16%	-88.58%	-86.00%
E0_Male1	49613154	45657513	1522582	44134931	42858576
		-92.03%	-3.07%	-88.96%	-86.39%
E0_Male2	48341038	44692489	1444656	43247833	41971388
		-92.45%	-2.99%	-89.46%	-86.82%
E0_Male3	48891198	45165361	1484798	43680563	42393508
		-92.38%	-3.04%	-89.34%	-86.71%
E18_5_Female1	48702416	45443492	1461630	43981862	42576660
		-93.31%	-3.00%	-90.31%	-87.42%
E18_5_Female2	47552840	44106115	1324058	42782057	41327376
		-92.75%	-2.78%	-89.97%	-86.91%
E18_5_Female3	49010472	45438327	1244255	44194072	42753750
		-92.71%	-2.54%	-90.17%	-87.23%
E18_5_Male1	48849800	45706062	1195164	44510898	43164208
		-93.56%	-2.45%	-91.12%	-88.36%
E18_5_Male2	46348706	43406706	1068756	42337950	41039898
		-93.65%	-2.31%	-91.35%	-88.55%
E18_5_Male3	46912846	43818556	1123787	42694769	41366988
		-93.40%	-2.40%	-91.01%	-88.18%
E3_5_Female1	46211212	43688289	866908	42821381	41485274
		-94.54%	-1.88%	-92.66%	-89.77%
E3_5_Female2	47771274	45275380	906681	44368699	43028878
		-94.78%	-1.90%	-92.88%	-90.07%
E3_5_Female3	46926504	44408560	901301	43507259	42213268
		-94.63%	-1.92%	-92.71%	-89.96%
E3_5_Male1	50475140	47616328	917817	46698511	45216850
		-94.34%	-1.82%	-92.52%	-89.58%
E3_5_Male2	50211588	47249687	919786	46329901	44863570
		-94.10%	-1.83%	-92.27%	-89.35%
E3_5_Male3	49490150	46842318	912507	45929811	44526078
		-94.65%	-1.84%	-92.81%	-89.97%
E4_5_Female1	50457644	47841094	1022479	46818615	45435136
		-94.81%	-2.03%	-92.79%	-90.05%
E4_5_Female2	40157844	38008284	803649	37204635	36087882
		-94.65%	-2.00%	-92.65%	-89.87%
E4_5_Female3	47553452	45055399	964883	44090516	42802034
		-94.75%	-2.03%	-92.72%	-90.01%
E4_5_Male1	46516402	44067585	899316	43168269	41875600
		-94.74%	-1.93%	-92.80%	-90.02%
E4_5_Male2	48753592	46251850	892955	45358895	43977306
		-94.87%	-1.83%	-93.04%	-90.20%
E4_5_Male3	47366462	44885873	910002	43975871	42653634
		-94.76%	-1.92%	-92.84%	-90.05%
E5_5_Female1	40354748	38242621	853148	37389473	36278386
		-94.77%	-2.11%	-92.65%	-89.90%
E5_5_Female2	39991868	37853985	811062	37042923	35913642
		-94.65%	-2.03%	-92.63%	-89.80%
E5_5_Female3	43248308	40954351	903532	40050819	38865538
		-94.70%	-2.09%	-92.61%	-89.87%
E5_5_Male1	46289394	43842426	963698	42878728	41602182
		-94.71%	-2.08%	-92.63%	-89.87%
E5_5_Male2	41471950	39251278	861975	38389303	37260184
		-94.65%	-2.08%	-92.57%	-89.84%
E5_5_Male3	41636186	39449022	875601	38573421	37415516
		-94.75%	-2.10%	-92.64%	-89.86%
E6_5_Female1	48609914	46094745	1102596	44992149	43698630
		-94.83%	-2.27%	-92.56%	-89.90%
E6_5_Female2	47449106	44916545	1046927	43869618	42543112
		-94.66%	-2.21%	-92.46%	-89.66%
E6_5_Female3	42169818	40004341	928786	39075555	37936122
		-94.86%	-2.20%	-92.66%	-89.96%
E6_5_Male1	48367540	45822449	1066606	44755843	43459612
		-94.74%	-2.21%	-92.53%	-89.85%
E6_5_Male2	47982972	45597217	1028372	44568845	43286644
		-95.03%	-2.14%	-92.88%	-90.21%
E6_5_Male3	47359660	44892304	1022522	43869782	42562250
		-94.79%	-2.16%	-92.63%	-89.87%
