# Supplementary figures and images for: Key events in the process of sex determination and differentiation in early chicken embryos
Source: Anim Biosci. 2025 Feb 27;38(6):1081–104. doi: 10.5713/ab.24.0679 (PMC12061580; doi:10.5713/ab.24.0679)

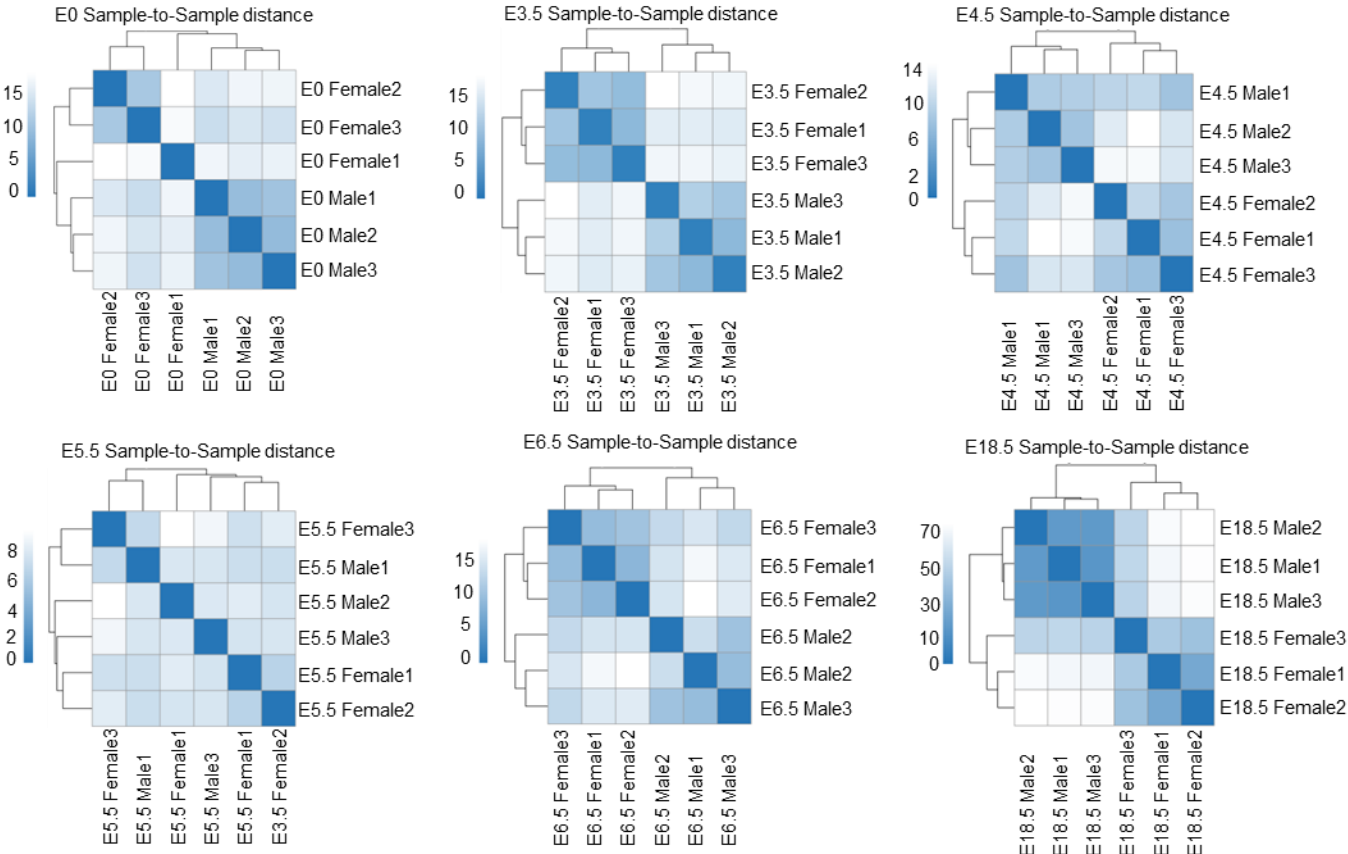

Supplement 4. Correlation analysis of samples at E0-E18.5.

Supplement: Supplementary file 4 [file ab-24-0679-Supplementary-4.pdf]

E0

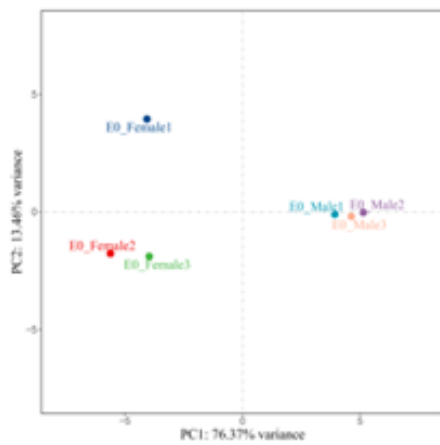

E3.5

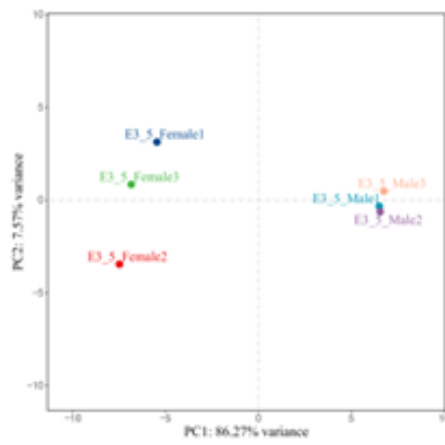

E4.5

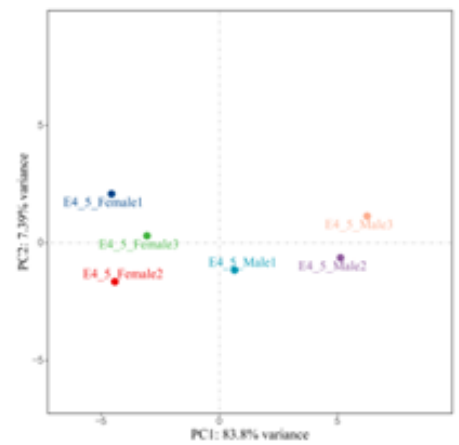

E5.5

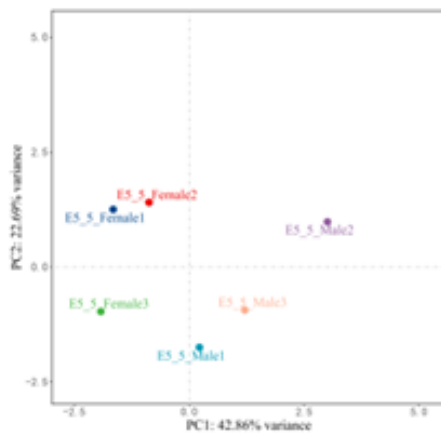

E6.5

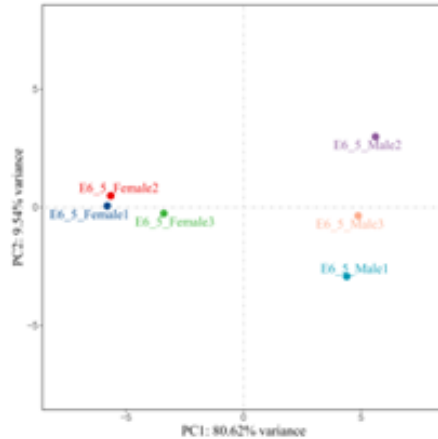

E18.5

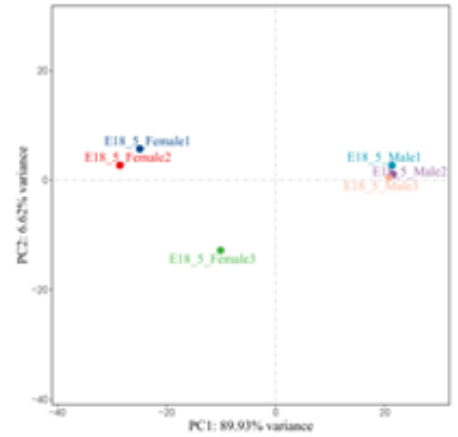

Supplement 7. The PCA analysis of samples at E0-E18.5.

Supplement: Supplementary file 7 [file ab-24-0679-Supplementary-7.pdf]

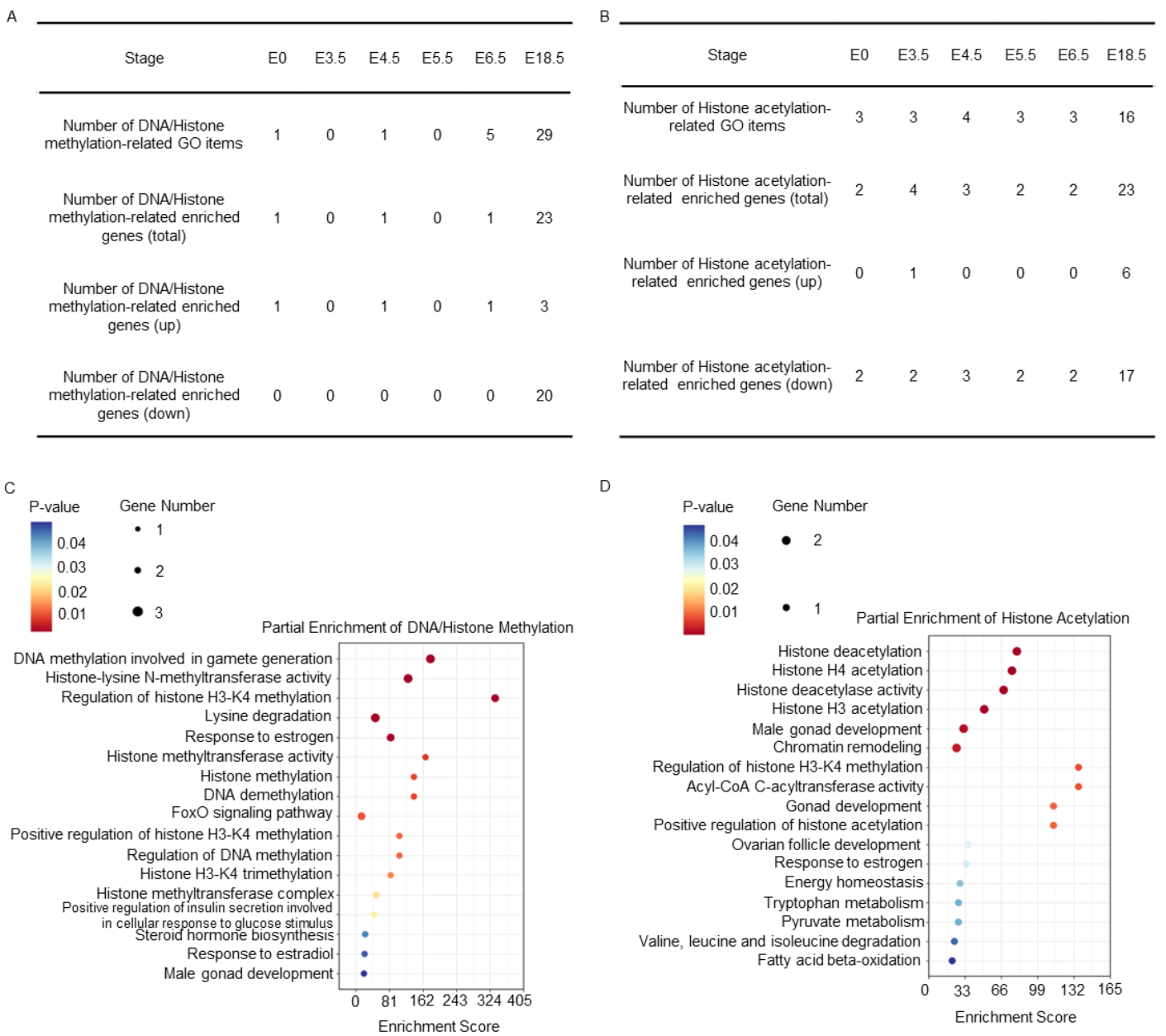

Supplement: Supplementary file 14 [file ab-24-0679-Supplementary-14.pdf]
