# Supplementary material for: Key events in the process of sex determination and differentiation in early chicken embryos
Source: Anim Biosci. 2025 Feb 27;38(6):1081–104. doi: 10.5713/ab.24.0679 (PMC12061580; doi:10.5713/ab.24.0679)
Supplement: Supplementary file 6 [file ab-24-0679-Supplementary-6.pdf]

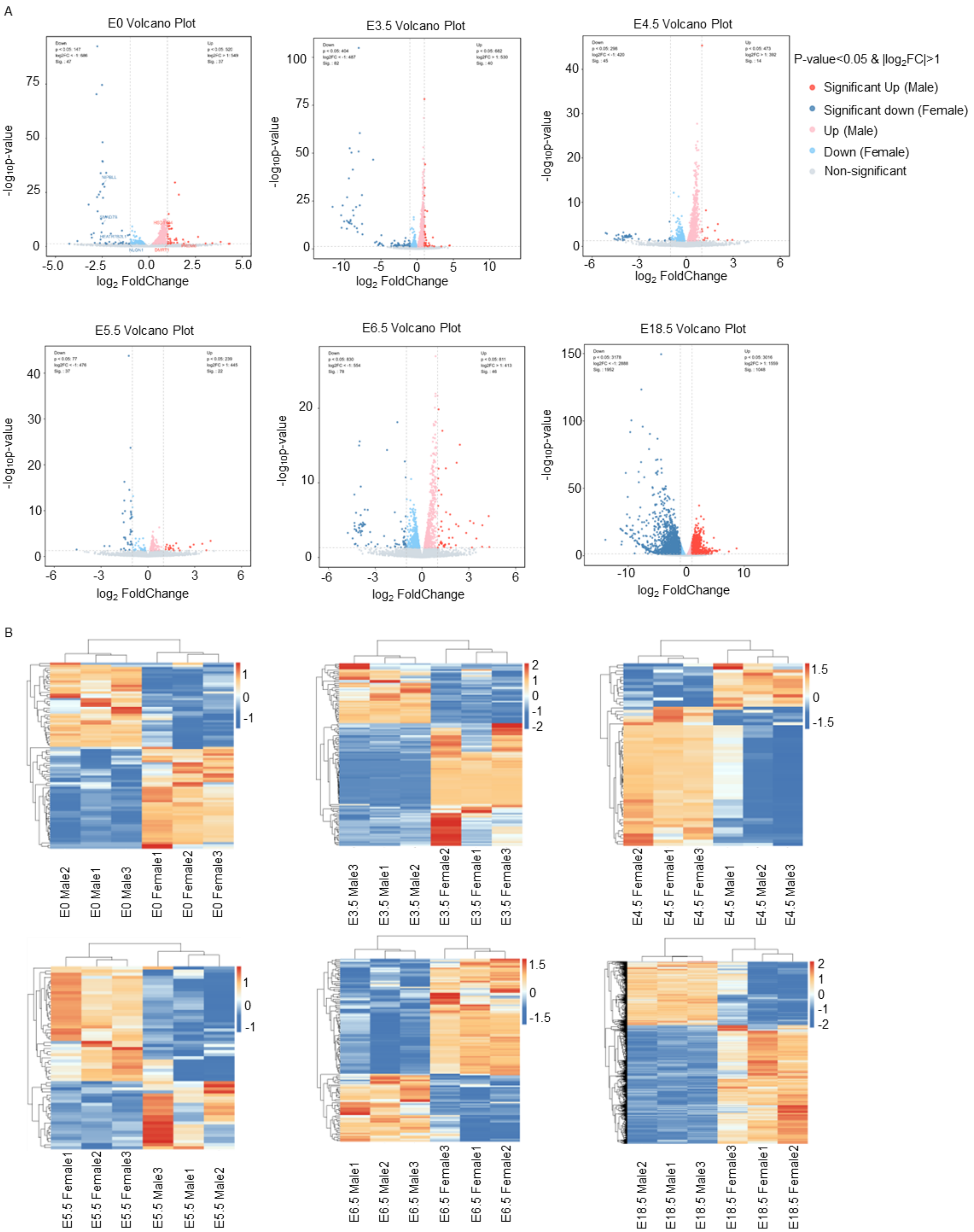

Supplement 6. Statistical analysis of genes differentially expressed between male and female in each stage. A. Volcano plot analysis of differentially expressed genes in each period. B. Cluster Heatmap analysis of differentially expressed genes in each period.
