# Supplementary material for: Key events in the process of sex determination and differentiation in early chicken embryos
Source: Anim Biosci. 2025 Feb 27;38(6):1081–104. doi: 10.5713/ab.24.0679 (PMC12061580; doi:10.5713/ab.24.0679)
Supplement: Supplementary file 8 [file ab-24-0679-Supplementary-8.pdf]

Supplement 8. Distribution statistics of GO items related to gender and corresponding related genes in different periods in male and female.

| id | term | category | ListHits | ListTotal | PopHits | PopTotal | pval | padj | Enrichment_score | Gene |
|---|---|---|---|---|---|---|---|---|---|---|
| E0 | | | | | | | | | | |
| GO:0030238 | male sex determination | biological_process | 1 | 69 | 5 | 14405 | 0.000224035 | 0.000844367 | 41.75362319 | DMRT1 |
| GO:0030539 | male genitalia development | biological_process | 2 | 69 | 13 | 14405 | 2.91E-05 | 0.000290676 | 32.11817168 | LOC100859467; LOC101752211 |
| E3.5 | | | | | | | | | | |
| GO:0030539 | male genitalia development | biological_process | 3 | 103 | 13 | 14405 | 1.68E-06 | 2.20E-05 | 32.27408514 | LOC100859467; LOC101752211; LOC112530485 |
| E4.5 | | | | | | | | | | |
| GO:0030539 | male genitalia development | biological_process | 3 | 46 | 13 | 14405 | 6.37E-08 | 2.18E-06 | 72.26588629 | LOC100859467; LOC101752211; LOC112530485 |
| E5.5 | | | | | | | | | | |
| GO:0030539 | male genitalia development | biological_process | 2 | 52 | 13 | 14405 | 1.24E-05 | 0.000184711 | 42.6183432 | LOC100859467; LOC101752211 |
| E6.5 | | | | | | | | | | |
| GO:0030238 | male sex determination | biological_process | 1 | 102 | 5 | 14405 | 0.000489648 | 0.001720696 | 28.24509804 | DMRT1 |
| GO:0008585 | female gonad development | biological_process | 1 | 102 | 10 | 14405 | 0.002153023 | 0.004950625 | 14.12254902 | CYP19A1 |
| E18.5 | | | | | | | | | | |
| GO:0046546 | development of primary male sexual characteristics | biological_process | 1 | 2499 | 1 | 14405 | 0 | 0 | 5.764305722 | LHFPL2 |
| GO:0007548 | sex differentiation | biological_process | 2 | 2499 | 3 | 14405 | 0.005215884 | 0.020955598 | 3.842870482 | CNTFR; HNF4A |
| GO:0019100 | male germ-line sex determination | biological_process | 1 | 2499 | 2 | 14405 | 0.030085852 | 0.070274844 | 2.882152861 | SOX9 |
| GO:0046661 | male sex differentiation | biological_process | 1 | 2499 | 3 | 14405 | 0.079825789 | 0.138156799 | 1.921435241 | SRD5A1 |
| GO:0030238 | male sex determination | biological_process | 1 | 2499 | 5 | 14405 | 0.209473809 | 0.288310387 | 1.152861144 | DMRT1 |
| GO:0046545 | development of primary female sexual characteristics | biological_process | 1 | 2499 | 5 | 14405 | 0.209473809 | 0.288310387 | 1.152861144 | LHFPL2 |
| GO:0032274 | gonadotropin secretion | biological_process | 1 | 2499 | 1 | 14405 | 0 | 0 | 5.764305722 | FAM129B |
| GO:2000020 | positive regulation of male gonad development | biological_process | 2 | 2499 | 2 | 14405 | 0 | 0 | 5.764305722 | SOX9; ZFPM2 |
| GO:2000195 | negative regulation of female gonad development | biological_process | 1 | 2499 | 1 | 14405 | 0 | 0 | 5.764305722 | ZFPM2 |
| GO:0008406 | gonad development | biological_process | 4 | 2499 | 6 | 14405 | 0.000804055 | 0.0041433 | 3.842870482 | IRX5; SALL1; TGFB2; WDR19 |
| GO:0008584 | male gonad development | biological_process | 16 | 2499 | 49 | 14405 | 0.002613414 | 0.012446715 | 1.882222277 | GATA3; HOXA10; INHBA; KIT; KITLG; KLHL32; LRRC6; NASP; PDGFA; REN; SOX9; SRD5A1; SRD5A2; TEX11; TGFB2; TLR5 |
| GO:0008585 | female gonad development | biological_process | 3 | 2499 | 10 | 14405 | 0.078859885 | 0.138156799 | 1.729291717 | CYP19A1; TGFB2; TOX2 |
| GO:0034698 | response to gonadotropin | biological_process | 1 | 2499 | 6 | 14405 | 0.279707589 | 0.362029371 | 0.96071762 | TOX2 |
| GO:0007621 | negative regulation of female receptivity | biological_process | 1 | 2499 | 1 | 14405 | 0 | 0 | 5.764305722 | PPP1R1B |
| GO:0030540 | female genitalia development | biological_process | 3 | 2499 | 4 | 14405 | 0.000903961 | 0.004493258 | 4.323229292 | SRD5A1; SRD5A2; TBX3 |
| GO:0030539 | male genitalia development | biological_process | 6 | 2499 | 13 | 14405 | 0.003031829 | 0.014193691 | 2.660448795 | LOC100859467; LOC101752211; LOC112530485; SRD5A1; SRD5A2; TBX3 |
| GO:0048808 | male genitalia morphogenesis | biological_process | 2 | 2499 | 4 | 14405 | 0.018151652 | 0.05976976 | 2.882152861 | LOC107054872; SYCP2 |

## Supplement 8-1. The FPKM values of gender related differentially expressed genes during different developmental stages.

| gene_id | baseMean | lfcSE | stat | foldChange | log2FoldChange | pval | padj | up_down | expression_Female1 | expression_Female2 | expression_Female3 | expression_Male1 | expression_Male2 | expression_Male3 | Dbxref |
|---|---|---|---|---|---|---|---|---|---|---|---|---|---|---|---|
| DMRT1 | 31.13675471 | 0.492037446 | 2.088300684 | 2.038520034 | 1.027522135 | 0.03677072 | 0.819506348 | Up | 0 | 0 | 0.333938 | 0.617072 | 1.4853 | 1.34323 | CGNC:7724,GeneID:769693 |
| LOC100859467 | 21.63971667 | 0.656390164 | -4.68646189 | 0.118573417 | -3.076147487 | 2.77969E-06 | 0.000223858 | Down | 0.90024 | 0.776454 | 0.592922 | 0.150647 | 0.0990492 | 0.0717503 | CGNC:63675,GeneID:100859467 |
| LOC101752211 | 10.75096995 | 0.81415208 | -1.995094861 | 0.324364844 | -1.624310632 | 0.046032535 | 0.951604856 | Down | 0.493656 | 0.380218 | 0.32349 | 0.238613 | 0.0325796 | 0.149825 | CGNC:71619,GeneID:101752211 |
| E3.5 | | | | | | | | | | | | | | | |
| LOC100859467 | 69.23562134 | 1.224341718 | -7.046978724 | 0.002527786 | -8.627910036 | 1.82844E-12 | 1.28314E-10 | Down | 2.51009 | 2.9463 | 2.69712 | 0 | 0.0225648 | 0 | CGNC:63675,GeneID:100859467 |
| LOC101752211 | 80.86613702 | 1.356704821 | -6.057351195 | 0.003358336 | -8.218037568 | 1.38381E-09 | 7.66235E-08 | Down | 3.44638 | 3.28404 | 2.95632 | 0 | 0.0542438 | 0 | CGNC:71619,GeneID:101752211 |
| LOC112530485 | 64.95817631 | 1.22589146 | -7.747510594 | 0.001383361 | -9.497607071 | 9.37115E-15 | 8.6604E-13 | Down | 2.94459 | 4.48785 | 3.19329 | 0 | 0 | 0 | GeneID:112530485 |
| E4.5 | | | | | | | | | | | | | | | |
| LOC100859467 | 111.8082542 | 1.848327019 | -2.137321091 | 0.064683057 | -3.950468321 | 0.032571885 | 0.60668243 | Down | 3.472 | 4.91874 | 3.73362 | 1.84657 | 0.431413 | 0 | CGNC:63675,GeneID:100859467 |
| LOC101752211 | 159.5274274 | 1.972366256 | -2.045199054 | 0.061049293 | -4.033881602 | 0.040835238 | 0.705199968 | Down | 4.39716 | 6.67432 | 6.65879 | 2.67344 | 0.476561 | 0 | CGNC:71619,GeneID:101752211 |
| LOC112530485 | 84.31159741 | 1.739656231 | -2.330725022 | 0.06017632 | -4.054660308 | 0.019767864 | 0.424591443 | Down | 5.07129 | 5.48925 | 5.09459 | 2.05211 | 0.453419 | 0 | GeneID:112530485 |
| E5.5 | | | | | | | | | | | | | | | |
| LOC100859467 | 79.77909604 | 0.316650919 | -3.446667038 | 0.469308896 | -1.091390287 | 0.000567548 | 0.156211799 | Down | 3.27447 | 2.01833 | 2.44003 | 1.21377 | 0.877162 | 1.5135 | CGNC:63675,GeneID:100859467 |
| LOC101752211 | 111.9714621 | 0.262350078 | -4.341092805 | 0.454110079 | -1.138886036 | 1.41776E-05 | 0.007804475 | Down | 4.10283 | 3.04009 | 3.43295 | 1.98408 | 1.12826 | 1.67148 | CGNC:71619,GeneID:101752211 |
| E6.5 | | | | | | | | | | | | | | | |
| DMRT1 | 182.7268271 | 0.210905534 | 5.896727021 | 2.367972555 | 1.24365236 | 3.70783E-09 | 4.93067E-07 | Up | 2.89296 | 2.52228 | 0.740133 | 3.80422 | 3.52857 | 5.60204 | CGNC:7724,GeneID:769693 |
| CYP19A1 | 725.6550602 | 1.040559006 | -3.889769806 | 0.060474259 | -4.047535001 | 0.000100339 | 0.004599515 | Down | 14.9347 | 13.0553 | 7.73789 | 2.5231 | 0.13684 | 0.90656 | CGNC:10026,GeneID:414854 |
| E18.5 | | | | | | | | | | | | | | | |
| LHFPL2 | 878.0718384 | 0.825403043 | 3.423711198 | 7.090766755 | 2.825941641 | 0.000617722 | 0.003389709 | Up | 0.717493 | 0.990131 | 5.8307 | 9.87007 | 11.9337 | 11.3272 | CGNC:3270,GeneID:416369 |
| CNTFR | 252.3446468 | 0.398910467 | -2.686065006 | 0.475824201 | -1.071499445 | 0.007229902 | 0.027262088 | Down | 5.20831 | 9.7687 | 2.34865 | 2.69172 | 1.83118 | 2.09577 | CGNC:20242,GeneID:395885 |
| SOX9 | 1143.930014 | 0.777871055 | 3.561479486 | 6.822837265 | 2.770371806 | 0.000368771 | 0.002158512 | Up | 2.9005 | 5.15846 | 24.2053 | 43.5844 | 41.5151 | 40.3798 | CGNC:49063,GeneID:374148 |
| SRD5A1 | 143.7874302 | 0.364775219 | -4.853327772 | 0.293132797 | -1.7703737 | 1.21407E-06 | 1.36109E-05 | Down | 3.50913 | 4.01568 | 2.00845 | 0.644563 | 1.12471 | 1.09059 | CGNC:56034,GeneID:770453 |
| DMRT1 | 1076.855431 | 0.242160805 | 4.829085558 | 2.249205138 | 1.169415248 | 1.37161E-06 | 1.51355E-05 | Up | 10.4743 | 8.32055 | 16.2672 | 21.8846 | 20.4706 | 19.4712 | CGNC:7724,GeneID:769693 |
| FAM129B | 951.4950517 | 0.271028485 | 4.611288252 | 2.378068825 | 1.24979047 | 4.00181E-06 | 3.89219E-05 | Up | 4.53447 | 5.11641 | 11.8389 | 13.4136 | 14.5511 | 14.5035 | CGNC:16803,GeneID:771307 |
| ZFPM2 | 434.695216 | 0.250527011 | 4.427763807 | 2.157371204 | 1.109274433 | 9.52151E-06 | 8.49365E-05 | Up | 2.2651 | 1.92488 | 3.14813 | 5.18003 | 5.16096 | 5.02222 | CGNC:12014,GeneID:420269 |
| IRX5 | 177.1774805 | 0.481308679 | -11.87661909 | 0.019020252 | -5.716319847 | 1.56573E-32 | 3.61348E-30 | Down | 2.08514 | 3.26874 | 1.21141 | 0.0569473 | 0.0334386 | 0.0256197 | CGNC:56270,GeneID:770775 |
| CYP19A1 | 4274.567835 | 1.974092552 | -7.055720111 | 6.41298E-05 | -13.92864452 | 1.71709E-12 | 5.49299E-11 | Down | 114.173 | 62.0392 | 53.2539 | 0 | 0 | 0.0420155 | CGNC:10026,GeneID:414854 |
| TOX2 | 248.1970091 | 0.270874693 | 3.96215271 | 2.104163631 | 1.073246901 | 7.4277E-05 | 0.000532538 | Up | 1.15571 | 1.74719 | 2.18208 | 3.22759 | 2.79267 | 4.1675 | CGNC:2248,GeneID:419156 |
| PPP1R1B | 216.7453662 | 0.286346567 | -8.095419256 | 0.200532015 | -2.31809551 | 5.70675E-16 | 2.94545E-14 | Down | 10.0083 | 20.0364 | 9.58236 | 2.2156 | 3.28612 | 2.08319 | CGNC:24263,GeneID:426639 |
| LOC107054872 | 100.2291506 | 0.3956075 | -5.476115703 | 0.222767018 | -2.166392443 | 4.34763E-08 | 6.46741E-07 | Down | 5.52393 | 2.60427 | 1.55756 | 0.671625 | 0.704057 | 0.602178 | CGNC:77840,GeneID:107054872 |
| GATA3 | 39.20737637 | 0.551651136 | -4.334348839 | 0.190643803 | -2.391048459 | 1.46192E-05 | 0.000124564 | Down | 0.74262 | 0.848998 | 0.76227 | 0.189228 | 0.118463 | 0.126845 | CGNC:5080,GeneID:419106 |
| HOXA10 | 185.9789053 | 0.330756696 | 3.331252529 | 2.14627372 | 1.101834079 | 0.000864561 | 0.004527387 | Up | 1.34437 | 0.810545 | 0.689909 | 1.9097 | 1.61058 | 2.29428 | CGNC:16063,GeneID:776143 |
| INHBA | 192.2401884 | 0.526693521 | 2.72629422 | 2.705549248 | 1.435921502 | 0.006404988 | 0.024677457 | Up | 0.359976 | 0.478444 | 1.97717 | 2.30368 | 1.84421 | 1.42329 | CGNC:9365,GeneID:396361 |
| KIT | 331.1858248 | 0.248886079 | 7.546781552 | 3.676387577 | 1.878288868 | 4.46146E-14 | 1.78846E-12 | Up | 1.33271 | 0.796454 | 1.32245 | 4.88062 | 3.67613 | 3.8259 | CGNC:10414,GeneID:378783 |
| KITLG | 319.0265572 | 0.241975733 | 4.350685002 | 2.074495017 | 1.052760192 | 1.35713E-05 | 0.000116868 | Up | 5.25688 | 6.53432 | 4.76249 | 12.966 | 10.3853 | 13.0606 | CGNC:8510,GeneID:396028 |
| KLHL32 | 50.7964689 | 0.529465186 | -2.748450161 | 0.36470233 | -1.455208677 | 0.005987774 | 0.02331465 | Down | 0.673489 | 0.674787 | 0.456825 | 0.180674 | 0.178838 | 0.363421 | CGNC:11569,GeneID:421801 |
| LRRC6 | 109.8144374 | 0.376737713 | -3.153286366 | 0.438922492 | -1.187961895 | 0.001614433 | 0.007704926 | Down | 2.86503 | 3.53456 | 2.11655 | 1.13218 | 1.05535 | 1.22986 | CGNC:12145,GeneID:420324 |
| NASP | 7242.013346 | 0.256593684 | 4.019211587 | 2.043871236 | 1.03130431 | 5.83932E-05 | 0.000428403 | Up | 32.6681 | 33.4875 | 64.6748 | 66.0193 | 83.3416 | 63.774 | CGNC:7802,GeneID:424600 |
| PDGFA | 665.8715264 | 0.340903475 | 5.049866304 | 3.297829865 | 1.721516972 | 4.42119E-07 | 5.47313E-06 | Up | 6.87995 | 7.11425 | 19.3984 | 33.5479 | 34.6347 | 29.5734 | CGNC:49079,GeneID:374196 |
| REN | 7.446576259 | 1.442071806 | -2.762254874 | 0.063224614 | -3.983369874 | 0.005740365 | 0.022541467 | Down | 0.0717905 | 0.10639 | 0.103828 | 0.013925 | 0 | 0.0139912 | CGNC:54151,GeneID:431167 |
| SRD5A2 | 203.5654446 | 0.265937364 | -7.482525021 | 0.251759307 | -1.989882982 | 7.2908E-14 | 2.83132E-12 | Down | 4.69931 | 4.81905 | 3.93567 | 1.3786 | 1.27683 | 1.12393 | CGNC:8069,GeneID:772291 |
| TEX11 | 979.5343573 | 0.199378494 | -5.05222892 | 0.497474405 | -1.007305794 | 4.36684E-07 | 5.41047E-06 | Down | 11.4803 | 10.5864 | 8.56132 | 3.35383 | 3.2272 | 4.08786 | CGNC:4306,GeneID:422215 |
| TGFB2 | 1174.472526 | 0.219218297 | -6.887095277 | 0.351165423 | -1.509777298 | 5.69431E-12 | 1.69726E-10 | Down | 18.3281 | 23.3689 | 14.0477 | 5.90143 | 6.39957 | 7.16474 | CGNC:51471,GeneID:421352 |
| TLR5 | 142.7151157 | 0.303588751 | -3.592912329 | 0.469511446 | -1.090767767 | 0.000327003 | 0.001942179 | Down | 0.400016 | 0.445271 | 0.331714 | 0.20722 | 0.159054 | 0.174612 | CGNC:7140,GeneID:554217 |
| LOC100859467 | 75.20188969 | 1.258338503 | -7.670515186 | 0.001242874 | -9.652104595 | 1.71307E-14 | 7.32133E-13 | Down | 3.96465 | 3.22535 | 2.51572 | 0 | 0 | 0 | CGNC:63675,GeneID:100859467 |
| LOC101752211 | 79.21596243 | 1.263150155 | -6.926547148 | 0.002323847 | -8.749269106 | 4.31236E-12 | 1.30706E-10 | Down | 3.89572 | 3.41484 | 2.14842 | 0.0265047 | 0 | 0 | CGNC:71619,GeneID:101752211 |
| LOC112530485 | 36.89925327 | 1.32864373 | -5.734313956 | 0.005087384 | -7.618860281 | 9.79078E-09 | 1.68445E-07 | Down | 2.98565 | 3.00316 | 1.56773 | 0 | 0 | 0.0454396 | GeneID:112530485 |
| TBX3 | 2274.826476 | 0.636111005 | 2.801890677 | 3.439773321 | 1.782313495 | 0.005080409 | 0.020406266 | Up | 9.7066 | 9.21132 | 36.4981 | 44.6781 | 51.3748 | 40.2918 | CGNC:49082,GeneID:374202 |
| HNF4A | 23.8213962 | 0.808920555 | -2.353879394 | 0.267182713 | -1.904101426 | 0.018578638 | 0.059126607 | Down | 0.0993508 | 0.403626 | 0.885643 | 0.0837417 | 0.0384096 | 0.122061 | CGNC:50941,GeneID:419198 |
| WDR19 | 653.095199 | 0.244183696 | -4.136787036 | 0.496499459 | -1.010135948 | 3.52203E-05 | 0.000272784 | Down | 9.05763 | 5.69867 | 4.3957 | 3.63758 | 2.70448 | 3.44264 | CGNC:10496,GeneID:430829 |
| SALL1 | 2343.426352 | 0.223134809 | -9.465996479 | 0.231295112 | -2.112193314 | 2.90775E-21 | 2.62429E-19 | Down | 39.0761 | 38.8265 | 24.7806 | 7.55559 | 6.92216 | 8.59143 | CGNC:49323,GeneID:395446 |
| SYCP2 | 804.4446738 | 0.254171641 | -10.78419057 | 0.149577448 | -2.741035416 | 4.08827E-27 | 6.5392E-25 | Down | 12.5879 | 8.86341 | 5.95987 | 1.31365 | 0.869952 | 1.25925 | CGNC:3623,GeneID:419219 |
