# Supplementary material for: Key events in the process of sex determination and differentiation in early chicken embryos
Source: Anim Biosci. 2025 Feb 27;38(6):1081–104. doi: 10.5713/ab.24.0679 (PMC12061580; doi:10.5713/ab.24.0679)
Supplement: Supplementary file 9 [file ab-24-0679-Supplementary-9.pdf]

Supplement 9. Gender related genes partial enrichment terms.

Term_ID	Term_description	ListHits	FoldEnrichment	p-value	q-value	geneID
gga00140	Steroid hormone biosynthesis	3	40.43513514	4.35E-05		CYP19A1;SRD5A1;SRD5A2
gga04010	MAPK signaling pathway	4	8.01124498	0.001003505		KIT;KITLG;PDGFA;TGFB2
gga04350	TGF-beta signaling pathway	2	10.38958333	0.014924072		INHBA;TGFB2
GO:0051569	regulation of histone H3-K4 methylation	1	98.53793103	0.010108673	0.04052036	GATA3
GO:0016575	histone deacetylation	1	28.98174442	0.033968577	0.061324951	SALL1
GO:0045722	positive regulation of gluconeogenesis	1	61.5862069	0.016126444	0.045675496	HNF4A
GO:0046326	positive regulation of glucose import	1	20.52873563	0.047629404	0.072673251	LOC100859467
GO:0045723	positive regulation of fatty acid biosynthetic process	1	70.38423645	0.014124455	0.043280035	HNF4A
GO:0006637	acyl-CoA metabolic process	1	30.79310345	0.032001653	0.059593629	HNF4A
GO:0043627	response to estrogen	1	24.63448276	0.039846226	0.065899528	GATA3
GO:0034698	response to gonadotropin	1	82.11494253	0.012118535	0.041839904	TOX2
GO:0033574	response to testosterone	1	41.05747126	0.024095215	0.052041264	NASP
GO:0030238	male sex determination	1	98.53793103	0.010108673	0.04052036	DMRT1
GO:0046545	development of primary female sexual characteristics	1	82.11494253	0.012118535	0.041839904	LHFPL2
GO:0008584	male gonad development	13	148.9526864	1.46E-26	6.89E-24	SOX9;GATA3;HOXA10;INHBA;KIT;KITLG;LRRC6;NASP;PDGFA;REN;SRD5A2;TEX11;TGFB2
GO:0008406	gonad development	4	328.4597701	2.05E-10	4.84E-08	IRX5;TGFB2;WDR19;SALL1
GO:0008585	female gonad development	3	134.369906	1.23E-06	8.29E-05	CYP19A1;TOX2;TGFB2
GO:0070402	NADPH binding	1	32.84597701	0.030030861	0.058215563	SRD5A1
GO:0006338	chromatin remodeling	2	18.24776501	0.005331976	0.030385838	SOX9;GATA3
