# Supplementary material for: Key events in the process of sex determination and differentiation in early chicken embryos
Source: Anim Biosci. 2025 Feb 27;38(6):1081–104. doi: 10.5713/ab.24.0679 (PMC12061580; doi:10.5713/ab.24.0679)
Supplement: Supplementary file 10 [file ab-24-0679-Supplementary-10.pdf]

Supplement 10. Distribution statistics of GO items related to energy metabolism and corresponding related genes in different periods in male and female.

id	term	category	ListHits	ListTotal	PopHits	PopTotal	pval	padj	Enrichment_score	Gene
E0										
GO:0005753	mitochondrial proton-transporting ATP synthase complex	cellular_component	1	69	14	14405	0.00198265	0.003918511	14.91200828	ATP5A1W
GO:1903715	regulation of aerobic respiration	biological_process	1	69	2	14405	2.26E-05	0.000240489	104.384058	TERAL2
GO:0006734	NADH metabolic process	biological_process	1	69	7	14405	0.000467564	0.001338751	29.82401656	TERAL2
GO:1903862	positive regulation of oxidative phosphorylation	biological_process	1	69	2	14405	2.26E-05	0.000240489	104.384058	TERAL2
GO:0033540	fatty acid beta-oxidation using acyl-CoA oxidase	biological_process	1	69	9	14405	0.000796583	0.001954985	23.19645733	HSD17B4
GO:0006635	fatty acid beta-oxidation	biological_process	1	69	27	14405	0.007346544	0.010962549	7.732152442	HSD17B4
GO:0036111	very long-chain fatty-acyl-CoA metabolic process	biological_process	1	69	1	14405	0	0	208.7681159	HSD17B4
GO:0036112	medium-chain fatty-acyl-CoA metabolic process	biological_process	1	69	1	14405	0	0	208.7681159	HSD17B4
GO:0004321	fatty-acyl-CoA synthase activity	molecular_function	1	69	2	14405	2.26E-05	0.000240489	104.384058	ACSM4L
GO:0006637	acyl-CoA metabolic process	biological_process	1	69	15	14405	0.002280607	0.004390824	13.9178744	ACSM4L
GO:1904949	ATPase complex	cellular_component	1	69	2	14405	2.26E-05	0.000240489	104.384058	TERAL2
GO:0045261	proton-transporting ATP synthase complex, catalytic core F(1)	cellular_component	1	69	5	14405	0.000224035	0.000844367	41.75362319	ATP5A1W
GO:2001171	positive regulation of ATP biosynthetic process	biological_process	1	69	5	14405	0.000224035	0.000844367	41.75362319	TERAL2
GO:0046034	ATP metabolic process	biological_process	1	69	27	14405	0.007346544	0.010962549	7.732152442	TERAL2
GO:0016887	ATPase activity	molecular_function	1	69	91	14405	0.070533153	0.081337715	2.29415512	TERAL2
GO:0044594	17-beta-hydroxysteroid dehydrogenase (NAD+) activity	molecular_function	1	69	1	14405	0	0	208.7681159	HSD17B4
GO:0072389	flavin adenine dinucleotide catabolic process	biological_process	1	69	2	14405	2.26E-05	0.000240489	104.384058	TERAL2
E3.5										
GO:0005753	mitochondrial proton-transporting ATP synthase complex	cellular_component	1	103	14	14405	0.004356462	0.00746682	9.989597781	ATP5A1W
GO:1903715	regulation of aerobic respiration	biological_process	1	103	2	14405	5.06E-05	0.000283326	69.92718447	TERAL2
GO:0006734	NADH metabolic process	biological_process	1	103	7	14405	0.001038714	0.002338643	19.97919556	TERAL2
GO:0032000	positive regulation of fatty acid beta-oxidation	biological_process	1	103	2	14405	5.06E-05	0.000283326	69.92718447	ACSL5
GO:1903862	positive regulation of oxidative phosphorylation	biological_process	1	103	2	14405	5.06E-05	0.000283326	69.92718447	TERAL2
GO:1904949	ATPase complex	cellular_component	1	103	2	14405	5.06E-05	0.000283326	69.92718447	TERAL2
GO:0045261	proton-transporting ATP synthase complex, catalytic core F(1)	cellular_component	1	103	5	14405	0.000499274	0.001428375	27.97087379	ATP5A1W
GO:2001171	positive regulation of ATP biosynthetic process	biological_process	1	103	5	14405	0.000499274	0.001428375	27.97087379	TERAL2
GO:0046034	ATP metabolic process	biological_process	1	103	27	14405	0.015819843	0.022130332	5.179791442	TERAL2
GO:0016887	ATPase activity	molecular_function	1	103	91	14405	0.138039617	0.153131412	1.536861197	TERAL2
GO:0004367	glycerol-3-phosphate dehydrogenase [NAD+] activity	molecular_function	1	103	2	14405	5.06E-05	0.000283326	69.92718447	GPD1L2
GO:0072389	flavin adenine dinucleotide catabolic process	biological_process	1	103	2	14405	5.06E-05	0.000283326	69.92718447	TERAL2
E4.5										
GO:0005753	mitochondrial proton-transporting ATP synthase complex	cellular_component	1	46	14	14405	0.000885936	0.00164018	22.36801242	ATP5A1W
GO:0006734	NADH metabolic process	biological_process	1	46	7	14405	0.000207381	0.000560746	44.73602484	TERAL2
GO:1903862	positive regulation of oxidative phosphorylation	biological_process	1	46	2	14405	9.98E-06	6.31E-05	156.576087	TERAL2
GO:1903715	regulation of aerobic respiration	biological_process	1	46	2	14405	9.98E-06	6.31E-05	156.576087	TERAL2
GO:1904949	ATPase complex	cellular_component	1	46	2	14405	9.98E-06	6.31E-05	156.576087	TERAL2
GO:0045261	proton-transporting ATP synthase complex, catalytic core F(1)	cellular_component	1	46	5	14405	9.92E-05	0.000342463	62.63043478	ATP5A1W
GO:2001171	positive regulation of ATP biosynthetic process	biological_process	1	46	5	14405	9.92E-05	0.000342463	62.63043478	TERAL2
GO:0046034	ATP metabolic process	biological_process	1	46	27	14405	0.003328133	0.005066157	11.59822866	TERAL2
GO:0016887	ATPase activity	molecular_function	1	46	91	14405	0.034131002	0.04019439	3.44123268	TERAL2
GO:0072389	flavin adenine dinucleotide catabolic process	biological_process	1	46	2	14405	9.98E-06	6.31E-05	156.576087	TERAL2
E5.5										
GO:0005753	mitochondrial proton-transporting ATP synthase complex	cellular_component	1	52	14	14405	0.001131252	0.002379346	19.78708791	ATP5A1W
GO:0045261	proton-transporting ATP synthase complex, catalytic core F(1)	cellular_component	1	52	5	14405	0.000126928	0.000546769	55.40384615	ATP5A1W
GO:0005753	mitochondrial proton-transporting ATP synthase complex	cellular_component	1	52	14	14405	0.001131252	0.002379346	19.78708791	ATP5A1W
GO:0030898	actin-dependent ATPase activity	molecular_function	1	52	14	14405	0.001131252	0.002379346	19.78708791	MYH11
E6.5										
GO:0005753	mitochondrial proton-transporting ATP synthase complex	cellular_component	1	102	14	14405	0.004274241	0.008424099	10.08753501	ATP5A1W
GO:1903715	regulation of aerobic respiration	biological_process	1	102	2	14405	4.97E-05	0.000352283	70.6127451	TERAL2
GO:0045333	cellular respiration	biological_process	1	102	15	14405	0.004909134	0.009426044	9.41503268	NDUFAF2
GO:0022904	respiratory electron transport chain	biological_process	1	102	12	14405	0.003128734	0.006603136	11.76879085	NDUFAF2
GO:1903862	positive regulation of oxidative phosphorylation	biological_process	1	102	2	14405	4.97E-05	0.000352283	70.6127451	TERAL2
GO:1904949	ATPase complex	cellular_component	1	102	2	14405	4.97E-05	0.000352283	70.6127451	TERAL2
GO:0045261	proton-transporting ATP synthase complex, catalytic core F(1)	cellular_component	1	102	5	14405	0.000489648	0.001720696	28.24509804	ATP5A1W
GO:2001171	positive regulation of ATP biosynthetic process	biological_process	1	102	5	14405	0.000489648	0.001720696	28.24509804	TERAL2
GO:0046034	ATP metabolic process	biological_process	1	102	27	14405	0.015530461	0.023516653	5.230573711	TERAL2
GO:0016887	ATPase activity	molecular_function	1	102	91	14405	0.135891541	0.155274843	1.551928464	TERAL2
GO:0006734	NADH metabolic process	biological_process	1	102	7	14405	0.001018781	0.002896915	20.17507003	TERAL2
GO:0072389	flavin adenine dinucleotide catabolic process	biological_process	1	102	2	14405	4.97E-05	0.000352283	70.6127451	TERAL2
E18.5										
GO:2000505	regulation of energy homeostasis	biological_process	3	2499	9	14405	0.054617602	0.1159263	1.921435241	NR4A3; PASK; PRKAA2
GO:0097009	energy homeostasis	biological_process	3	2499	13	14405	0.175898772	0.25848348	1.330224397	ACACB; EDN2; NR4A3
GO:0051901	positive regulation of mitochondrial depolarization	biological_process	3	2499	5	14405	0.003893353	0.017459255	3.458583433	MLLT11; MYOC; P2RX7
GO:0051902	negative regulation of mitochondrial depolarization	biological_process	1	2499	2	14405	0.030085852	0.070274844	2.882152861	IFI6
GO:0005753	mitochondrial proton-transporting ATP synthase complex	cellular_component	1	2499	14	14405	0.726695183	0.78316978	0.411736123	ATP5A1W
GO:0045333	cellular respiration	biological_process	1	2499	15	14405	0.762100191	0.814280247	0.384287048	NR4A3
GO:0009051	pentose-phosphate shunt, oxidative branch	biological_process	1	2499	2	14405	0.030085852	0.070274844	2.882152861	PGD
GO:1903862	positive regulation of oxidative phosphorylation	biological_process	1	2499	2	14405	0.030085852	0.070274844	2.882152861	TERAL2
GO:0006637	acyl-CoA metabolic process	biological_process	4	2499	15	14405	0.10292626	0.175979401	1.537148193	ACOT2L; ACSL6; BAAT; HNF4A
GO:0046949	fatty-acyl-CoA biosynthetic process	biological_process	1	2499	4	14405	0.141499926	0.214033228	1.441076431	CBR4
GO:1903578	regulation of ATP metabolic process	biological_process	1	2499	1	14405	0	0	5.764305722	DNM1L
GO:1904949	ATPase complex	cellular_component	1	2499	2	14405	0.030085852	0.070274844	2.882152861	TERAL2
GO:0043462	regulation of ATPase activity	biological_process	1	2499	4	14405	0.141499926	0.214033228	1.441076431	MYH7
GO:0016887	ATPase activity	molecular_function	19	2499	91	14405	0.151302971	0.224792012	1.20353636	ACTC1; CARNS1; DMC1; DNAH12; ENTPD2L; KIF12; KIF13B; KIF1A; KIF20AL; KIF20B; KIF20BL; KIF28P; KIF6; KIF9; LOC107050152; MYH7; MYO5B; TERAL2; TOR1BL
GO:0045261	proton-transporting ATP synthase complex, catalytic core F(1)	cellular_component	1	2499	5	14405	0.209473809	0.288310387	1.152861144	ATP5A1W
GO:2001171	positive regulation of ATP biosynthetic process	biological_process	1	2499	5	14405	0.209473809	0.288310387	1.152861144	TERAL2
GO:0030898	actin-dependent ATPase activity	molecular_function	3	2499	14	14405	0.214443364	0.293558823	1.235208369	MYH11; MYH7; MYO3A
GO:0032780	negative regulation of ATPase activity	biological_process	1	2499	6	14405	0.279707589	0.362029371	0.96071762	PXK
GO:0046034	ATP metabolic process	biological_process	4	2499	27	14405	0.51463422	0.593483663	0.853971218	ENPP1; LOC107049680; MYH7; TERAL2
GO:0032781	positive regulation of ATPase activity	biological_process	1	2499	15	14405	0.762100191	0.814280247	0.384287048	RGNL
GO:0070524	11-beta-hydroxysteroid dehydrogenase (NADP+) activity	molecular_function	2	2499	2	14405	0	0	5.764305722	HSD11B1a; HSD11B1b
GO:0047035	testosterone dehydrogenase (NAD+) activity	molecular_function	1	2499	1	14405	0	0	5.764305722	RDH7L
GO:0003845	11-beta-hydroxysteroid dehydrogenase [NAD(P)] activity	molecular_function	2	2499	3	14405	0.005215884	0.020955598	3.842870482	HSD11B1a; HSD11B1b
GO:0003955	NAD(P)H dehydrogenase (quinone) activity	molecular_function	1	2499	2	14405	0.030085852	0.070274844	2.882152861	CBR4
GO:0004367	glycerol-3-phosphate dehydrogenase [NAD+] activity	molecular_function	1	2499	2	14405	0.030085852	0.070274844	2.882152861	GPD1L2
GO:0016174	NAD(P)H oxidase activity	molecular_function	1	2499	2	14405	0.030085852	0.070274844	2.882152861	KMO
GO:0009435	NAD biosynthetic process	biological_process	4	2499	13	14405	0.059328675	0.124841755	1.77363253	HAAO; KMO; KYNU; NMNAT2
GO:0034628	de novo' NAD biosynthetic process from aspartate	biological_process	1	2499	3	14405	0.079825789	0.138156799	1.921435241	NMNAT2
GO:0006735	NADH regeneration	biological_process	1	2499	3	14405	0.079825789	0.138156799	1.921435241	PCK1
GO:0030283	testosterone dehydrogenase [NAD(P)] activity	molecular_function	1	2499	3	14405	0.079825789	0.138156799	1.921435241	SRD5A2
GO:0006734	NADH metabolic process	biological_process	2	2499	7	14405	0.105544985	0.175979401	1.646944492	MDH1B; TERAL2
GO:0043020	NADPH oxidase complex	cellular_component	2	2499	7	14405	0.105544985	0.175979401	1.646944492	NOX3; NOXO1
GO:0019674	NAD metabolic process	biological_process	2	2499	8	14405	0.147831249	0.220030231	1.441076431	KMO; NMNAT2
GO:0003951	NAD+ kinase activity	molecular_function	3	2499	16	14405	0.296895852	0.37885357	1.080807323	CERKL; DGKG; DGKH
GO:0034356	NAD biosynthesis via nicotinamide riboside salvage pathway	biological_process	1	2499	7	14405	0.34937263	0.429941971	0.823472246	LOC112530987
GO:0046500	S-adenosylmethionine metabolic process	biological_process	2	2499	4	14405	0.018151652	0.05976976	2.882152861	COMT; GAMT
GO:0015742	alpha-ketoglutarate transport	biological_process	1	2499	1	14405	0	0	5.764305722	LOC107049971
GO:0006771	riboflavin metabolic process	biological_process	1	2499	2	14405	0.030085852	0.070274844	2.882152861	ENPP1
GO:0072389	flavin adenine dinucleotide catabolic process	biological_process	1	2499	2	14405	0.030085852	0.070274844	2.882152861	TERAL2

Supplement 10-1. The FPKM values of energy metabolism related differentially expressed genes during different developmental stages.

gene_id	baseMean	lfcSE	stat	foldChange	log2FoldChange	pval	padj	up_down	expression_Female1	expression_Female2	expression_Female3	expression_Male1	expression_Male2	expression_Male3	Dbxref
ATP5A1W	2428.622188	0.134634625	-20.47607175	0.147953092	-2.756788245	3.51921E-93	4.61967E-89	Down	141.611	98.8841	94.965	19.9284	13.6101	16.9948	CGNC:54287,GeneID:431564
TERAL2	1039.589281	0.157289813	-17.82227719	0.143262939	-2.803262647	4.74644E-71	2.07688E-67	Down	21.3094	18.5292	14.944	3.02479	2.04532	2.40133	CGNC:54049,GeneID:430766
HSD17B4	418.46343	0.18929053	5.775875076	2.133642478	1.093318452	7.65542E-09	1.24065E-06	Up	4.74667	2.75725	3.49493	7.81111	7.81748	7.4418	CGNC:1559,GeneID:395785
ACSM4L	2.110957688	2.156214853	-1.973254675	0.052382872	-4.25476104	0.048466554	0	Down	0.101526	0.0178516	0.0168668	0	0	0	CGNC:52956,GeneID:426991
E3.5															
ATP5A1W	2948.546366	0.369253379	-21.82462592	0.0037502	-8.058816867	1.3544E-105	1.7774E-101	Down	143.82	149.161	151.432	0.582074	0.297074	0.705269	CGNC:54287,GeneID:431564
TERAL2	1139.552692	0.652573638	-13.59825201	0.00213158	-8.873860785	4.10118E-42	3.84427E-39	Down	21.838	21.9375	23.7197	0.0476004	0.0599198	0.0119002	CGNC:54049,GeneID:430766
ACSL5	29.33353089	0.504837855	-2.210719024	0.46135377	-1.11605465	0.0270553	0.423420726	Down	0.262366	0.643187	0.394388	0.127094	0.220912	0.183576	CGNC:52076,GeneID:423896
GPD1L2	106.1542601	0.335623161	-4.761697064	0.330303504	-1.598135822	1.91972E-06	7.97229E-05	Down	0.742704	3.86449	1.69876	0.501753	0.637115	0.678943	CGNC:13104,GeneID:424263
E4.5															
ATP5A1W	2934.755388	1.238533111	-2.841848147	0.087188216	-3.519723027	0.004485285	0.127237858	Down	139.808	138.949	105.828	70.6114	13.304	1.03398	CGNC:54287,GeneID:431564
TERAL2	1143.63901	1.319985924	-2.79660049	0.077402647	-3.691473283	0.005164335	0.141266558	Down	22.5197	20.324	16.7135	10.9407	1.93073	0.10001	CGNC:54049,GeneID:430766
E5.5															
ATP5A1W	2034.655747	0.24060753	-4.580713384	0.465820446	-1.102154132	4.63393E-06	0.00265717	Down	93.1656	73.9619	70.3962	38.4144	29.5457	45.1729	CGNC:54287,GeneID:431564
MYH11	113.2917682	0.614553427	2.189094562	2.540857675	1.345315566	0.028589968	0.999887727	Up	0.22849	0.274837	0.189624	0.220164	0.790776	1.17428	CGNC:4917,GeneID:396211
E6.5															
ATP5A1W	3268.065976	0.944160368	-4.161986732	0.065626262	-3.929582926	3.15491E-05	0.001722502	Down	180.87	144.122	140.743	29.1011	1.93985	15.9198	CGNC:54287,GeneID:431564
TERAL2	935.2341755	0.937174762	-4.023557658	0.073262732	-3.770776691	5.73255E-05	0.002883668	Down	20.5634	17.9601	16.2652	3.87142	0.261981	1.9606	CGNC:54049,GeneID:430766
NDUFAF2	386.7728715	0.154972462	6.671427607	2.047534219	1.033887563	2.53327E-11	5.67072E-09	Up	15.8971	17.8745	16.7785	32.0553	32.6271	38.4069	CGNC:66627,GeneID:769549
E18.5															
NR4A3	93.90498882	0.46307518	-3.429199097	0.332637566	-1.587976988	0.000605365	0.003335497	Down	1.8021	1.094	0.682435	0.297149	0.427509	0.441775	CGNC:10177,GeneID:420996
ACACB	412.4169959	0.264903602	4.014877887	2.090076304	1.063555613	5.94766E-05	0.000435362	Up	1.05154	0.83152	1.82391	2.10603	2.38151	2.29594	CGNC:53981,GeneID:430557
MLLT11	258.7214254	0.253005957	-4.373033563	0.464450405	-1.106403544	1.22532E-05	0.00010614	Down	11.5477	14.773	8.0295	4.09829	5.24564	3.97976	CGNC:16982,GeneID:395847
IFI6	144.0784286	0.517649575	-11.04088831	0.019033555	-5.715311142	2.42623E-28	4.30722E-26	Down	51.3497	52.621	16.2263	1.62728	0.508788	0	CGNC:49866,GeneID:403120
ATP5A1W	1271.634427	0.760993465	-10.86693367	0.003240281	-8.269665507	1.65676E-27	2.73111E-25	Down	74.4382	84.0237	38.9496	0.395955	0.0843353	0.171986	CGNC:54287,GeneID:431564
PGD	2713.259869	0.229973939	4.669701812	2.105130573	1.073909721	3.01637E-06	3.01171E-05	Up	28.3554	27.2321	51.9074	70.5085	75.7334	68.1006	CGNC:2017,GeneID:419450
TERAL2	1363.85238	0.6266959	-16.62873826	0.000729319	-10.42116209	4.31587E-62	3.8735E-59	Down	36.1139	35.1394	18.6825	0.0368453	0.0130616	0.0129694	CGNC:54049,GeneID:430766
ACOT2L	421.7229731	0.236460828	5.441156327	2.439558922	1.286620329	5.29358E-08	7.7532E-07	Up	3.34594	3.42688	4.09339	9.75239	9.38556	8.51945	CGNC:51932,GeneID:423247
CBR4	1639.816044	0.250574233	6.954165085	3.3462253	1.742534585	3.54656E-12	1.09971E-10	Up	5.16603	3.88489	8.6932	24.6932	25.1064	22.7089	CGNC:7388,GeneID:422435
DNM1L	3540.411378	0.217728313	4.794911322	2.061919418	1.043987952	1.62747E-06	1.7563E-05	Up	16.8817	15.9439	24.5287	42.7989	38.1424	36.5431	CGNC:9791,GeneID:418132
MYH7	342.9014152	0.844557818	-2.960284417	0.176760602	-2.500131347	0.003073551	0.013347641	Down	4.13482	1.17444	0.627323	0.164856	0.225806	0.240892	CGNC:49277,GeneID:395350
MYH11	1190.506098	0.277295749	7.160323966	3.960074031	1.985527401	8.04866E-13	2.70888E-11	Up	1.48969	0.878435	4.43303	4.4917	6.23677	12.3181	CGNC:4917,GeneID:396211
PXK	702.9916929	0.221229758	-7.666457285	0.308630279	-1.696048488	1.76812E-14	7.49711E-13	Down	13.9931	11.2736	8.50885	3.30113	3.44602	3.3085	CGNC:5325,GeneID:416065
ENPP1	774.4370893	0.223538797	5.077754588	2.196300036	1.135075154	3.81922E-07	4.80151E-06	Up	3.92956	3.18748	5.82137	11.4191	8.8847	10.3766	CGNC:2090,GeneID:426929
RGNL	8.266565499	1.38768175	-2.26793685	0.112877154	-3.147174577	0.023333056	0.070867742	Down	0.115534	0.230462	0.0595234	0.0849851	0.0150186	0	CGNC:53240,GeneID:428008
HSD11B1a	108.1929422	0.44177742	-6.396942526	0.141020347	-2.826024762	1.58519E-10	3.75916E-09	Down	7.79085	9.65443	4.44713	0.816949	0.486259	1.0235	CGNC:57151,GeneID:771930
RDH7L	21.33296315	0.681534645	-2.412870801	0.319867197	-1.644455046	0.015827429	0.052051002	Down	0.123755	0.150332	0.0679793	0.0487815	0.058139	0.0451674	CGNC:57208,GeneID:772005
GPD1L2	92.33345476	0.448334046	-8.617176693	0.06870821	-3.863373691	6.86252E-18	4.43456E-16	Down	2.12543	2.78069	1.65449	0.228723	0.0991705	0.111957	CGNC:13104,GeneID:424263
KMO	18.74978796	0.793928141	-3.084505446	0.183153392	-2.448875675	0.002038909	0.009357887	Down	0.113628	0.208261	0.729017	0	0	0.0878243	CGNC:5661,GeneID:424041
HAAO	25.89199663	0.69638943	-4.027464038	0.143121926	-2.804683387	5.63817E-05	0.000414775	Down	0.27918	1.13585	0.799479	0.0626301	0.0986447	0.167881	CGNC:7539,GeneID:421396
NMNAT2	55.54678888	0.546131303	-5.298475095	0.134561434	-2.893663108	1.16774E-07	1.62069E-06	Down	1.14605	1.92547	0.623292	0.199338	0.179615	0.0629682	CGNC:3436,GeneID:771393
PCK1	140.181788	0.469168018	-5.223455464	0.182924694	-2.450678247	1.75615E-07	2.36224E-06	Down	0.486569	2.14088	7.82656	0.204469	0.169458	1.34138	CGNC:7308,GeneID:396458
SRD5A2	203.5654446	0.265937364	-7.482525021	0.251759307	-1.989882982	7.2908E-14	2.83132E-12	Down	4.69931	4.81905	3.93567	1.3786	1.27683	1.12393	CGNC:8069,GeneID:772291
MDH1B	432.9828068	0.259447293	-10.41582292	0.153641828	-2.702357059	2.09974E-25	2.92425E-23	Down	16.9632	15.9147	10.9774	1.9789	2.09473	2.50339	CGNC:6485,GeneID:424097
NOX3	361.4332112	0.371195159	-7.97681634	0.128429131	-2.960955612	1.50156E-15	7.39564E-14	Down	1.07763	2.35315	0.554997	0.12075	0.150325	0.183603	CGNC:10233,GeneID:428609
CERKL	70.9640688	0.578232375	-8.694178268	0.030665168	-5.027255346	3.49349E-18	2.35155E-16	Down	1.49061	1.47007	0.899581	0.0438471	0.0228855	0.024068	CGNC:6767,GeneID:424120
LOC112530987	72.72633309	0.409150793	-3.370628662	0.384459787	-1.379095389	0.000749969	0.004015825	Down	4.44619	5.14938	5.20429	2.02637	1.97617	1.54476	GeneID:112530987
COMT	2909.30148	0.957235435	3.563417571	10.63707486	3.411029567	0.000366058	0.002144963	Up	3.57126	3.215	36.4154	86.8948	83.7332	67.6002	CGNC:1439,GeneID:416783
LOC107049971	104.851005	0.644009881	-4.01875478	0.166302552	-2.588117789	5.85065E-05	0.000429039	Down	2.36649	8.06935	43.2985	1.05724	1.95859	1.26741	CGNC:72987,GeneID:107049971
ACTC1	58.33634788	0.544762884	2.199495719	2.294537892	1.198203632	0.027842693	0.081544363	Up	1.13288	0.656927	2.26727	2.38744	3.90531	2.39214	CGNC:51944,GeneID:423298
CARNS1	41.34003764	0.516602053	-3.8860535	0.248696271	-2.007543216	0.000101887	0.000701016	Down	1.21035	0.837705	1.0369	0.215432	0.198048	0.192934	CGNC:61457,GeneID:100359387
DMC1	295.7424854	0.325794612	-13.06643879	0.052302533	-4.256975361	5.12072E-39	1.6545E-36	Down	11.1869	13.053	8.42623	0.249866	0.345018	0.556812	CGNC:9294,GeneID:427903
DNAH12	580.0969926	0.298566299	-5.87863206	0.296240045	-1.75516142	4.13671E-09	7.71692E-08	Down	3.68788	2.51479	1.31981	0.761927	0.539886	0.789036	CGNC:4170,GeneID:416004
ENTPD2L	217.4973742	0.383881001	-7.721682558	0.128139997	-2.964207228	1.14804E-14	4.98564E-13	Down	3.9721	4.49989	2.12699	0.419655	0.289028	0.567434	CGNC:53185,GeneID:427773
KIF12	30.58952365	0.624623847	-2.845712421	0.291688449	-1.777499839	0.004431219	0.018178352	Down	0.221593	0.982946	0.976448	0.162267	0.124838	0.255413	CGNC:2607,GeneID:427829
KIF13B	2593.697375	0.158720353	7.223256467	2.21372777	1.14647782	5.07572E-13	1.73725E-11	Up	6.05681	5.7704	6.09552	12.8122	13.9629	12.8579	CGNC:12468,GeneID:422026
KIF1A	207.792568	0.317759772	-6.084294061	0.261821614	-1.933343896	1.17006E-09	2.43272E-08	Down	1.34463	2.32342	1.01269	0.283468	0.412962	0.347467	CGNC:4671,GeneID:424849
KIF20AL	229.5223372	0.267591317	-4.994358944	0.395994669	-1.336447088	5.90315E-07	7.12214E-06	Down	1.86838	2.01816	1.55397	0.796862	0.765928	0.825546	CGNC:54047,GeneID:430761
KIF20B	951.5576633	0.243623533	-7.623931011	0.275979113	-1.857369011	2.46065E-14	1.0219E-12	Down	7.80692	8.52983	6.29693	1.78947	1.55834	2.43878	CGNC:54416,GeneID:423793
KIF20BL	118.1899276	0.402615073	-5.752967771	0.200791271	-2.316231541	8.76902E-09	1.53482E-07	Down	0.768009	0.947629	0.886455	0.282423	0.179447	0.110985	CGNC:52053,GeneID:768380
KIF28P	76.38214588	0.410517454	-3.299109044	0.391113206	-1.354341846	0.000969922	0.0050077	Down	0.490279	0.54308	0.306653	0.239484	0.163097	0.161978	CGNC:68232,GeneID:101748750
KIF6	36.24663335	0.564050295	-3.079964828	0.299939812	-1.737255068	0.00207025	0.009485223	Down	0.400405	0.666486	0.249683	0.106673	0.196201	0.15731	CGNC:7648,GeneID:428572
KIF9	317.2557242	0.301716318	-6.588214707	0.252127977	-1.987771882	4.45147E-11	1.13607E-09	Down	5.2984	4.60801	3.47069	0.968764	1.08808	1.21391	CGNC:4109,GeneID:420405
LOC107050152	160.2197399	0.31977278	-4.258526287	0.3891071	-1.361760791	2.05779E-05	0.000168254	Down	1.99816	2.44711	1.10436	0.680395	0.671877	0.47755	CGNC:73167,GeneID:107050152
MYO5B	30.03387364	0.63995474	-2.440996722	0.338651332	-1.562127424	0.014646788	0.048827663	Down	0.551436	0.210775	0.250578	0.155499	0.049235	0.164324	CGNC:9827,GeneID:430317
TOR1BL	55.2911179	0.499271397	-5.347874396	0.157122237	-2.670040723	8.89932E-08	1.25672E-06	Down	2.13469	4.61004	2.48053	0.529824	0.672048	0.222148	CGNC:50458,GeneID:417192
PASK	383.1614415	0.287747608	-3.709445972	0.477183411	-1.067384205	0.000207713	0.001303213	Down	6.49637	4.28328	2.64192	2.21622	1.9042	2.09494	CGNC:4507,GeneID:424845
EDN2	80.14968573	0.512051959	-3.470262237	0.291798713	-1.776954576	0.00051995	0.002920654	Down	2.16321	3.13291	1.63324	0.69599	0.359893	0.763308	CGNC:421,GeneID:419559
MYOC	420.6395	0.388695537	3.106662004	2.309444147	1.207545656	0.001892126	0.00879833	Up	5.54587	5.67275	20.089	13.7043	24.2088	16.1243	CGNC:14423,GeneID:424391
PRKAA2	106.2871607	0.372644121	4.671262276	3.342015505	1.740718427	2.99354E-06	2.99077E-05	Up	0.221279	0.171097	0.415127	0.820011	0.728447	0.987422	CGNC:8225,GeneID:429110
P2RX7	68.80873625	0.510748147	-7.769460463	0.063891263	-3.968237531	7.88211E-15	3.5273E-13	Down	2.01541	2.53231	1.0724	0.0502733	0.123152	0.0526313	CGNC:2831,GeneID:771952
BAAT	15.51211583	0.818957447	-2.004294091	0.32053825	-1.641431572	0.045038566	0.120184678	Down	0.331864	0.635259	1.84873	0.265949	0.191094	0.362455	CGNC:55610,GeneID:769879
HNF4A	23.8213962	0.808920555	-2.353879394	0.267182713	-1.904101426	0.018578638	0.059126607	Down	0.0993508	0.403626	0.885643	0.0837417	0.0384096	0.122061	CGNC:50941,GeneID:419198
ACSL6	54.66792474	0.540383533	-3.775941366	0.243086801	-2.040456535	0.000159404	0.001030897	Down	0.509495	0.79585	0.282811	0.0784593	0.104167	0.144417	CGNC:50224,GeneID:416324
MYO3A	204.160988	0.354004486	-7.253624106	0.168659387	-2.567815475	4.05765E-13	1.4158E-11	Down	2.18052	1.56488	0.848952	0.301025	0.17881	0.258998	CGNC:5772,GeneID:420494
LOC107049680	145.2808806	0.317045011	-4.19300437	0.39794167	-1.329371119	2.75284E-05	0.000218751	Down	3.9633	2.96822	1.83632	1.66742	0.739354	0.970279	CGNC:72696,GeneID:107049680
HSD11B1b	20.58918906	0.842707397	-3.009996719	0.172354814	-2.536546501	0.002612505	0.011594786	Down	0.880185	0.815857	1.52899	0.264619	0.188015	0.0596132	CGNC:937,GeneID:419861
KYNU	62.74774957	0.583125516	2.294261658	2.527730252	1.337842514	0.021775474	0.067211077	Up	0.101656	0.0892317	0.377575	0.45409	0.304777	0.377193	CGNC:9432,GeneID:424302
DGKH	186.6862163	0.329837281	3.407098846	2.179184309	1.123788221	0.000656573	0.003582217	Up	0.685992	0.55559	0.301246	1.3579	0.961405	1.44165	CGNC:12728,GeneID:769506
NOXO1	86.60683102	0.514434003	-8.701641265	0.044922432	-4.47642015	3.27118E-18	2.22042E-16	Down	2.47647	3.35857	2.00162	0.116729	0.0883871	0.0853633	CGNC:4149,GeneID:416546
DGKG	195.5128997	0.273792939	4.777593904	2.476103254	1.308071476	1.77405E-06	1.89674E-05	Up	0.891452	0.767423	1.42401	2.50961	1.59169	3.0141	CGNC:4267,GeneID:424839
GAMT	161.885328	0.330425089	-4.146858241	0.386830646	-1.370226002	3.37069E-05	0.000262631	Down	5.58083	11.5661	14.1784	2.82108	4.6164	3.39027	CGNC:66651,GeneID:770737
