# Supplementary material for: Key events in the process of sex determination and differentiation in early chicken embryos
Source: Anim Biosci. 2025 Feb 27;38(6):1081–104. doi: 10.5713/ab.24.0679 (PMC12061580; doi:10.5713/ab.24.0679)
Supplement: Supplementary file 11 [file ab-24-0679-Supplementary-11.pdf]

Supplement 11. energy related genes partial enrichment terms.

Term_ID	Term_description	ListHits	FoldEnrichmen	p-value	q-value	geneID
GO:0097009	energy homeostasis	5	54.1212121	2.82E-08	2.98E-06	NR4A3;ACACB;PASK;EDN2;PRKAA2
gga00061	Fatty acid biosynthesis	4	39.5793651	2.31E-06		ACSL5;ACACB;CBR4;ACSL6
GO:0006637	acyl-CoA metabolic process	3	48.7090909	2.92E-05	0.00115596	BAAT;HNF4A;ACSL6
GO:0046034	ATP metabolic process	3	25.9781818	0.00020358	0.00570838	MYH7;ENPP1;LOC107049680
GO:0008210	estrogen metabolic process	2	86.5939394	0.00021609	0.00570838	HSD17B4;COMT
GO:0045722	positive regulation of gluconeogenesis	2	64.9454545	0.00040138	0.00795163	PCK1;HNF4A
gga00380	Tryptophan metabolism	3	14.4411197	0.00108416		KMO;HAAO;KYNU
GO:0045333	cellular respiration	2	32.4727273	0.00168653	0.0213852	NDUFAF2;NR4A3
gga00030	Pentose phosphate pathway	2	14.2485714	0.00842112		PGD;RGNL
GO:0009749	response to glucose	2	14.0422604	0.00888881	0.07719869	ACSL5;HNF4A
gga01040	Biosynthesis of unsaturated fatty acids	2	11.8738095	0.01200135		HSD17B4;BAAT
gga00071	Fatty acid degradation	2	10.7943723	0.01441701		ACSL5;ACSL6
gga00564	Glycerophospholipid metabolism	3	5.74539171	0.01468076		GPD1L2;DGKH;DGKG
gga00140	Steroid hormone biosynthesis	2	9.62741313	0.01793588		SRD5A2;COMT
gga00620	Pyruvate metabolism	2	9.62741313	0.01793588		ACACB;PCK1
gga00330	Arginine and proline metabolism	2	8.68815331	0.02178096		CARNS1;GAMT
GO:0046325	negative regulation of glucose import	1	28.8646465	0.03412519	0.09714203	ENPP1
GO:0006734	NADH metabolic process	1	25.9781818	0.03784556	0.09714203	MDH1B
GO:0030539	male genitalia development	1	25.9781818	0.03784556	0.09714203	SRD5A2
gga00740	Riboflavin metabolism	1	22.2633929	0.0440743		ENPP1
GO:0035035	histone acetyltransferase binding	1	19.9832168	0.04892244	0.10408331	NR4A3
