# Supplementary material for: Key events in the process of sex determination and differentiation in early chicken embryos
Source: Anim Biosci. 2025 Feb 27;38(6):1081–104. doi: 10.5713/ab.24.0679 (PMC12061580; doi:10.5713/ab.24.0679)
Supplement: Supplementary file 12 [file ab-24-0679-Supplementary-12.pdf]

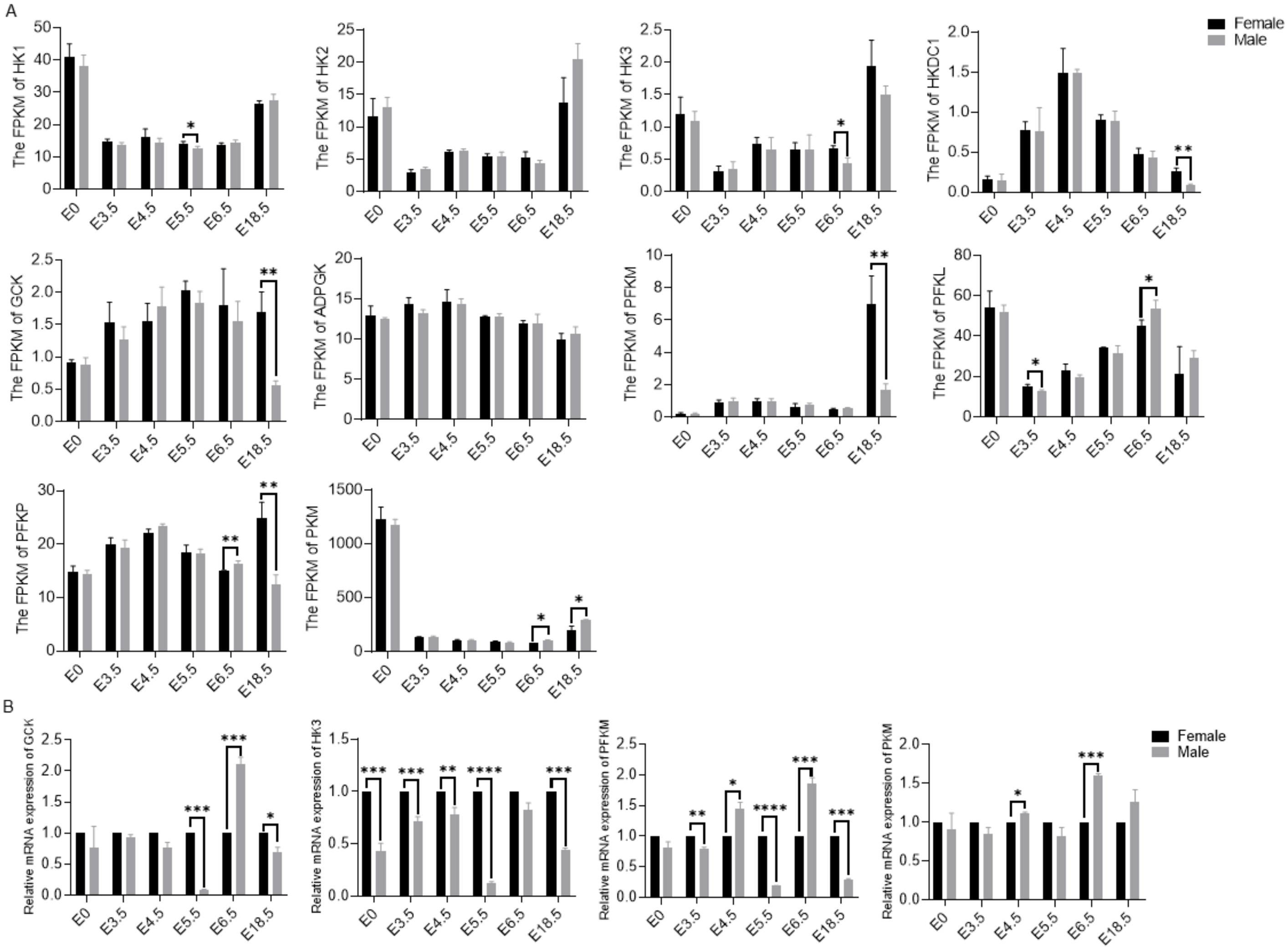

Supplement 12. A. The FPKM value of key enzymes in the glycolysis process (HK1, HK2, HK3, HKDC1, GCK, ADPGK, PFKM, PFKL, PFKP and PKM) at E0-E18.5. *$p < 0.05$, significant difference; **$p < 0.01$, extremely significant difference. B. The relative expression level of gender-related genes (GCK, HK3, PFKM and PKM) at E0-E18.5 were detected by qRT-PCR.
