# Supplementary material for: Key events in the process of sex determination and differentiation in early chicken embryos
Source: Anim Biosci. 2025 Feb 27;38(6):1081–104. doi: 10.5713/ab.24.0679 (PMC12061580; doi:10.5713/ab.24.0679)
Supplement: Supplementary file 13 [file ab-24-0679-Supplementary-13.pdf]

Supplement 13. Distribution statistics of GO items related to epigenetic modification and corresponding related genes in different periods in male and female.

id	term	category	ListHits	ListTotal	PopHits	PopTotal	pval	padj	Enrichment_score	Gene
E0										
GO:0018024	histone-lysine N-methyltransferase activity	molecular_function	1	69	24	14405	0.005830423	0.009191491	8.698671498	PRDM6
GO:0031065	positive regulation of histone deacetylation	biological_process	1	69	10	14405	0.000992648	0.002341812	20.87681159	NIPBLL
GO:0000118	histone deacetylase complex	cellular_component	1	69	26	14405	0.006823343	0.010485412	8.029542921	HINTW
GO:0042826	histone deacetylase binding	molecular_function	1	69	61	14405	0.034515888	0.04227723	3.42242813	NIPBLL
E3.5										
GO:0031065	positive regulation of histone deacetylation	biological_process	1	103	10	14405	0.002194844	0.004260909	13.98543689	NIPBLL
GO:0042826	histone deacetylase binding	molecular_function	2	103	61	14405	0.009464806	0.014405434	4.585389145	KLF4; NIPBLL
GO:0000118	histone deacetylase complex	cellular_component	1	103	26	14405	0.014715903	0.02085438	5.37901419	HINTW
E4.5										
GO:0051571	positive regulation of histone H3-K4 methylation	biological_process	1	46	9	14405	0.000354065	0.000799935	34.79468599	GCG
GO:0019213	deacetylase activity	molecular_function	1	46	5	14405	9.92E-05	0.000342463	62.63043478	AADAC
GO:0031065	positive regulation of histone deacetylation	biological_process	1	46	10	14405	0.000441681	0.000950423	31.31521739	NIPBLL
GO:0000118	histone deacetylase complex	cellular_component	1	46	26	14405	0.003087862	0.00480724	12.04431438	HINTW
GO:0042826	histone deacetylase binding	molecular_function	1	46	61	14405	0.016199423	0.020114692	5.133642195	NIPBLL
E5.5										
GO:0031065	positive regulation of histone deacetylation	biological_process	1	52	10	14405	0.000564607	0.001431823	27.70192308	NIPBLL
GO:0000118	histone deacetylase complex	cellular_component	1	52	26	14405	0.003929856	0.006355869	10.6545858	HINTW
GO:0042826	histone deacetylase binding	molecular_function	1	52	61	14405	0.020420915	0.026750204	4.541298865	NIPBLL
E6.5										
GO:0051567	histone H3-K9 methylation	biological_process	1	102	3	14405	0.000148263	0.000721932	47.0751634	PRDM8
GO:0046974	histone methyltransferase activity (H3-K9 specific)	molecular_function	1	102	5	14405	0.000489648	0.001720696	28.24509804	PRDM8
GO:0042054	histone methyltransferase activity	molecular_function	1	102	7	14405	0.001018781	0.002896915	20.17507003	PRDM8
GO:0016571	histone methylation	biological_process	1	102	9	14405	0.001730398	0.00425461	15.69172113	PRDM8
GO:0035097	histone methyltransferase complex	cellular_component	1	102	14	14405	0.004274241	0.008424099	10.08753501	PRDM8
GO:0031065	positive regulation of histone deacetylation	biological_process	1	102	10	14405	0.002153023	0.004950625	14.12254902	NIPBLL
GO:0000118	histone deacetylase complex	cellular_component	1	102	26	14405	0.01444606	0.022282226	5.431749623	HINTW
GO:0042826	histone deacetylase binding	molecular_function	1	102	61	14405	0.069401929	0.084890598	2.31517197	NIPBLL
GO:0006338	chromatin remodeling	biological_process	1	102	55	14405	0.057858616	0.072935141	2.567736185	BRDT
E18.5										
GO:0040029	regulation of gene expression, epigenetic	biological_process	1	2499	5	14405	0.209473809	0.288310387	1.152861144	PRDM14
GO:0045814	negative regulation of gene expression, epigenetic	biological_process	1	2499	12	14405	0.642491128	0.70826385	0.48035881	TRIM27.1
GO:0061085	regulation of histone H3-K27 methylation	biological_process	1	2499	1	14405	0	0	5.764305722	GATA3
GO:0034971	histone H3-R17 methylation	biological_process	1	2499	1	14405	0	0	5.764305722	NR1H4
GO:0034972	histone H3-R26 methylation	biological_process	1	2499	1	14405	0	0	5.764305722	PRDM14
GO:0051570	regulation of histone H3-K9 methylation	biological_process	1	2499	1	14405	0	0	5.764305722	SETD7
GO:0006211	5-methylcytosine catabolic process	biological_process	1	2499	1	14405	0	0	5.764305722	TET2
GO:0090308	regulation of methylation-dependent chromatin silencing	biological_process	1	2499	1	14405	0	0	5.764305722	UHRF2
GO:0051569	regulation of histone H3-K4 methylation	biological_process	2	2499	4	14405	0.018151652	0.05976976	2.882152861	GATA3; GFI1
GO:1901536	negative regulation of DNA demethylation	biological_process	1	2499	2	14405	0.030085852	0.070274844	2.882152861	GATA3
GO:0010216	maintenance of DNA methylation	biological_process	1	2499	2	14405	0.030085852	0.070274844	2.882152861	UHRF2
GO:0070989	oxidative demethylation	biological_process	2	2499	5	14405	0.0395391	0.086681368	2.305722289	CYP1A2; CYP3A5
GO:0043046	DNA methylation involved in gamete generation	biological_process	4	2499	14	14405	0.07953729	0.138156799	1.646944492	FKBP6; LOC107049165; TDRD5; TDRKH
GO:0042799	histone methyltransferase activity (H4-K20 specific)	molecular_function	1	2499	3	14405	0.079825789	0.138156799	1.921435241	KMT5C
GO:0016428	tRNA (cytosine-5-)-methyltransferase activity	molecular_function	1	2499	3	14405	0.079825789	0.138156799	1.921435241	NSUN6
GO:0051567	histone H3-K9 methylation	biological_process	1	2499	3	14405	0.079825789	0.138156799	1.921435241	PRDM8
GO:0016571	histone methylation	biological_process	2	2499	9	14405	0.194438073	0.281466166	1.280956827	PRDM8; SATB1
GO:0046974	histone methyltransferase activity (H3-K9 specific)	molecular_function	1	2499	5	14405	0.209473809	0.288310387	1.152861144	PRDM8
GO:0034773	histone H4-K20 trimethylation	biological_process	1	2499	6	14405	0.279707589	0.362029371	0.96071762	KMT5C
GO:0032259	methylation	biological_process	2	2499	11	14405	0.29515248	0.378164116	1.048055586	ARMT1; GSTO1
GO:0051568	histone H3-K4 methylation	biological_process	1	2499	7	14405	0.34937263	0.429941971	0.823472246	DYDC1
GO:0042054	histone methyltransferase activity	molecular_function	1	2499	7	14405	0.34937263	0.429941971	0.823472246	PRDM8
GO:0044030	regulation of DNA methylation	biological_process	1	2499	8	14405	0.41655309	0.49677813	0.720538215	PRDM14
GO:0051571	positive regulation of histone H3-K4 methylation	biological_process	1	2499	9	14405	0.480014524	0.557946095	0.640478414	GCG
GO:0080111	DNA demethylation	biological_process	1	2499	9	14405	0.480014524	0.557946095	0.640478414	TET2
GO:0080182	histone H3-K4 trimethylation	biological_process	1	2499	10	14405	0.53902526	0.614313836	0.576430572	TET2
GO:0018024	histone-lysine N-methyltransferase activity	molecular_function	3	2499	24	14405	0.618893365	0.689155719	0.720538215	KMT5C; SETD7; SMYD1
GO:0008757	S-adenosylmethionine-dependent methyltransferase activity	molecular_function	1	2499	14	14405	0.726695183	0.78316978	0.411736123	ARMT1
GO:0035097	histone methyltransferase complex	cellular_component	1	2499	14	14405	0.726695183	0.78316978	0.411736123	PRDM8
GO:0008168	methyltransferase activity	molecular_function	1	2499	32	14405	0.982727613	0.996924078	0.180134554	PRDM14
GO:0035064	methylated histone binding	molecular_function	1	2499	34	14405	0.987568526	0.999684073	0.169538404	RAG2
GO:2000617	positive regulation of histone H3-K9 acetylation	biological_process	3	2499	3	14405	0	0	5.764305722	CEBPB; GATA3; LOC107049165
GO:0071442	positive regulation of histone H3-K14 acetylation	biological_process	2	2499	4	14405	0.018151652	0.05976976	2.882152861	GATA3; LOC107049165
GO:0003985	acetyl-CoA C-acetyltransferase activity	molecular_function	1	2499	2	14405	0.030085852	0.070274844	2.882152861	ACAT2
GO:0035065	regulation of histone acetylation	biological_process	1	2499	3	14405	0.079825789	0.138156799	1.921435241	MYOCD
GO:0019213	deacetylase activity	molecular_function	1	2499	5	14405	0.209473809	0.288310387	1.152861144	NDST4
GO:0031065	positive regulation of histone deacetylation	biological_process	2	2499	10	14405	0.243971582	0.329943948	1.152861144	ERD2L; NIPBLL
GO:0035067	negative regulation of histone acetylation	biological_process	1	2499	6	14405	0.279707589	0.362029371	0.96071762	ERD2L
GO:0035035	histone acetyltransferase binding	molecular_function	2	2499	11	14405	0.29515248	0.378164116	1.048055586	CEBPB; NR4A3
GO:0035066	positive regulation of histone acetylation	biological_process	1	2499	8	14405	0.41655309	0.49677813	0.720538215	ISL1
GO:0042826	histone deacetylase binding	molecular_function	10	2499	61	14405	0.496336235	0.574147822	0.944968151	BHLHE41; C6H10ORF90; CEBPB; HEY2; HOXA10; LEF1; MEF2C; NACC2; NIPBLL; SKOR2
GO:0032041	NAD-dependent histone deacetylase activity (H3-K14 specific)	molecular_function	1	2499	10	14405	0.53902526	0.614313836	0.576430572	HDAC11
GO:0000118	histone deacetylase complex	cellular_component	3	2499	26	14405	0.68398	0.74700828	0.665112199	HDAC11; HINTW; SATB2
GO:0016575	histone deacetylation	biological_process	2	2499	20	14405	0.699863046	0.758961059	0.576430572	HDAC11; SALL1
GO:0043967	histone H4 acetylation	biological_process	2	2499	20	14405	0.699863046	0.758961059	0.576430572	LEF1; MYOD1
GO:0004407	histone deacetylase activity	molecular_function	2	2499	26	14405	0.853644365	0.892870615	0.443408132	HDAC11; NACC2
GO:0043966	histone H3 acetylation	biological_process	2	2499	27	14405	0.87117098	0.907750535	0.426985609	LEF1; MYOD1

Supplement 13-1. The FPKM values of epigenetic modification related differentially expressed genes during different developmental stages.

gene_id	baseMean	lfcSE	stat	foldChange	log2FoldChange	pval	padj	up_down	expression_Female1	expression_Female2	expression_Female3	expression_Male1	expression_Male2	expression_Male3	Dbxref
E0															
PRDM6	27.11350842	0.531561039	3.450066644	3.565046787	1.833921011	0.000560448	0.023504803	Up	0.263475	0.10916	0.103923	0.708993	0.469155	0.480789	CGNC:3984,GeneID:426800
NIPBLL	330.9817484	0.215191042	-11.3288839	0.184555376	-2.437874335	9.43999E-30	1.23919E-26	Down	2.50642	1.83089	1.70549	0.406979	0.227011	0.462546	CGNC:52967,GeneID:427025
HINTW	13611.63125	0.327196579	-8.386782348	0.149257311	-2.744126493	4.99629E-17	3.45191E-14	Down	298.781	278.674	257.26	61.2866	29.6831	41.1404	CGNC:49311,GeneID:395423
E3.5															
NIPBLL	749.9024884	1.284388638	-7.835852794	0.000934006	-10.0642803	4.65671E-15	4.46059E-13	Down	4.5383	4.76952	4.78093	0.0208601	0	0	CGNC:52967,GeneID:427025
KLF4	208.2035007	0.20080455	5.468660901	2.140773244	1.09813199	4.53448E-08	2.22953E-06	Up	2.84448	3.09948	2.78718	7.07889	6.44623	4.8215	CGNC:28582,GeneID:770254
HINTW	5718.257781	0.479584639	-16.49871035	0.004150417	-7.912528044	3.74812E-61	1.22967E-57	Down	150.649	134.875	146.508	1.15581	0.604101	0.279767	CGNC:49311,GeneID:395423
E4.5															
GCG	34.89753552	0.483327027	2.494792321	2.306652306	1.205800555	0.012603087	0	Up	0.217562	0.186408	0.189405	0.750866	0.39302	0.245994	CGNC:8432,GeneID:396196
AADAC	162.9587966	0.224570703	-4.707694004	0.480560457	-1.057210153	2.50535E-06	0.000156288	Down	2.89977	1.8324	1.9844	0.935991	0.878009	1.10505	CGNC:7888,GeneID:425034
NIPBLL	840.9073573	1.375863791	-2.810547864	0.068539	-3.86693104	0.004945723	0.137131416	Down	4.69998	5.52719	4.67902	2.23972	0.405738	0.0149829	CGNC:52967,GeneID:427025
HINTW	6490.771064	1.322765183	-2.734735119	0.081479876	-3.617412401	0.006243049	0.163887089	Down	142.707	142.313	120.838	71.2853	15.3965	0.983141	CGNC:49311,GeneID:395423
E5.5															
NIPBLL	550.6752879	0.152353844	-7.161645272	0.469401974	-1.091104187	7.97145E-13	1.56719E-09	Down	3.23672	2.76181	2.41457	1.41113	1.10731	1.48843	CGNC:52967,GeneID:427025
HINTW	4673.962282	0.087423855	-13.99360766	0.428279603	-1.223375126	1.70537E-44	2.34693E-40	Down	106.586	78.6911	82.1279	44.5264	29.8253	38.6833	CGNC:49311,GeneID:395423
E6.5															
PRDM8	30.43606422	0.569680934	3.041906953	3.32399081	1.732916394	0.002350846	0.061668371	Up	0.439868	0.438875	1.39438	2.25965	2.0734	1.0851	CGNC:58878,GeneID:777386
NIPBLL	573.7461618	1.023768743	-4.22831067	0.04976198	-4.328812301	2.35453E-05	0.001351437	Down	4.16078	3.76027	2.98858	0.590675	0.0330186	0.246503	CGNC:52967,GeneID:427025
HINTW	5123.457518	1.062986504	-3.612416616	0.069832861	-3.839950108	0.000303357	0.011542163	Down	113.445	121.329	88.0379	25.6856	1.09607	11.603	CGNC:49311,GeneID:395423
BRDT	12.68620477	0.939096929	-3.544073031	0.099564269	-3.3282281	0.000393996	0	Down	0.151385	0.183486	0.141843	0	0.0529374	0.0212034	CGNC:4518,GeneID:424506
E18.5															
PRDM14	82.02233398	0.520845565	-9.224690106	0.035781584	-4.804638929	2.84382E-20	2.3203E-18	Down	0.872194	0.754205	0.639791	0.0252733	0.0267164	0.0263671	CGNC:64389,GeneID:100858709
TRIM27.1	20.13042507	0.794626472	-2.085142431	0.31711777	-1.656909374	0.037056374	0.102666047	Down	0.246002	0.293818	0.400863	0.211613	0.0481417	0.044392	CGNC:58,GeneID:417042
GATA3	39.20737637	0.551651136	-4.334348839	0.190643803	-2.391048459	1.46192E-05	0.000124564	Down	0.74262	0.848998	0.76227	0.189228	0.118463	0.126845	CGNC:5080,GeneID:419106
NR1H4	19.38042438	0.835829661	-3.032462975	0.172584822	-2.534622501	0.002425669	0.010873106	Down	0.421599	0.690443	0.576487	0.140224	0.0210299	0.145172	CGNC:8812,GeneID:373902
SETD7	1391.303585	0.174354531	5.944999149	2.051298631	1.036537537	2.76458E-09	5.39394E-08	Up	7.22366	6.57979	7.74374	15.7584	14.164	14.04	CGNC:7445,GeneID:422443
TET2	1868.091605	0.215609216	5.780429609	2.372345111	1.246313898	7.45101E-09	1.31986E-07	Up	2.0732	1.38011	1.95796	3.75627	4.36911	4.5424	CGNC:51773,GeneID:422540
UHRF2	2367.688018	0.219596782	-4.93745547	0.471637608	-1.084249332	7.91485E-07	9.27898E-06	Down	27.9966	33.7057	20.8065	11.5387	12.1965	14.3776	CGNC:11194,GeneID:431601
CYP1A2	28.42851365	0.837607926	-2.802305685	0.19652252	-2.347233454	0.005073877	0.0203851	Down	0.316922	0.212153	2.67028	0.161648	0.0993698	0.0940811	CGNC:49932,GeneID:396051
FKBP6	340.8429863	0.322745117	-3.451578165	0.462017692	-1.113979998	0.000557318	0.003101439	Down	5.63505	4.03647	2.51379	1.93396	1.34836	1.99921	CGNC:50514,GeneID:417460
KMT5C	818.9419996	0.221446574	-7.657195235	0.308713473	-1.695659649	1.90038E-14	8.0368E-13	Down	40.6149	57.7921	39.3086	11.2781	11.3314	13.7984	CGNC:67572,GeneID:101748081
NSUN6	157.2417667	0.296832817	-3.888391305	0.449314603	-1.154202143	0.000100911	0.000695187	Down	3.13828	2.54898	2.20556	1.43102	0.977264	1.08085	CGNC:6499,GeneID:428419
PRDM8	5.300953844	1.804632087	-2.708827392	0.033762444	-4.888436831	0.006752146	0.025830195	Down	0.410243	0.497311	0.683256	0	0.0558324	0	CGNC:58878,GeneID:777386
ARMT1	1944.239591	0.212011625	-7.277847352	0.343173905	-1.542988242	3.39189E-13	1.19904E-11	Down	33.4796	28.9452	16.7045	10.1542	8.12041	8.98035	CGNC:9819,GeneID:421637
DYDC1	153.3421129	0.693382656	-10.45745813	0.00656486	-7.251020096	1.3544E-25	1.91933E-23	Down	8.83734	12.7236	9.64289	0.0430522	0	0.0908144	CGNC:1735,GeneID:423627
GCG	151.0612144	0.310532592	-4.970873183	0.343024105	-1.543618136	6.6652E-07	7.97012E-06	Down	1.54878	2.09002	1.59023	0.708197	0.744664	0.853236	CGNC:8432,GeneID:396196
RAG2	112.6293534	0.334919389	-6.114187007	0.241859351	-2.04775978	9.70504E-10	2.0468E-08	Down	1.11501	0.647464	0.673136	0.213596	0.139923	0.14375	CGNC:6005,GeneID:423165
CEBPB	388.0123919	0.25952886	-5.802750797	0.352090621	-1.5059813	6.52357E-09	1.16968E-07	Down	24.9434	38.1234	17.8	7.99008	9.29802	6.89785	CGNC:6065,GeneID:396185
ACAT2	3110.397905	0.291233545	5.101482384	2.80057449	1.485722802	3.37003E-07	4.29913E-06	Up	36.5798	43.2012	107.479	150.481	147.752	152.521	CGNC:8861,GeneID:421587
MYOCD	723.3422507	0.190691116	5.910449887	2.184147498	1.127070287	3.41175E-09	6.51498E-08	Up	2.88233	3.28591	4.82466	6.18597	7.44127	8.57901	CGNC:685,GeneID:427790
NDST4	72.96124869	0.475678989	-7.116268085	0.095718446	-3.385059208	1.10889E-12	3.65593E-11	Down	0.470006	0.872897	0.247894	0.031045	0.0698283	0.037622	CGNC:9106,GeneID:422686
ERD2L	61.81541451	0.543870716	-7.730781622	0.054238243	-4.204545736	1.06888E-14	4.66697E-13	Down	1.66979	1.28967	1.05125	0.0723394	0.101036	0.0642947	CGNC:53397,GeneID:428538
ISL1	31.33486903	1.20231328	-5.028482163	0.015136643	-6.045810883	4.94377E-07	6.07351E-06	Down	0.367208	1.5776	0	0.0110794	0	0	CGNC:11048,GeneID:396383
HDAC11	1103.259797	0.268515017	-4.473337431	0.434925958	-1.201158277	7.70081E-06	7.02465E-05	Down	30.432	17.5916	14.6504	6.71768	8.48025	9.05216	CGNC:3790,GeneID:415978
LEF1	679.1358979	0.202551288	-7.700807876	0.33919609	-1.559808554	1.35209E-14	5.84036E-13	Down	16.0561	23.3508	11.7423	5.49056	6.21042	4.31141	CGNC:7997,GeneID:395865
BHLHE41	499.8263501	0.249080951	4.070623329	2.019383236	1.013914729	4.68875E-05	0.000352147	Up	8.02792	5.93501	10.4541	11.8998	16.6743	18.1799	CGNC:70134,GeneID:101750689
C6H10ORF90	46.48303737	0.500793734	-2.438408267	0.428943763	-1.221139581	0.014752102	0.04915846	Down	0.679105	0.91063	0.796826	0.421674	0.250369	0.300335	CGNC:52096,GeneID:423959
HEY2	132.8812259	0.325261081	-4.24014392	0.384444223	-1.379153796	2.23377E-05	0.000181066	Down	5.25244	3.01921	3.40594	1.56902	1.16045	1.60508	CGNC:66409,GeneID:421718
HOXA10	185.9789053	0.330756696	3.331252529	2.14627372	1.101834079	0.000864561	0.004527387	Up	1.34437	0.810545	0.689909	1.9097	1.61058	2.29428	CGNC:16063,GeneID:776143
MEF2C	398.3008231	0.264914065	5.561935298	2.776822365	1.47343489	2.66799E-08	4.13641E-07	Up	0.954613	1.01256	1.78953	3.52944	2.74263	3.46108	CGNC:10854,GeneID:769007
NACC2	311.0948013	0.26942546	4.018209938	2.11786123	1.082608062	5.86419E-05	0.000429642	Up	4.96779	4.98668	7.275	11.063	11.5612	9.71981	CGNC:1213,GeneID:417128
NIPBLL	477.8266388	1.212963015	-9.285788517	0.000406821	-11.26331803	1.60514E-20	1.33665E-18	Down	4.18106	3.82256	1.60036	0	0.00379762	0	CGNC:52967,GeneID:427025
SKOR2	10.67177482	1.194136353	-2.895061666	0.091056235	-3.457098379	0.003790841	0.015952339	Down	0.0467108	0.211909	0.0407589	0.00763406	0.00807715	0.0230414	CGNC:6011,GeneID:770237
GFI1	255.505304	0.282512162	-9.368691834	0.159677243	-2.646769381	7.3437E-21	6.41284E-19	Down	8.90222	7.50049	5.21049	1.37236	0.915041	1.16053	CGNC:4440,GeneID:429088
CYP3A5	16.365044	1.022165152	-2.134864209	0.220341966	-2.1821838	0.032772104	0.092980916	Down	0.703584	0.901897	0.174362	0.248994	0	0.0580109	CGNC:49925,GeneID:414832
LOC107049165	1482.697477	0.239275019	-6.453339705	0.34290409	-1.544122983	1.09412E-10	2.66197E-09	Down	54.1528	46.8369	30.2887	12.9571	11.8497	13.9158	CGNC:72186,GeneID:107049165
TDRD5	778.8966557	0.238949965	-4.616584608	0.465504581	-1.103132729	3.90107E-06	3.8038E-05	Down	16.6487	11.192	8.30486	5.87407	5.30304	5.4181	CGNC:3034,GeneID:424421
TDRKH	1871.36812	0.235686484	-8.522387313	0.248512186	-2.008611497	1.56298E-17	9.63736E-16	Down	65.232	56.6986	37.7947	11.8954	10.2909	12.0482	CGNC:16943,GeneID:425667
SATB1	241.3727966	0.271544904	6.378328932	3.321886355	1.732002718	1.79031E-10	4.22842E-09	Up	0.491126	0.619192	1.23027	2.17105	2.27523	3.04277	CGNC:8550,GeneID:420647
GSTO1	383.2042937	0.253514279	-12.28407076	0.115487824	-3.114187346	1.10305E-34	2.82854E-32	Down	23.4431	30.9614	17.7347	1.91755	3.64301	2.13661	CGNC:52072,GeneID:423881
SMYD1	72.7490822	0.673246225	-7.973824271	0.024208423	-5.368347093	1.53838E-15	7.55397E-14	Down	1.83293	1.96825	1.19289	0.0975165	0.0345964	0	CGNC:11893,GeneID:373960
NR4A3	93.90498882	0.46307518	-3.429199097	0.332637566	-1.587976988	0.000605365	0.003335497	Down	1.8021	1.094	0.682435	0.297149	0.427509	0.441775	CGNC:10177,GeneID:420996
HINTW	1734.474094	0.442570028	-21.34999681	0.001430893	-9.448868691	3.8997E-101	2.09996E-97	Down	32.0635	52.047	27.0949	0.0680875	0.125716	0.0355991	CGNC:49311,GeneID:395423
SALL1	2343.426352	0.223134809	-9.465996479	0.231295112	-2.112193314	2.90775E-21	2.62429E-19	Down	39.0761	38.8265	24.7806	7.55559	6.92216	8.59143	CGNC:49323,GeneID:395446
MYOD1	4.615695714	1.723171378	-2.180914081	0.073909892	-3.758088721	0.029189772	0.08475167	Down	0.239743	0.29648	0.181337	0.032968	0	0.0326284	CGNC:4667,GeneID:374048
SATB2	311.0416262	0.32735744	-4.67848507	0.345908676	-1.531536897	2.89002E-06	2.9035E-05	Down	5.75649	2.69371	2.17717	1.11363	1.1438	1.09101	CGNC:6166,GeneID:424063
ACTL6B	13.53453227	0.988024144	-2.353557612	0.199523178	-2.325371746	0.018594726	0.059147727	Down	0.374288	0.949576	0.0716913	0.0966168	0.068932	0.127051	CGNC:67318,GeneID:101747825
BRDT	32.64727625	0.581509623	-2.954714311	0.303928779	-1.718194805	0.003129587	0.013540032	Down	0.610771	0.369952	0.312441	0.123941	0.108772	0.100557	CGNC:4518,GeneID:424506
SOX9	1143.930014	0.777871055	3.561479486	6.822837265	2.770371806	0.000368771	0.002158512	Up	2.9005	5.15846	24.2053	43.5844	41.5151	40.3798	CGNC:49063,GeneID:374148
