# Supplementary material for: Key events in the process of sex determination and differentiation in early chicken embryos
Source: Anim Biosci. 2025 Feb 27;38(6):1081–104. doi: 10.5713/ab.24.0679 (PMC12061580; doi:10.5713/ab.24.0679)
Supplement: Supplementary file 15 [file ab-24-0679-Supplementary-15.pdf]

Supplement 15. Epigenetic modification related genes partial enrichment terms.

Term_ID	Term_description	ListHits	FoldEnrichmen	p-value	q-value	geneID
gga00310	Lysine degradation	4	34.2161235	3.55E-06		PRDM6;SETD7;ACAT2;SMYD1
gga04068	FoxO signaling pathway	2	7.43219076	0.028259688		SETD7;RAG2
GO:0045814	negative regulation of gene expression, epigenetic	1	25.0666667	0.03917825	0.06776434	TRIM27.1
GO:0043046	DNA methylation involved in gamete generation	3	80.5714286	6.19E-06	0.00020547	FKBP6;TDRD5;TDRKH
GO:0051569	regulation of histone H3-K4 methylation	2	150.4	6.85E-05	0.00110271	GATA3;GFI1
GO:0016571	histone methylation	1	62.6666667	0.015854478	0.05071523	PRDM8
GO:0080111	DNA demethylation	1	62.6666667	0.015854478	0.05071523	TET2
GO:0044030	regulation of DNA methylation	1	47	0.02108471	0.05231767	PRDM14
GO:0051571	positive regulation of histone H3-K4 methylation	1	47	0.02108471	0.05231767	GCG
GO:0016575	histone deacetylation	2	44.2352941	0.00091342	0.00702937	HDAC11;SALL1
GO:0043967	histone H4 acetylation	2	41.7777778	0.001025876	0.007782	LEF1;MYOD1
GO:0043966	histone H3 acetylation	2	27.8518519	0.002318245	0.01431382	LEF1;MYOD1
GO:0035066	positive regulation of histone acetylation	1	62.6666667	0.015854478	0.05071523	ISL1
GO:0006338	chromatin remodeling	3	20.8888889	0.000392028	0.00385495	GATA3;SMYD1;SATB2
GO:0008584	male gonad development	2	17.4883721	0.005806539	0.02964685	GATA3;HOXA10
GO:0001541	ovarian follicle development	1	19.7894737	0.049370777	0.07598807	CEBPB
GO:0071392	cellular response to estradiol stimulus	1	22.1176471	0.044287744	0.07280741	MYOD1
GO:0010828	positive regulation of glucose transmembrane transport	1	75.2	0.013229169	0.05017635	NR4A3
GO:0035774	positive regulation of insulin secretion involved in cellular response to glucose stimulus	1	19.7894737	0.049370777	0.07598807	GCG
GO:0045333	cellular respiration	1	23.5	0.041736313	0.07013285	NR4A3
GO:0003988	acetyl-CoA C-acyltransferase activity	1	75.2	0.013229169	0.05017635	ACAT2
