# Supplementary material for: Key events in the process of sex determination and differentiation in early chicken embryos
Source: Anim Biosci. 2025 Feb 27;38(6):1081–104. doi: 10.5713/ab.24.0679 (PMC12061580; doi:10.5713/ab.24.0679)
Supplement: Supplementary file 16 [file ab-24-0679-Supplementary-16.pdf]

Supplement 16. Acetylation related genes partial enrichment terms.

Term_ID	Term_description	ListHits	FoldEnrichmen	p-value	q-value	geneID
GO:0016575	histone deacetylation	2	80.0448179	0.00027612	0.00211181	HDAC11;SALL1
GO:0043967	histone H4 acetylation	2	75.5978836	0.00031036	0.00233053	LEF1;MYOD1
GO:0004407	histone deacetylase activity	2	68.0380952	0.00038473	0.00273957	HDAC11;NACC2
GO:0043966	histone H3 acetylation	2	50.3985891	0.00070635	0.00448804	LEF1;MYOD1
GO:0008584	male gonad development	2	31.6456257	0.00179163	0.0090237	GATA3;HOXA10
GO:0006338	chromatin remodeling	2	25.1992945	0.00281173	0.01350286	GATA3;SATB2
GO:0051569	regulation of histone H3-K4 methylation	1	136.07619	0.00732828	0.02631807	GATA3
GO:0003988	acetyl-CoA C-acyltransferase activity	1	136.07619	0.00732828	0.02631807	ACAT2
GO:0035066	positive regulation of histone acetylation	1	113.396825	0.00878778	0.0268841	ISL1
GO:0008406	gonad development	1	113.396825	0.00878778	0.0268841	SALL1
GO:0001541	ovarian follicle development	1	35.8095238	0.02757634	0.04249637	CEBPB
GO:0043627	response to estrogen	1	34.0190476	0.02900748	0.04309384	GATA3
GO:0097009	energy homeostasis	1	28.3492063	0.03471199	0.04700345	NR4A3
gga00380	Tryptophan metabolism	1	26.9567568	0.03656451		ACAT2
gga00620	Pyruvate metabolism	1	26.9567568	0.03656451		ACAT2
gga00280	Valine, leucine and isoleucine degradation	1	23.1953488	0.04239172		ACAT2
GO:0006635	fatty acid beta-oxidation	1	21.2619048	0.04602541	0.05783982	ACAT2
