# Supplementary material for: Key events in the process of sex determination and differentiation in early chicken embryos
Source: Anim Biosci. 2025 Feb 27;38(6):1081–104. doi: 10.5713/ab.24.0679 (PMC12061580; doi:10.5713/ab.24.0679)
Supplement: Supplementary file 17 [file ab-24-0679-Supplementary-17.pdf]

Supplement 17. Distribution statistics of GO items related to histone acetylation and corresponding related genes in different periods in male and female.

| id | term | category | ListHits | ListTotal | PopHits | PopTotal | pval | padj | Enrichment_score | Gene |
|---|---|---|---|---|---|---|---|---|---|---|
| E0 | | | | | | | | | | |
| GO:0031065 | positive regulation of histone deacetylation | biological_process | 1 | 69 | 10 | 14405 | 0.000992648 | 0.002341812 | 20.87681159 | NIPBLL |
| GO:0000118 | histone deacetylase complex | cellular_component | 1 | 69 | 26 | 14405 | 0.006823343 | 0.010485412 | 8.029542921 | HINTW |
| GO:0042826 | histone deacetylase binding | molecular_function | 1 | 69 | 61 | 14405 | 0.034515888 | 0.04227723 | 3.42242813 | NIPBLL |
| E3.5 | | | | | | | | | | |
| GO:0031065 | positive regulation of histone deacetylation | biological_process | 1 | 103 | 10 | 14405 | 0.002194844 | 0.004260909 | 13.98543689 | NIPBLL |
| GO:0042826 | histone deacetylase binding | molecular_function | 2 | 103 | 61 | 14405 | 0.009464806 | 0.014405434 | 4.585389145 | KLF4; NIPBLL |
| GO:0000118 | histone deacetylase complex | cellular_component | 1 | 103 | 26 | 14405 | 0.014715903 | 0.02085438 | 5.37901419 | HINTW |
| E4.5 | | | | | | | | | | |
| GO:0019213 | deacetylase activity | molecular_function | 1 | 46 | 5 | 14405 | 9.92E-05 | 0.000342463 | 62.63043478 | AADAC |
| GO:0031065 | positive regulation of histone deacetylation | biological_process | 1 | 46 | 10 | 14405 | 0.000441681 | 0.000950423 | 31.31521739 | NIPBLL |
| GO:0000118 | histone deacetylase complex | cellular_component | 1 | 46 | 26 | 14405 | 0.003087862 | 0.00480724 | 12.04431438 | HINTW |
| GO:0042826 | histone deacetylase binding | molecular_function | 1 | 46 | 61 | 14405 | 0.016199423 | 0.020114692 | 5.133642195 | NIPBLL |
| E5.5 | | | | | | | | | | |
| GO:0031065 | positive regulation of histone deacetylation | biological_process | 1 | 52 | 10 | 14405 | 0.000564607 | 0.001431823 | 27.70192308 | NIPBLL |
| GO:0000118 | histone deacetylase complex | cellular_component | 1 | 52 | 26 | 14405 | 0.003929856 | 0.006355869 | 10.6545858 | HINTW |
| GO:0042826 | histone deacetylase binding | molecular_function | 1 | 52 | 61 | 14405 | 0.020420915 | 0.026750204 | 4.541298865 | NIPBLL |
| E6.5 | | | | | | | | | | |
| GO:0031065 | positive regulation of histone deacetylation | biological_process | 1 | 102 | 10 | 14405 | 0.002153023 | 0.004950625 | 14.12254902 | NIPBLL |
| GO:0000118 | histone deacetylase complex | cellular_component | 1 | 102 | 26 | 14405 | 0.01444606 | 0.022282226 | 5.431749623 | HINTW |
| GO:0042826 | histone deacetylase binding | molecular_function | 1 | 102 | 61 | 14405 | 0.069401929 | 0.084890598 | 2.31517197 | NIPBLL |
| E18.5 | | | | | | | | | | |
| GO:2000617 | positive regulation of histone H3-K9 acetylation | biological_process | 3 | 2499 | 3 | 14405 | 0 | 0 | 5.764305722 | CEBPB; GATA3; LOC107049165 |
| GO:0071442 | positive regulation of histone H3-K14 acetylation | biological_process | 2 | 2499 | 4 | 14405 | 0.018151652 | 0.05976976 | 2.882152861 | GATA3; LOC107049165 |
| GO:0003985 | acetyl-CoA C-acetyltransferase activity | molecular_function | 1 | 2499 | 2 | 14405 | 0.030085852 | 0.070274844 | 2.882152861 | ACAT2 |
| GO:0035065 | regulation of histone acetylation | biological_process | 1 | 2499 | 3 | 14405 | 0.079825789 | 0.138156799 | 1.921435241 | MYOCD |
| GO:0019213 | deacetylase activity | molecular_function | 1 | 2499 | 5 | 14405 | 0.209473809 | 0.288310387 | 1.152861144 | NDST4 |
| GO:0031065 | positive regulation of histone deacetylation | biological_process | 2 | 2499 | 10 | 14405 | 0.243971582 | 0.329943948 | 1.152861144 | ERD2L; NIPBLL |
| GO:0035067 | negative regulation of histone acetylation | biological_process | 1 | 2499 | 6 | 14405 | 0.279707589 | 0.362029371 | 0.96071762 | ERD2L |
| GO:0035035 | histone acetyltransferase binding | molecular_function | 2 | 2499 | 11 | 14405 | 0.29515248 | 0.378164116 | 1.048055586 | CEBPB; NR4A3 |
| GO:0035066 | positive regulation of histone acetylation | biological_process | 1 | 2499 | 8 | 14405 | 0.41655309 | 0.49677813 | 0.720538215 | ISL1 |
| GO:0042826 | histone deacetylase binding | molecular_function | 10 | 2499 | 61 | 14405 | 0.496336235 | 0.574147822 | 0.944968151 | BHLHE41; C6H10ORF90; CEBPB; HEY2; HOXA10; LEF1; MEF2C; NACC2; NIPBLL; SKOR2 |
| GO:0032041 | NAD-dependent histone deacetylase activity (H3-K14 specific) | molecular_function | 1 | 2499 | 10 | 14405 | 0.53902526 | 0.614313836 | 0.576430572 | HDAC11 |
| GO:0000118 | histone deacetylase complex | cellular_component | 3 | 2499 | 26 | 14405 | 0.68398 | 0.74700828 | 0.665112199 | HDAC11; HINTW; SATB2 |
| GO:0016575 | histone deacetylation | biological_process | 2 | 2499 | 20 | 14405 | 0.699863046 | 0.758961059 | 0.576430572 | HDAC11; SALL1 |
| GO:0043967 | histone H4 acetylation | biological_process | 2 | 2499 | 20 | 14405 | 0.699863046 | 0.758961059 | 0.576430572 | LEF1; MYOD1 |
| GO:0004407 | histone deacetylase activity | molecular_function | 2 | 2499 | 26 | 14405 | 0.853644365 | 0.892870615 | 0.443408132 | HDAC11; NACC2 |
| GO:0043966 | histone H3 acetylation | biological_process | 2 | 2499 | 27 | 14405 | 0.87117098 | 0.907750535 | 0.426985609 | LEF1; MYOD1 |

Supplement 17-1. The FPKM values of histone acetylation related differentially expressed genes during different developmental stages.

| gene_id | baseMean | lfcSE | stat | foldChange | log2FoldChange | pval | padj | expression_Female1 | expression_Female2 | expression_Female3 | expression_Male1 | expression_Male2 | expression_Male3 | Dbxref |
|---|---|---|---|---|---|---|---|---|---|---|---|---|---|---|
| E0 | | | | | | | | | | | | | | |
| HDAC1 | 4413.744806 | 0.11377109 | 0.028290171 | 1.002233456 | 0.003218604 | 0.97743072 | 0.999940151 | 59.7524 | 59.3605 | 55.6382 | 60.5539 | 56.0938 | 56.8292 | CGNC:2394,GeneID:373961 |
| HDAC10 | 723.1583442 | 0.167454089 | 0.007439292 | 1.000863854 | 0.00124574 | 0.994064358 | 0.999940151 | 13.1476 | 7.40321 | 7.36359 | 8.89374 | 9.18477 | 7.92085 | CGNC:50586,GeneID:417742 |
| HDAC11 | 257.2447073 | 0.208303856 | -0.643206885 | 0.911312351 | -0.133982474 | 0.520089867 | 0.999940151 | 4.12429 | 2.72556 | 3.42719 | 3.05129 | 3.4186 | 2.83462 | CGNC:3790,GeneID:415978 |
| HDAC2 | 7866.204653 | 0.113429297 | -0.153122837 | 0.988033172 | -0.017368616 | 0.878301407 | 0.999940151 | 111.01 | 106.355 | 112.773 | 108.151 | 112.161 | 105.42 | CGNC:11140,GeneID:395635 |
| HDAC3 | 2855.905766 | 0.118646654 | -0.265770198 | 0.978380297 | -0.031532745 | 0.790416196 | 0.999940151 | 54.8023 | 58.7478 | 52.4089 | 53.183 | 55.873 | 55.5835 | CGNC:1896,GeneID:395506 |
| HDAC4 | 331.9766179 | 0.180753857 | -0.707402117 | 0.915184383 | -0.127865661 | 0.479316621 | 0.999940151 | 1.53515 | 1.57844 | 1.37009 | 1.31937 | 1.56765 | 1.21904 | CGNC:49084,GeneID:374207 |
| HDAC7 | 1099.043689 | 0.15237683 | 0.15160079 | 1.016140889 | 0.023100448 | 0.87950181 | 0.999940151 | 4.84669 | 5.76232 | 6.50577 | 5.73113 | 4.90498 | 5.96363 | CGNC:4743,GeneID:426885 |
| HDAC8 | 2476.977278 | 0.121678517 | -1.157670186 | 0.90697608 | -0.140863592 | 0.246998655 | 0.999940151 | 56.1751 | 62.5906 | 58.0343 | 53.4179 | 51.9825 | 53.505 | CGNC:3582,GeneID:422182 |
| HDAC9 | 62.44857283 | 0.366167555 | -0.664342241 | 0.84483378 | -0.243260574 | 0.506471291 | 0.999940151 | 0.354847 | 0.205828 | 0.202627 | 0.191839 | 0.180353 | 0.218937 | CGNC:51296,GeneID:420599 |
| HAT1 | 2448.692407 | 0.120154154 | 0.840552492 | 1.072513549 | 0.100995873 | 0.400598683 | 0.999940151 | 59.6182 | 57.7959 | 65.6762 | 66.2753 | 67.2791 | 68.5427 | CGNC:7250,GeneID:374037 |
| SIRT2 | 1092.635223 | 0.138981066 | -0.741229281 | 0.931083954 | -0.103016836 | 0.458554433 | 0.999940151 | 32.0925 | 35.2093 | 32.8863 | 32.9556 | 31.9543 | 28.6783 | CGNC:54341,GeneID:548628 |
| SIRT3 | 550.1719624 | 0.154752328 | -0.423333255 | 0.955606249 | -0.065511807 | 0.672052134 | 0.999940151 | 8.96657 | 10.5816 | 9.33518 | 9.21264 | 10.0699 | 8.70393 | CGNC:3086,GeneID:422988 |
| SIRT6 | 980.4321247 | 0.143864843 | -1.327164852 | 0.876039385 | -0.190932363 | 0.184454156 | 0.999940151 | 24.8899 | 33.0212 | 32.4322 | 25.8265 | 24.8715 | 26.5985 | CGNC:850,GeneID:428332 |
| SIRT7 | 313.2845628 | 0.191478524 | -0.523065936 | 0.932932176 | -0.100155893 | 0.60092837 | 0.999940151 | 5.45239 | 7.30034 | 7.64717 | 6.72316 | 6.16006 | 6.73211 | CGNC:71990,GeneID:10321419 |
| MORF4L1 | 3412.178846 | 0.115337254 | 0.531872643 | 1.043437892 | 0.06134473 | 0.594814206 | 0.999940151 | 62.9674 | 62.7175 | 66.9438 | 66.6299 | 69.4439 | 68.6981 | CGNC:54344,GeneID:554283 |
| E3.5 | | | | | | | | | | | | | | |
| HDAC1 | 1707.018446 | 0.079322304 | -0.290111644 | 0.984175615 | -0.023012324 | 0.771730828 | 0.999910028 | 21.8147 | 19.6755 | 20.6552 | 20.5385 | 19.6094 | 20.0117 | CGNC:2394,GeneID:373961 |
| HDAC10 | 1378.334061 | 0.088308766 | 0.548940345 | 1.034172068 | 0.048476245 | 0.58304639 | 0.999910028 | 13.4628 | 15.0452 | 14.0619 | 15.6948 | 14.5894 | 14.3875 | CGNC:50586,GeneID:417742 |
| HDAC11 | 484.4136978 | 0.142702221 | -0.967033142 | 0.908779517 | -0.137997777 | 0.333527466 | 0.999910028 | 5.21187 | 5.21396 | 5.99974 | 5.50107 | 5.42284 | 4.34879 | CGNC:3790,GeneID:415978 |
| HDAC2 | 8189.069053 | 0.055827196 | 0.957290443 | 1.037738426 | 0.053442841 | 0.338420678 | 0.999910028 | 98.2001 | 94.9422 | 95.1157 | 99.9812 | 101.228 | 105.271 | CGNC:11140,GeneID:395635 |
| HDAC3 | 1618.007801 | 0.084468773 | -0.463825175 | 0.973208791 | -0.039178743 | 0.642773005 | 0.999910028 | 27.8627 | 28.9257 | 29.1917 | 27.4883 | 25.7949 | 27.867 | CGNC:1896,GeneID:395506 |
| HDAC4 | 1288.540858 | 0.088341965 | -0.10628257 | 0.993513027 | -0.009389211 | 0.91535816 | 0.999910028 | 5.77557 | 5.58032 | 5.68682 | 5.70299 | 5.61652 | 5.52345 | CGNC:49084,GeneID:374207 |
| HDAC7 | 500.9855833 | 0.130533707 | -0.069769916 | 0.993707166 | -0.009107326 | 0.944376793 | 0.999910028 | 2.63748 | 2.66143 | 2.38241 | 2.75722 | 2.32353 | 2.1055 | CGNC:4743,GeneID:426885 |
| HDAC8 | 1051.21614 | 0.100759242 | -1.406682281 | 0.90642764 | -0.141736241 | 0.159521622 | 0.999910028 | 25.7168 | 25.62 | 25.2506 | 21.6563 | 21.0749 | 25.3303 | CGNC:3582,GeneID:422182 |
| HDAC9 | 549.9158144 | 0.124814675 | -0.792500196 | 0.933734533 | -0.098915654 | 0.428069078 | 0.999910028 | 2.27019 | 2.00218 | 1.99842 | 1.89999 | 1.90245 | 2.06046 | CGNC:51296,GeneID:420599 |
| HAT1 | 3236.125261 | 0.069631203 | 0.346645412 | 1.01687147 | 0.024137337 | 0.728857725 | 0.999910028 | 77.2613 | 80.2888 | 79.5267 | 76.9728 | 83.6754 | 84.6538 | CGNC:7250,GeneID:374037 |
| SIRT2 | 1056.885007 | 0.096629024 | 2.043548007 | 1.146682552 | 0.19746605 | 0.04099822 | 0.549560411 | 32.4596 | 36.3085 | 35.5698 | 38.6363 | 38.3703 | 37.1425 | CGNC:54341,GeneID:548628 |
| SIRT3 | 628.0249022 | 0.130833509 | 0.728829346 | 1.068328479 | 0.095355301 | 0.466106058 | 0.999910028 | 9.18036 | 11.6178 | 9.59006 | 10.9733 | 10.0877 | 9.73575 | CGNC:3086,GeneID:422988 |
| SIRT6 | 699.8764526 | 0.122434064 | 0.011854969 | 1.001006576 | 0.001451452 | 0.990541325 | 0.999910028 | 18.428 | 20.4428 | 18.9084 | 20.566 | 17.5433 | 18.2505 | CGNC:850,GeneID:428332 |
| SIRT7 | 363.6123847 | 0.149084976 | 1.095000217 | 1.11980543 | 0.163248081 | 0.273516541 | 0.999910028 | 6.95582 | 8.78906 | 6.7787 | 8.08995 | 7.83227 | 7.55873 | CGNC:71990,GeneID:10321419 |
| MORF4L1 | 4964.278158 | 0.064185765 | -1.162461997 | 0.949596477 | -0.074613512 | 0.245047853 | 0.999910028 | 78.6375 | 83.3101 | 82.1223 | 77.0146 | 74.0127 | 84.3902 | CGNC:54344,GeneID:554283 |
| E4.5 | | | | | | | | | | | | | | |
| HDAC1 | 1887.281846 | 0.073530652 | 0.513147218 | 1.026498877 | 0.03773205 | 0.607848344 | 0.999951946 | 24.2752 | 23.8472 | 24.8047 | 25.4841 | 23.9675 | 25.6581 | CGNC:2394,GeneID:373961 |
| HDAC10 | 1163.240591 | 0.089295365 | 0.565263965 | 1.03560616 | 0.050475452 | 0.571894235 | 0.999951946 | 12.2502 | 13.5296 | 13.6527 | 12.7218 | 13.2247 | 13.2949 | CGNC:50586,GeneID:417742 |
| HDAC11 | 525.9820915 | 0.121817278 | -0.738937547 | 0.939512746 | -0.090015361 | 0.459944921 | 0.999951946 | 6.26731 | 6.76679 | 5.89237 | 5.83165 | 6.01626 | 5.47212 | CGNC:3790,GeneID:415978 |
| HDAC2 | 7912.72023 | 0.053003694 | -0.124583319 | 0.995433347 | -0.006603376 | 0.900853435 | 0.999951946 | 98.7113 | 106.843 | 105.438 | 101.983 | 103.791 | 101.865 | CGNC:11140,GeneID:395635 |
| HDAC3 | 1118.620759 | 0.08938616 | 1.356708729 | 1.087692674 | 0.121270983 | 0.174873778 | 0.999951946 | 21.3776 | 22.0465 | 20.6159 | 22.5235 | 24.7928 | 23.3591 | CGNC:1896,GeneID:395506 |
| HDAC4 | 1149.562773 | 0.089356959 | 0.621446623 | 1.039241235 | 0.055530581 | 0.534305804 | 0.999951946 | 4.96236 | 5.45941 | 5.17609 | 5.55478 | 5.34421 | 5.1016 | CGNC:49084,GeneID:374207 |
| HDAC7 | 808.0410395 | 0.103032115 | 0.700481106 | 1.051298275 | 0.07217205 | 0.483626901 | 0.999951946 | 4.71693 | 4.35164 | 4.1934 | 4.65477 | 4.3225 | 4.71472 | CGNC:4743,GeneID:426885 |
| HDAC8 | 976.6184928 | 0.095072178 | -1.260100632 | 0.920314898 | -0.119800512 | 0.207633062 | 0.999951946 | 26.5933 | 23.8744 | 24.2768 | 23.1691 | 21.6629 | 23.9982 | CGNC:3582,GeneID:422182 |
| HDAC9 | 438.0652133 | 0.135709952 | 0.366030446 | 1.035030997 | 0.049673974 | 0.714342357 | 0.999951946 | 1.81389 | 1.49757 | 1.73757 | 1.82998 | 1.73493 | 1.58457 | CGNC:51296,GeneID:420599 |
| HAT1 | 2171.572822 | 0.073369274 | 0.485416905 | 1.024993447 | 0.035614686 | 0.627380643 | 0.999951946 | 54.1333 | 61.1495 | 55.8325 | 56.5967 | 60.6854 | 55.2115 | CGNC:7250,GeneID:374037 |
| SIRT2 | 1024.8343 | 0.092663718 | -1.029259055 | 0.936028933 | -0.095374971 | 0.303357958 | 0.999951946 | 37.4206 | 38.8875 | 36.9988 | 36.2636 | 35.6479 | 35.2889 | CGNC:54341,GeneID:548628 |
| SIRT3 | 476.3143615 | 0.127882033 | -0.410726625 | 0.964247526 | -0.052524556 | 0.681273001 | 0.999951946 | 10.0039 | 8.4683 | 10.1671 | 8.31259 | 8.80938 | 8.86088 | CGNC:3086,GeneID:422988 |
| SIRT6 | 563.0984733 | 0.117786594 | 0.008214816 | 1.000670911 | 0.000967595 | 0.993445599 | 0.999951946 | 16.901 | 16.784 | 16.3909 | 17.6734 | 15.6599 | 17.0757 | CGNC:850,GeneID:428332 |
| SIRT7 | 329.4986735 | 0.148776831 | 0.635700975 | 1.067752722 | 0.094577576 | 0.524971341 | 0.999951946 | 7.63526 | 7.72305 | 7.66809 | 8.7194 | 7.54067 | 9.17792 | CGNC:71990,GeneID:10321419 |
| MORF4L1 | 4320.315215 | 0.059428318 | 0.005270922 | 1.000217146 | 0.000313242 | 0.995794432 | 0.999951946 | 78.8025 | 83.4387 | 82.9418 | 79.4353 | 82.3084 | 79.7114 | CGNC:54344,GeneID:554283 |
| E5.5 | | | | | | | | | | | | | | |
| HDAC1 | 1506.374836 | 0.090299164 | -0.038506987 | 0.997592726 | -0.003477149 | 0.969283461 | 0.999887727 | 22.1744 | 21.5964 | 21.592 | 21.3693 | 21.4837 | 22.7157 | CGNC:2394,GeneID:373961 |
| HDAC10 | 893.0781645 | 0.115633575 | 0.852571788 | 1.070723465 | 0.098585924 | 0.393896813 | 0.999887727 | 10.1813 | 11.2736 | 10.2493 | 10.8091 | 10.947 | 11.8489 | CGNC:50586,GeneID:417742 |
| HDAC11 | 519.9630405 | 0.158898533 | -0.148210177 | 0.983808633 | -0.02355038 | 0.8821769 | 0.999887727 | 7.85711 | 7.06246 | 5.71979 | 6.14943 | 6.6471 | 7.43069 | CGNC:3790,GeneID:415978 |
| HDAC2 | 6126.287035 | 0.069567543 | -0.684582302 | 0.967527982 | -0.047624709 | 0.493607538 | 0.999887727 | 89.519 | 85.5064 | 94.4169 | 88.1845 | 79.3295 | 91.5705 | CGNC:11140,GeneID:395635 |
| HDAC3 | 957.2249106 | 0.104679296 | -0.103457211 | 0.99252144 | -0.010829828 | 0.917600108 | 0.999887727 | 23.0935 | 21.4835 | 21.2326 | 20.9815 | 20.4906 | 22.685 | CGNC:1896,GeneID:395506 |
| HDAC4 | 953.9211076 | 0.105529711 | 1.175246108 | 1.089769775 | 0.124023383 | 0.239896269 | 0.999887727 | 4.42478 | 5.17575 | 4.38364 | 4.90983 | 5.26049 | 4.93238 | CGNC:49084,GeneID:374207 |
| HDAC7 | 676.8787035 | 0.120768062 | 0.831273394 | 1.072064181 | 0.100391277 | 0.405819201 | 0.999887727 | 4.38906 | 4.20703 | 3.68077 | 4.58914 | 4.2095 | 4.14416 | CGNC:4743,GeneID:426885 |
| HDAC8 | 875.8065157 | 0.106743006 | -1.487236491 | 0.895799587 | -0.158752093 | 0.136952362 | 0.999887727 | 26.3641 | 24.601 | 25.9058 | 24.7824 | 21.4788 | 21.5684 | CGNC:3582,GeneID:422182 |
| HDAC9 | 334.7367219 | 0.155845952 | -0.923389184 | 0.905065139 | -0.143906466 | 0.355804426 | 0.999887727 | 1.4288 | 1.60212 | 1.52137 | 1.39253 | 1.47089 | 1.2965 | CGNC:51296,GeneID:420599 |
| HAT1 | 1610.766724 | 0.088834387 | -0.613483893 | 0.962929135 | -0.054498466 | 0.539556429 | 0.999887727 | 49.8679 | 43.3347 | 52.5138 | 45.1808 | 44.1237 | 50.0781 | CGNC:7250,GeneID:374037 |
| SIRT2 | 1033.166208 | 0.105558258 | -0.11836603 | 0.991376859 | -0.012494512 | 0.90577764 | 0.999887727 | 37.9947 | 41.1669 | 43.7349 | 41.534 | 41.1576 | 35.9589 | CGNC:54341,GeneID:548628 |
| SIRT3 | 463.0340909 | 0.139902338 | -0.07939474 | 0.992330423 | -0.01110751 | 0.936718652 | 0.999887727 | 10.0007 | 11.1584 | 10.9784 | 11.505 | 11.8751 | 9.01747 | CGNC:3086,GeneID:422988 |
| SIRT6 | 473.6992694 | 0.136178922 | -0.699710371 | 0.936086786 | -0.095285804 | 0.484108198 | 0.999887727 | 17.2229 | 15.3952 | 17.2778 | 17.2634 | 13.8199 | 14.9014 | CGNC:850,GeneID:428332 |
| SIRT7 | 283.3898614 | 0.168521074 | -0.505142359 | 0.94270146 | -0.085127133 | 0.613458852 | 0.999887727 | 7.98319 | 7.94346 | 8.6584 | 7.44527 | 7.3086 | 6.90346 | CGNC:71990,GeneID:10321419 |
| MORF4L1 | 3554.381506 | 0.074820981 | -0.465945394 | 0.976124794 | -0.034862491 | 0.641254587 | 0.999887727 | 75.9043 | 71.4445 | 75.9753 | 71.4873 | 70.5611 | 74.0132 | CGNC:54344,GeneID:554283 |
| E6.5 | | | | | | | | | | | | | | |
| HDAC1 | 1679.781325 | 0.099975715 | 0.649175718 | 1.046013743 | 0.064901807 | 0.516224805 | 0.897823318 | 21.9378 | 22.5004 | 21.9751 | 25.8953 | 21.114 | 23.4623 | CGNC:2394,GeneID:373961 |
| HDAC10 | 744.5441011 | 0.123889985 | -2.506530711 | 0.806343214 | -0.310534052 | 0.012192247 | 0.1944044 | 9.99407 | 9.79107 | 7.83736 | 7.50638 | 8.72201 | 6.72991 | CGNC:50586,GeneID:417742 |
| HDAC11 | 569.6749639 | 0.133684908 | -1.957889817 | 0.834081162 | -0.261740321 | 0.050242943 | 0.41425929 | 7.38121 | 8.01784 | 7.47471 | 6.05309 | 6.72615 | 7.02483 | CGNC:3790,GeneID:415978 |
| HDAC2 | 5824.715881 | 0.07840688 | 0.792810605 | 1.044029014 | 0.062161806 | 0.427888177 | 0.863167032 | 74.3259 | 76.1538 | 70.6366 | 82.7267 | 77.1329 | 77.739 | CGNC:11140,GeneID:395635 |
| HDAC3 | 1038.174514 | 0.113133774 | 1.011300494 | 1.082533928 | 0.114412242 | 0.31187263 | 0.801275633 | 21.5067 | 19.103 | 22.4107 | 25.0258 | 23.0452 | 22.7675 | CGNC:1896,GeneID:395506 |
| HDAC4 | 840.5473112 | 0.119637088 | -1.913390853 | 0.853277842 | -0.228912511 | 0.055698035 | 0.434173129 | 4.29716 | 4.39897 | 3.82542 | 3.39724 | 3.87594 | 3.68702 | CGNC:49084,GeneID:374207 |
| HDAC7 | 720.8888626 | 0.12608891 | 0.399261262 | 1.035510669 | 0.050342417 | 0.689700707 | 0.951353357 | 4.11374 | 4.27205 | 3.87825 | 4.66627 | 3.72448 | 4.5109 | CGNC:4743,GeneID:426885 |
| HDAC8 | 1053.721504 | 0.113721844 | 0.683494548 | 1.055354917 | 0.07772826 | 0.494294394 | 0.889233037 | 25.5533 | 25.6578 | 26.4923 | 29.1835 | 23.7545 | 29.7363 | CGNC:3582,GeneID:422182 |
| HDAC9 | 304.7327875 | 0.167571545 | -0.202602034 | 0.976742152 | -0.033950336 | 0.8394461 | 0.980006896 | 1.28418 | 1.04094 | 1.20799 | 1.17378 | 1.10805 | 1.17606 | CGNC:51296,GeneID:420599 |
| HAT1 | 1524.034232 | 0.097957311 | 0.742914834 | 1.051736966 | 0.072773939 | 0.457533244 | 0.875556365 | 41.2658 | 38.5743 | 37.1607 | 43.4114 | 39.617 | 41.4848 | CGNC:7250,GeneID:374037 |
| SIRT2 | 1329.120066 | 0.112484488 | 0.581452694 | 1.046378214 | 0.065404409 | 0.560935392 | 0.909408551 | 44.6325 | 47.8657 | 51.4141 | 55.8324 | 48.4614 | 47.3267 | CGNC:54341,GeneID:548628 |
| SIRT3 | 568.856599 | 0.130956264 | 1.474845776 | 1.143249503 | 0.193140292 | 0.140254011 | 0.629596131 | 10.4076 | 12.9263 | 13.127 | 13.9153 | 14.0116 | 14.8962 | CGNC:3086,GeneID:422988 |
| SIRT6 | 511.4975549 | 0.136312697 | 0.629194059 | 1.061251902 | 0.085767139 | 0.529222018 | 0.899744421 | 14.9138 | 14.7383 | 16.54 | 17.9368 | 16.1853 | 16.3325 | CGNC:850,GeneID:428332 |
| SIRT7 | 270.4957424 | 0.176531942 | -0.740916003 | 0.913327805 | -0.130795341 | 0.458744372 | 0.875868302 | 6.63485 | 7.59066 | 7.31509 | 5.97711 | 7.18788 | 7.49856 | CGNC:71990,GeneID:10321419 |
| MORF4L1 | 3845.10284 | 0.083977058 | 0.206676347 | 1.012102967 | 0.017356072 | 0.836262631 | 0.979735773 | 72.4872 | 74.7968 | 70.1295 | 80.1133 | 71.0285 | 73.9919 | CGNC:54344,GeneID:554283 |
| E18.5 | | | | | | | | | | | | | | |
| HDAC1 | 1996.437101 | 0.157535894 | 0.948113383 | 1.109078813 | 0.14936189 | 0.343071736 | 0.532967005 | 23.8362 | 27.109 | 26.5518 | 26.3596 | 26.5163 | 28.6552 | CGNC:2394,GeneID:373961 |
| HDAC10 | 964.7102626 | 0.1879833 | -2.780694723 | 0.696056262 | -0.522724171 | 0.005424272 | 0.021551675 | 12.8219 | 14.6143 | 12.0536 | 8.20028 | 8.0983 | 10.5854 | CGNC:50586,GeneID:417742 |
| HDAC11 | 1103.259797 | 0.268515017 | -4.473337431 | 0.434925958 | -1.201158277 | 7.70081E-06 | 7.02465E-05 | 30.432 | 17.5916 | 14.6504 | 6.71768 | 8.48025 | 9.05216 | CGNC:3790,GeneID:415978 |
| HDAC2 | 3262.739879 | 0.165756186 | -0.900464874 | 0.901714344 | -0.149257623 | 0.36787291 | 0.557516645 | 45.0513 | 49.1869 | 42.1858 | 37.9411 | 37.8265 | 43.6086 | CGNC:11140,GeneID:395635 |
| HDAC3 | 1126.953788 | 0.176465338 | 1.506880341 | 1.202396028 | 0.265912149 | 0.131841331 | 0.271358989 | 23.0238 | 18.444 | 21.8148 | 24.4838 | 25.2049 | 25.2448 | CGNC:1896,GeneID:395506 |
| HDAC4 | 1266.310608 | 0.164866119 | 2.396040018 | 1.314966293 | 0.395025819 | 0.016573281 | 0.053947481 | 5.57199 | 5.19702 | 5.21273 | 7.03884 | 6.76756 | 6.29142 | CGNC:49084,GeneID:374207 |
| HDAC7 | 1138.146473 | 0.167888409 | 0.597026411 | 1.071947176 | 0.100233814 | 0.550489748 | 0.718629304 | 6.4649 | 6.64494 | 7.02657 | 6.88006 | 6.2498 | 6.73555 | CGNC:4743,GeneID:426885 |
| HDAC8 | 2126.293504 | 0.169199902 | -4.239581359 | 0.608219194 | -0.717336749 | 2.23937E-05 | 0.000181338 | 76.2742 | 73.7007 | 59.7461 | 40.0723 | 40.8539 | 38.6 | CGNC:3582,GeneID:422182 |
| HDAC9 | 213.7266075 | 0.270208588 | 0.009127631 | 1.001711016 | 0.002466364 | 0.992717305 | 0.996418022 | 0.853166 | 0.943698 | 0.54967 | 0.854276 | 0.824545 | 0.81277 | CGNC:51296,GeneID:420599 |
| HAT1 | 1015.80042 | 0.181750312 | -2.39711545 | 0.739346993 | -0.43567648 | 0.016524716 | 0.053832787 | 32.3089 | 31.1118 | 27.7819 | 21.4267 | 23.1637 | 24.0617 | CGNC:7250,GeneID:374037 |
| SIRT2 | 1617.994467 | 0.176623385 | -1.772724426 | 0.804907783 | -0.31310459 | 0.076274382 | 0.180861975 | 57.6197 | 68.2453 | 80.1469 | 48.917 | 52.6165 | 50.1319 | CGNC:54341,GeneID:548628 |
| SIRT3 | 625.6848995 | 0.186176042 | 0.448637197 | 1.059604222 | 0.083525498 | 0.653693396 | 0.795571554 | 11.1415 | 11.9974 | 11.7747 | 11.3185 | 11.5663 | 12.0652 | CGNC:3086,GeneID:422988 |
| SIRT6 | 718.703009 | 0.179890662 | -0.305935364 | 0.962571147 | -0.055034915 | 0.759653859 | 0.865031937 | 21.788 | 24.4854 | 23.7233 | 20.8341 | 21.3409 | 20.1179 | CGNC:850,GeneID:428332 |
| SIRT7 | 455.5779437 | 0.210075477 | -0.358518845 | 0.949134194 | -0.075316017 | 0.719955068 | 0.839425257 | 9.56574 | 11.6968 | 13.2515 | 9.60044 | 9.80976 | 9.85116 | CGNC:71990,GeneID:10321419 |
| MORF4L1 | 4345.565181 | 0.161133568 | -1.976020876 | 0.801956955 | -0.318403294 | 0.048152413 | 0.126642058 | 112.363 | 96.0046 | 88.7158 | 72.935 | 74.4986 | 74.1686 | CGNC:54344,GeneID:554283 |
