# Supplementary material for: Key events in the process of sex determination and differentiation in early chicken embryos
Source: Anim Biosci. 2025 Feb 27;38(6):1081–104. doi: 10.5713/ab.24.0679 (PMC12061580; doi:10.5713/ab.24.0679)
Supplement: Supplementary file 18 [file ab-24-0679-Supplementary-18.pdf]

Supplement 18. Methylation related genes partial enrichment terms.

Term_ID	Term_description	ListHits	FoldEnrichmen	p-value	q-value	geneID
gga00310	Lysine degradation	3	47.0471698	2.22E-05		PRDM6;SETD7;SMYD1
gga04068	FoxO signaling pathway	2	13.6256831	0.008348736		SETD7;RAG2
gga00140	Steroid hormone biosynthesis	1	22.463964	0.043719692		CYP3A5
GO:0018024	histone-lysine N-methyltransferase activity	3	126.070588	1.58E-06	0.00016145	PRDM6;SETD7;SMYD1
GO:0051569	regulation of histone H3-K4 methylation	2	336.188235	1.33E-05	0.00090861	GATA3;GFI1
GO:0042054	histone methyltransferase activity	1	168.094118	0.005935737	0.035689	PRDM8
GO:0016571	histone methylation	1	140.078431	0.007118899	0.035689	PRDM8
GO:0051571	positive regulation of histone H3-K4 methylation	1	105.058824	0.009481246	0.035689	GCG
GO:0080182	histone H3-K4 trimethylation	1	84.0470588	0.011838303	0.035689	TET2
GO:0035097	histone methyltransferase complex	1	49.4394464	0.020046496	0.04501516	PRDM8
GO:0043046	DNA methylation involved in gamete generation	3	180.10084	5.05E-07	0.00010356	FKBP6;TDRKH;TDRD5
GO:0080111	DNA demethylation	1	140.078431	0.007118899	0.035689	TET2
GO:0044030	regulation of DNA methylation	1	105.058824	0.009481246	0.035689	PRDM14
GO:0008584	male gonad development	1	19.5458276	0.049975694	0.06922309	GATA3
GO:0043627	response to estrogen	2	84.0470588	0.000249999	0.00569441	GATA3;CYP3A5
GO:0032355	response to estradiol	1	21.0117647	0.046566759	0.06791244	CYP3A5
GO:0035774	positive regulation of insulin secretion involved in cellular response to glucose stimulus	1	44.2352941	0.022379882	0.04510884	GCG
