# Supplementary material for: Key events in the process of sex determination and differentiation in early chicken embryos
Source: Anim Biosci. 2025 Feb 27;38(6):1081–104. doi: 10.5713/ab.24.0679 (PMC12061580; doi:10.5713/ab.24.0679)
Supplement: Supplementary file 19 [file ab-24-0679-Supplementary-19.pdf]

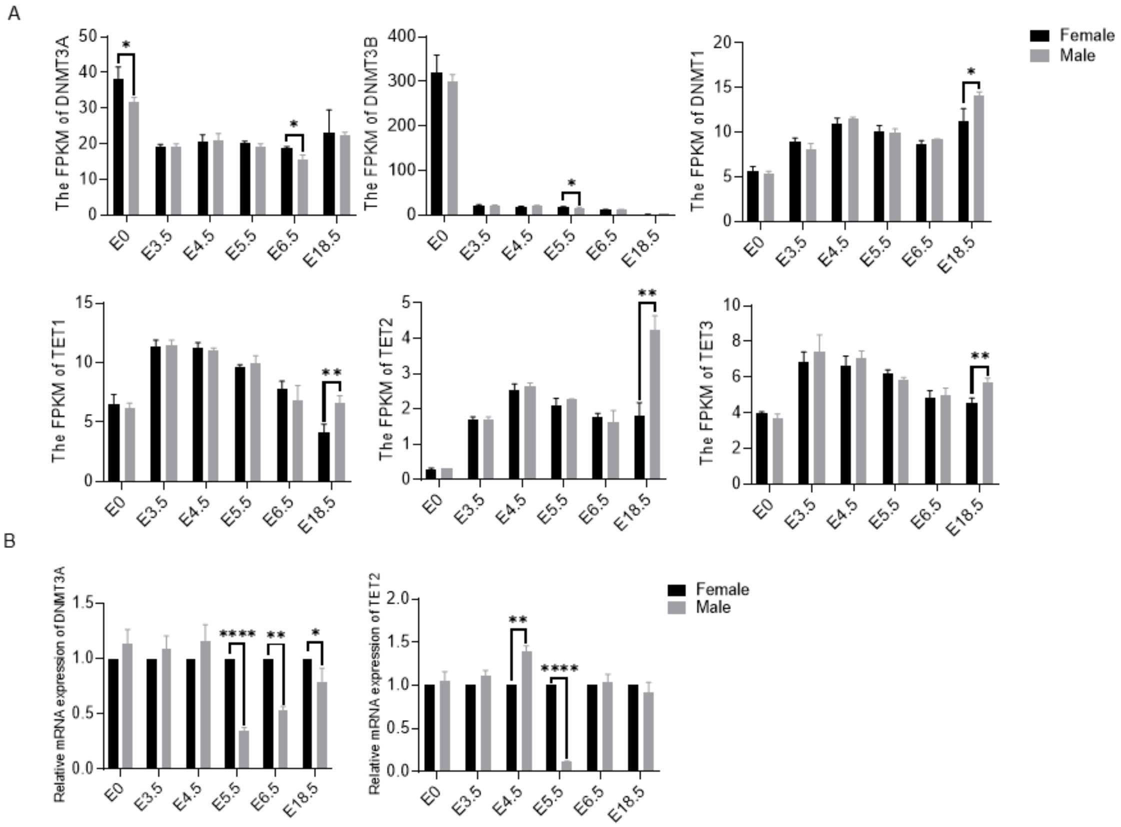

Supplement 19 A. The FPKM value of key enzymes in the DNA methylation process (DNMT3A, DNMT3B, DNMT1, TET1, TET2 and TET3) at E0-E18.5. *p < 0.05, significant difference; **p < 0.01, extremely significant difference. B. The relative expression level of gender-related genes (DNMT3A and TET2) at E0-E18.5 were detected by qRT-PCR.
