# Supplementary material for: Key events in the process of sex determination and differentiation in early chicken embryos
Source: Anim Biosci. 2025 Feb 27;38(6):1081–104. doi: 10.5713/ab.24.0679 (PMC12061580; doi:10.5713/ab.24.0679)
Supplement: Supplementary file 20 [file ab-24-0679-Supplementary-20.pdf]

Supplement 20. Distribution statistics of GO items related to DNA/histone methylation and corresponding related genes in different periods in male and female.

id	term	category	ListHits	ListTotal	PopHits	PopTotal	pval	padj	Enrichment_score	Gene
E0										
GO:0018024	histone-lysine N-methyltransferase activity	molecular_function	1	69	24	14405	0.005830423	0.009191491	8.698671498	PRDM6
E4.5										
GO:0051571	positive regulation of histone H3-K4 methylation	biological_process	1	46	9	14405	0.000354065	0.000799935	34.79468599	GCG
E6.5										
GO:0051567	histone H3-K9 methylation	biological_process	1	102	3	14405	0.000148263	0.000721932	47.0751634	PRDM8
GO:0046974	histone methyltransferase activity (H3-K9 specific)	molecular_function	1	102	5	14405	0.000489648	0.001720696	28.24509804	PRDM8
GO:0042054	histone methyltransferase activity	molecular_function	1	102	7	14405	0.001018781	0.002896915	20.17507003	PRDM8
GO:0016571	histone methylation	biological_process	1	102	9	14405	0.001730398	0.00425461	15.69172113	PRDM8
GO:0035097	histone methyltransferase complex	cellular_component	1	102	14	14405	0.004274241	0.008424099	10.08753501	PRDM8
E18.5										
GO:0061085	regulation of histone H3-K27 methylation	biological_process	1	2499	1	14405	0	0	5.764305722	GATA3
GO:0034971	histone H3-R17 methylation	biological_process	1	2499	1	14405	0	0	5.764305722	NR1H4
GO:0034972	histone H3-R26 methylation	biological_process	1	2499	1	14405	0	0	5.764305722	PRDM14
GO:0051570	regulation of histone H3-K9 methylation	biological_process	1	2499	1	14405	0	0	5.764305722	SETD7
GO:0006211	5-methylcytosine catabolic process	biological_process	1	2499	1	14405	0	0	5.764305722	TET2
GO:0090308	regulation of methylation-dependent chromatin silencing	biological_process	1	2499	1	14405	0	0	5.764305722	UHRF2
GO:0051569	regulation of histone H3-K4 methylation	biological_process	2	2499	4	14405	0.018151652	0.05976976	2.882152861	GATA3; GFI1
GO:1901536	negative regulation of DNA demethylation	biological_process	1	2499	2	14405	0.030085852	0.070274844	2.882152861	GATA3
GO:0010216	maintenance of DNA methylation	biological_process	1	2499	2	14405	0.030085852	0.070274844	2.882152861	UHRF2
GO:0070989	oxidative demethylation	biological_process	2	2499	5	14405	0.0395391	0.086681368	2.305722289	CYP1A2; CYP3A5
GO:0043046	DNA methylation involved in gamete generation	biological_process	4	2499	14	14405	0.07953729	0.138156799	1.646944492	FKBP6; LOC107049165; TDRD5; TDRKH
GO:0042799	histone methyltransferase activity (H4-K20 specific)	molecular_function	1	2499	3	14405	0.079825789	0.138156799	1.921435241	KMT5C
GO:0016428	tRNA (cytosine-5-)-methyltransferase activity	molecular_function	1	2499	3	14405	0.079825789	0.138156799	1.921435241	NSUN6
GO:0051567	histone H3-K9 methylation	biological_process	1	2499	3	14405	0.079825789	0.138156799	1.921435241	PRDM8
GO:0016571	histone methylation	biological_process	2	2499	9	14405	0.194438073	0.281466166	1.280956827	PRDM8; SATB1
GO:0046974	histone methyltransferase activity (H3-K9 specific)	molecular_function	1	2499	5	14405	0.209473809	0.288310387	1.152861144	PRDM8
GO:0034773	histone H4-K20 trimethylation	biological_process	1	2499	6	14405	0.279707589	0.362029371	0.96071762	KMT5C
GO:0032259	methylation	biological_process	2	2499	11	14405	0.29515248	0.378164116	1.048055586	ARMT1; GSTO1
GO:0051568	histone H3-K4 methylation	biological_process	1	2499	7	14405	0.34937263	0.429941971	0.823472246	DYDC1
GO:0042054	histone methyltransferase activity	molecular_function	1	2499	7	14405	0.34937263	0.429941971	0.823472246	PRDM8
GO:0044030	regulation of DNA methylation	biological_process	1	2499	8	14405	0.41655309	0.49677813	0.720538215	PRDM14
GO:0051571	positive regulation of histone H3-K4 methylation	biological_process	1	2499	9	14405	0.480014524	0.557946095	0.640478414	GCG
GO:0080111	DNA demethylation	biological_process	1	2499	9	14405	0.480014524	0.557946095	0.640478414	TET2
GO:0080182	histone H3-K4 trimethylation	biological_process	1	2499	10	14405	0.53902526	0.614313836	0.576430572	TET2
GO:0018024	histone-lysine N-methyltransferase activity	molecular_function	3	2499	24	14405	0.618893365	0.689155719	0.720538215	KMT5C; SETD7; SMYD1
GO:0008757	S-adenosylmethionine-dependent methyltransferase activity	molecular_function	1	2499	14	14405	0.726695183	0.78316978	0.411736123	ARMT1
GO:0035097	histone methyltransferase complex	cellular_component	1	2499	14	14405	0.726695183	0.78316978	0.411736123	PRDM8
GO:0008168	methyltransferase activity	molecular_function	1	2499	32	14405	0.982727613	0.996924078	0.180134554	PRDM14
GO:0035064	methylated histone binding	molecular_function	1	2499	34	14405	0.987568526	0.999684073	0.169538404	RAG2

Supplement 20-1. The FPKM values of methylation related differentially expressed genes during different developmental stages.

gene_id	baseMean	lfcSE	stat	foldChange	log2FoldChange	pval	padj	up_down	expression_Female1	expression_Female2	expression_Female3	expression_Male1	expression_Male2	expression_Male3	Dbxref
E0															
PRDM6	27.11350842	0.531561039	3.450066644	3.565046787	1.833921011	0.000560448	0.023504803	Up	0.263475	0.10916	0.103923	0.708993	0.469155	0.480789	CGNC:3984,GeneID:426800
E4.5															
GCG	34.89753552	0.483327027	2.494792321	2.306652306	1.205800555	0.012603087	0	Up	0.217562	0.186408	0.189405	0.750866	0.39302	0.245994	CGNC:8432,GeneID:396196
E6.5															
PRDM8	30.43606422	0.569680934	3.041906953	3.32399081	1.732916394	0.002350846	0.061668371	Up	0.439868	0.438875	1.39438	2.25965	2.0734	1.0851	CGNC:58878,GeneID:777386
E18.5															
GATA3	39.20737637	0.551651136	-4.334348839	0.190643803	-2.391048459	1.46192E-05	0.000124564	Down	0.74262	0.848998	0.76227	0.189228	0.118463	0.126845	CGNC:5080,GeneID:419106
NR1H4	19.38042438	0.835829661	-3.032462975	0.172584822	-2.534622501	0.002425669	0.010873106	Down	0.421599	0.690443	0.576487	0.140224	0.0210299	0.145172	CGNC:8812,GeneID:373902
PRDM14	82.02233398	0.520845565	-9.224690106	0.035781584	-4.804638929	2.84382E-20	2.3203E-18	Down	0.872194	0.754205	0.639791	0.0252733	0.0267164	0.0263671	CGNC:64389,GeneID:100858709
SETD7	1391.303585	0.174354531	5.944999149	2.051298631	1.036537537	2.76458E-09	5.39394E-08	Up	7.22366	6.57979	7.74374	15.7584	14.164	14.04	CGNC:7445,GeneID:422443
TET2	1868.091605	0.215609216	5.780429609	2.372345111	1.246313898	7.45101E-09	1.31986E-07	Up	2.0732	1.38011	1.95796	3.75627	4.36911	4.5424	CGNC:51773,GeneID:422540
UHRF2	2367.688018	0.219596782	-4.93745547	0.471637608	-1.084249332	7.91485E-07	9.27898E-06	Down	27.9966	33.7057	20.8065	11.5387	12.1965	14.3776	CGNC:11194,GeneID:431601
CYP1A2	28.42851365	0.837607926	-2.802305685	0.19652252	-2.347233454	0.005073877	0.0203851	Down	0.316922	0.212153	2.67028	0.161648	0.0993698	0.0940811	CGNC:49932,GeneID:396051
FKBP6	340.8429863	0.322745117	-3.451578165	0.462017692	-1.113979998	0.000557318	0.003101439	Down	5.63505	4.03647	2.51379	1.93396	1.34836	1.99921	CGNC:50514,GeneID:417460
KMT5C	818.9419996	0.221446574	-7.657195235	0.308713473	-1.695659649	1.90038E-14	8.0368E-13	Down	40.6149	57.7921	39.3086	11.2781	11.3314	13.7984	CGNC:67572,GeneID:101748081
NSUN6	157.2417667	0.296832817	-3.888391305	0.449314603	-1.154202143	0.000100911	0.000695187	Down	3.13828	2.54898	2.20556	1.43102	0.977264	1.08085	CGNC:6499,GeneID:428419
PRDM8	5.300953844	1.804632087	-2.708827392	0.033762444	-4.888436831	0.006752146	0.025830195	Down	0.410243	0.497311	0.683256	0	0.0558324	0	CGNC:58878,GeneID:777386
ARMT1	1944.239591	0.212011625	-7.277847352	0.343173905	-1.542988242	3.39189E-13	1.19904E-11	Down	33.4796	28.9452	16.7045	10.1542	8.12041	8.98035	CGNC:9819,GeneID:421637
DYDC1	153.3421129	0.693382656	-10.45745813	0.00656486	-7.251020096	1.3544E-25	1.91933E-23	Down	8.83734	12.7236	9.64289	0.0430522	0	0.0908144	CGNC:1735,GeneID:423627
GCG	151.0612144	0.310532592	-4.970873183	0.343024105	-1.543618136	6.6652E-07	7.97012E-06	Down	1.54878	2.09002	1.59023	0.708197	0.744664	0.853236	CGNC:8432,GeneID:396196
RAG2	112.6293534	0.334919389	-6.114187007	0.241859351	-2.04775978	9.70504E-10	2.0468E-08	Down	1.11501	0.647464	0.673136	0.213596	0.139923	0.14375	CGNC:6005,GeneID:423165
GFI1	255.505304	0.282512162	-9.368691834	0.159677243	-2.646769381	7.3437E-21	6.41284E-19	Down	8.90222	7.50049	5.21049	1.37236	0.915041	1.16053	CGNC:4440,GeneID:429088
CYP3A5	16.365044	1.022165152	-2.134864209	0.220341966	-2.1821838	0.032772104	0.092980916	Down	0.703584	0.901897	0.174362	0.248994	0	0.0580109	CGNC:49925,GeneID:414832
LOC107049165	1482.697477	0.239275019	-6.453339705	0.34290409	-1.544122983	1.09412E-10	2.66197E-09	Down	54.1528	46.8369	30.2887	12.9571	11.8497	13.9158	CGNC:72186,GeneID:107049165
TDRKH	1871.36812	0.235686484	-8.522387313	0.248512186	-2.008611497	1.56298E-17	9.63736E-16	Down	65.232	56.6986	37.7947	11.8954	10.2909	12.0482	CGNC:16943,GeneID:425667
TDRD5	778.8966557	0.238949965	-4.616584608	0.465504581	-1.103132729	3.90107E-06	3.8038E-05	Down	16.6487	11.192	8.30486	5.87407	5.30304	5.4181	CGNC:3034,GeneID:424421
SATB1	241.3727966	0.271544904	6.378328932	3.321886355	1.732002718	1.79031E-10	4.22842E-09	Up	0.491126	0.619192	1.23027	2.17105	2.27523	3.04277	CGNC:8550,GeneID:420647
GSTO1	383.2042937	0.253514279	-12.28407076	0.115487824	-3.114187346	1.10305E-34	2.82854E-32	Down	23.4431	30.9614	17.7347	1.91755	3.64301	2.13661	CGNC:52072,GeneID:423881
SMYD1	72.7490822	0.673246225	-7.973824271	0.024208423	-5.368347093	1.53838E-15	7.55397E-14	Down	1.83293	1.96825	1.19289	0.0975165	0.0345964	0	CGNC:11893,GeneID:373960
