# Supplementary material for: Key events in the process of sex determination and differentiation in early chicken embryos
Source: Anim Biosci. 2025 Feb 27;38(6):1081–104. doi: 10.5713/ab.24.0679 (PMC12061580; doi:10.5713/ab.24.0679)
Supplement: Supplementary file 21 [file ab-24-0679-Supplementary-21.pdf]

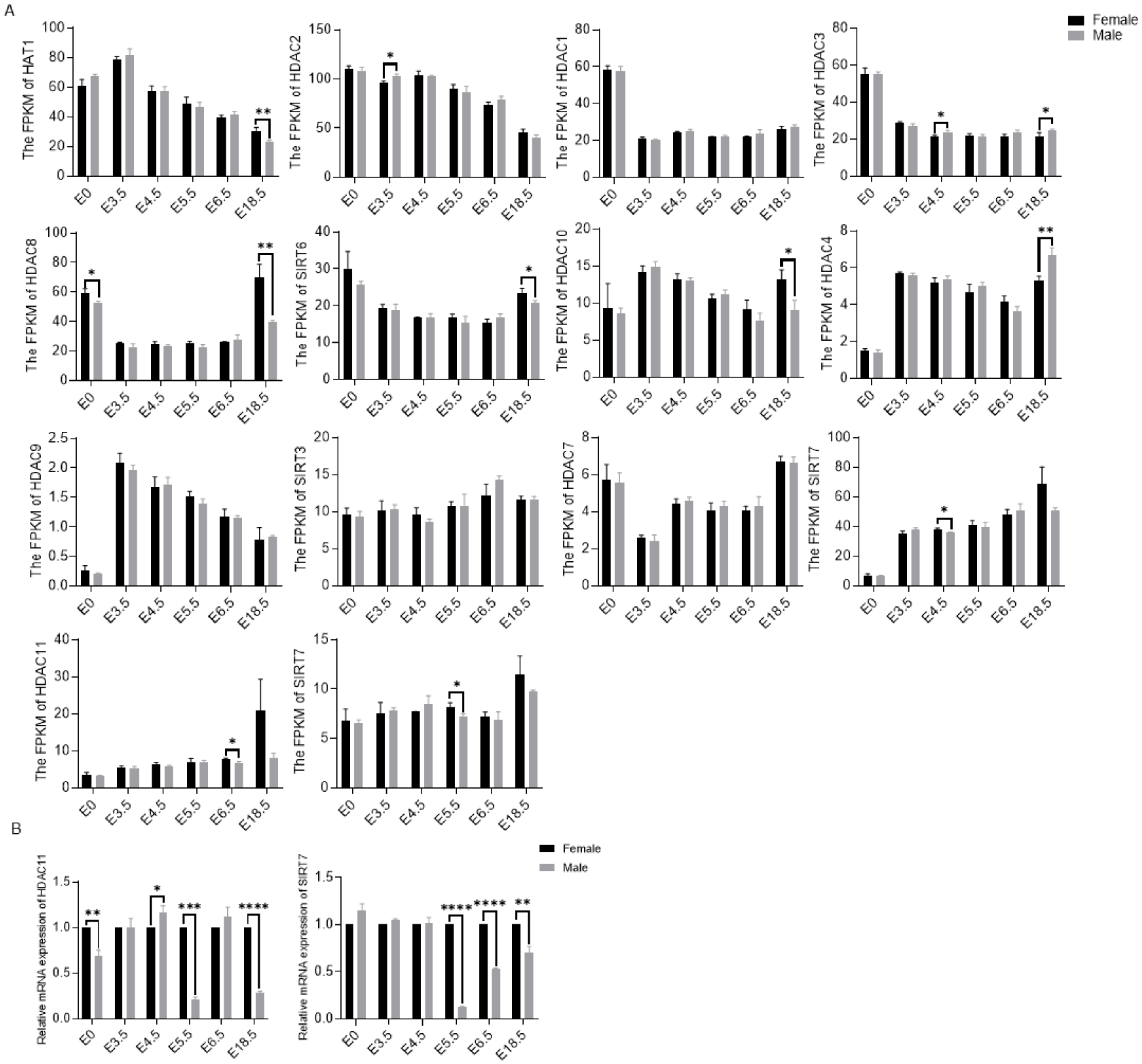

Supplement 21. A. The FPKM value of key enzymes in histone acetylation (HAT1, HDAC2, HDAC1, HDAC3, HDAC8, SIRT6, HDAC10, HDAC4, HDAC9, SIRT3, HDAC7, SIRT7,HDAC11 and SIRT7) at E0-E18.5. *p < 0.05, significant difference; **p < 0.01, extremely significant difference. B. The relative expression level of gender-related genes (HDAC11 and SIRT7) at E0-E18.5 were detected by qRT-PCR.
