# Supplementary material for: Key events in the process of sex determination and differentiation in early chicken embryos
Source: Anim Biosci. 2025 Feb 27;38(6):1081–104. doi: 10.5713/ab.24.0679 (PMC12061580; doi:10.5713/ab.24.0679)
Supplement: Supplementary file 22 [file ab-24-0679-Supplementary-22.pdf]

Supplement 22. The original analysis process of Dot blot.

		Area	Mean	IntDen	RawIntDen	Female/Male	Male/Male	The mean value of Female/Male	The mean value of Male/Male	Student's t test
Repeat1										
300ng-Female	1	2299	160.649	369333	369333	1.458148597	1	1.493010017	1	0.020077175
300ng-Male	2	2299	110.174	253289	253289					
200ng-Female	3	2299	115.784	266188	266188	1.501503263	1			
200ng-Male	4	2299	77.112	177281	177281					
100ng-Female	5	2299	30.95	71154	71154	1.51937819	1			
100ng-Male	6	2299	20.37	46831	46831					
Repeat2		Area	Mean	IntDen	RawIntDen					
300ng-Female	1	5413	120.741	653570	653570	1.007707714	1	1.173550318	1	
300ng-Male	2	5413	119.817	648571	648571					
200ng-Female	3	5413	121.98	660278	660278	1.441393809	1			
200ng-Male	4	5413	84.626	458083	458083					
100ng-Female	5	5413	69.611	376805	376805	1.071549432	1			
100ng-Male	6	5413	64.963	351645	351645					
Repeat3		Area	Mean	IntDen	RawIntDen					
300ng-Female	1	6020	122.481	737334	737334	1.095379651	1	1.391595262	1	
300ng-Male	2	6020	111.816	673131	673131					
200ng-Female	3	6020	107.045	644409	644409	1.357888349	1			
200ng-Male	4	6020	78.832	474567	474567					
100ng-Female	5	6020	102.39	616386	616386	1.721517785	1			
100ng-Male	6	6020	59.476	358048	358048					
